# Wet-processed high-areal-capacity electrodes via transformative spandex–poly(acrylic acid) binder toward 450 Wh kg^−1^ lithium-ion batteries

**DOI:** 10.1038/s41467-026-76014-4

**Published:** 2026-07-23

**Authors:** Bo Keun Park, Yoon Bo Shim, Jong Uk Won, Sung Joon Park, Yeon Jeong Kim, Byeongjin Park, Jang Wook Choi, Ki Jae Kim

**Affiliations:** 1 Department of Energy Science, Sungkyunkwan University, Suwon-si, South Korea; 2 Department of Future Energy Engineering, Sungkyunkwan University, Suwon-si, South Korea; 3 School of Chemical and Biological Engineering and Institute of Chemical Process, Seoul National University, Seoul, South Korea; 4 SKKU Institute of Energy Science and Technology (SIEST), Sungkyunkwan University, Suwon-si, South Korea

**Keywords:** Batteries, Batteries, Batteries

## Abstract

Driven by the increasing interest in lithium-ion batteries with high energy density, the design of high-mass-loading electrodes with Ni-rich positive materials has recently been considered one of the most promising strategies to achieve this goal. However, the conventional binder, poly(vinylidene fluoride), cannot ensure structural integrity and uniform charge transfer in high-mass-loading electrodes. Herein, we propose a dual-acting hybrid polymer as an advanced wet-processable binder, comprising a crosslinked network of spandex and polyacrylic acid. Spandex imparts high elasticity and strong affinity with Ni-rich positive electrode, while poly(acrylic acid) forms lithium polyacrylate on the electrode surface to enhance interfacial Li^+^ transport. The distinct roles of each polymer ensure mechanical robustness, enhance Li^+^ transport, and suppress binder migration during the drying process, thereby alleviating chronic issues in high-mass-loading electrodes. Notably, proposed hybrid polymer binder enables the fabrication of high-mass-loading electrodes (70 mg cm^−2^) with stable cyclability, despite a low binder content of 2 wt%. Moreover, pouch cell employing high-loading positive electrode based on the hybrid polymer binder exhibited improved cycling stability over its conventional poly(vinylidene fluoride)-based counterparts, ultimately highlighting its industrial applicability. This study provides practical insights into rational design of binders and highlights their potential to enable wet-processable fabrication of high-mass-loading electrodes.

## Abstract (web summary)

High-areal-loading electrodes are needed for high-energy-density batteries. Here, the authors proposed a Spandex/PAA hybrid polymer binder that enables mechanically robust thick electrodes with fast lithium-ion transport in a conventional wet-processed system.

## Introduction

Over the past few decades, lithium-ion batteries (LIBs) have been continuously advanced to achieve higher energy densities. However, further development is required to achieve even higher energy densities to serve as long-term power sources for electric vehicles (EV), energy storage systems (ESS), and urban air mobility (UAM)^1–6^. To achieve this, new candidates for positive and negative materials with higher theoretical capacities need to be developed, and significant efforts are in progress to make this possible. Considering negative electrodes, the development of next-generation materials such as silicon or lithium metal, which can replace commercial graphite materials, is rapidly advancing^7–10^. Efforts are also ongoing to develop next-generation positive materials beyond Ni-rich positive electrodes^11,12^. However, as safety concerns regarding Ni-rich positive electrodes have become a key issue, the focus has shifted toward developing safer positive materials rather than increasing their specific capacity^13,14^. Consequently, increasing the loading level of existing Ni-rich positive materials has been investigated, and this approach has proven to be very effective in developing high-energy-density LIBs^15,16^.

The main challenges associated with thick electrodes primarily stem from binder migration during the drying process and limited electrolyte penetration caused by the increased electrode thickness^17–20^. Binder migration toward the electrode surface is driven by capillary forces generated during solvent evaporation in the drying process and becomes more severe in thicker electrodes owing to prolonged solvent removal. This phenomenon leads to delamination by weakening the adhesion between the electrode and current collector and causes agglomeration of the binder and conductive materials, significantly hindering the lithium-ion and electron conductivity of the electrode^17,18^. Moreover, poor electrolyte penetration, caused by the formation of a thick binder layer on the electrode surface due to binder migration, further impedes lithium-ion mobility within the electrode^19,20^. These issues result in non-uniform electrochemical reactions in the electrode, thereby accelerating the degradation of the top-layer of the Ni-rich positive material such as crack formation, thick cathode electrolyte interface (CEI) layers, structural collapse, and reduced cyclability^21,22^. Therefore, thick electrodes with low tortuosity, uniform dispersion, and high electrolyte permeability are essential for inducing uniform electrochemical reactions. To achieve this, some research has focused on unique fabrication methods such as phase inversion, ice templating, and magnetic templating^10,23–25^. However, these methods often use water or non-reactive additives, which can significantly degrade the Ni-rich positive electrodes and reduce its energy density. These factors may lead to unwanted side reactions and additional processing, further increasing the complexity and costs of manufacturing and making practical industrial applications challenging.

An alternative approach is to develop binders suitable for thick electrodes. Binders play a crucial role in binder migration, the electrode structure, and the deterioration behavior of positive materials^26,27^. Therefore, the development of newly designed binders can effectively address issues of both Ni-rich and thick electrodes without modifications to established manufacturing systems, making them highly promising for industrial-scale applications. Conventional polyvinylidene fluoride (PVDF) is predominantly used as a binder material for positive electrodes owing to its good mechanical and chemical stability. However, the weak adhesion of PVDF, resulting from its van der Waals interactions with active materials, accelerates the structural degradation of Ni-rich positive electrodes, including particle cracking and delamination from the current collector, making it unsuitable for application in thick Ni-rich positive electrodes^26–28^. To overcome these limitations, various studies have explored alternative binders that offer good adhesion, stabilize Ni-rich positive electrodes, and enable the fabrication of robust electrodes at high loading levels^29–34^. Nevertheless, some of these studies have been conducted at relatively low loading levels (<20 mg cm^−2^) and have rarely been extended to thick electrodes ( > 20 mg cm^−2^). In addition, dry processing methods have recently garnered attention for fabricating high-mass-loading electrodes; however, they still face industrial challenges such as increased complexity and cost, suggesting that they are far from completely replacing the conventional wet process^35–38^. Therefore, the development of advanced wet-processable binders that enhance adhesion and Li^+^ mobility while mitigating the degradation of Ni-rich positive electrodes under high-loading conditions is pivotal to achieving high-performance thick Ni-rich positive electrodes without sacrificing the advantages of existing fabrication processes.

**Fig. 1:**
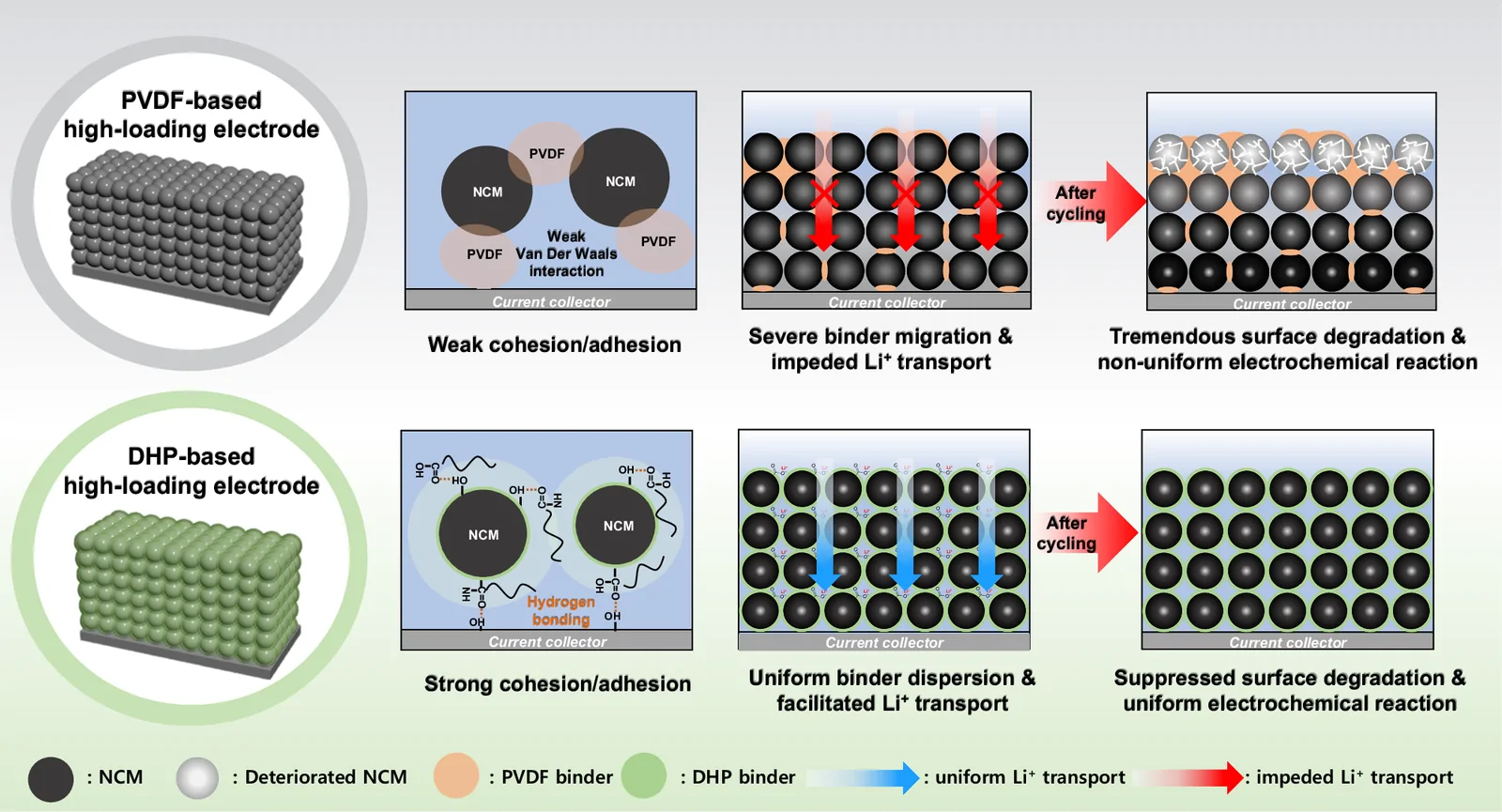
Effects of the DHP binder and a conventional binder on high-loading Ni-rich positive electrodes. The DHP binder improves cohesion/adhesion and Li⁺ transport compared with the PVDF binder, enabling more uniform electrochemical reactions and suppressing surface degradation during cycling.

Here, in an effort to advance the technological maturity of binders for thick electrodes, we report an advanced dual-acting hybrid polymer (DHP) binder to address the manufacturing challenges associated with high-mass-loading positive electrodes and the subsequent degradation of Ni-rich positive materials (LiNi_0.8_Co_0.1_Mn_0.1_, NCM811). The proposed binder was prepared by the simple crosslinking of two polymers with distinct characteristics: spandex (SPDX) and polyacrylic acid (PAA). Specifically, SPDX, which exhibits strong affinity for Ni-rich positive material due to its abundant hydrogen-bonding functional groups and high elasticity, uniformly covers the NCM surface and promotes the uniform dispersion of electrode materials and their robust adhesion/cohesion, as demonstrated in our previous studies conducted at an industry-level mass loading (16.3 mg cm^−2^)^39^. By virtue of these features, it also reinforces the mechanical robustness of the thick electrode (>20.0 mg cm^−2^) and mitigates the surface degradation of thick Ni-rich electrodes. By contrast, PAA facilitates the in situ formation of lithiophilic Li-PAA at the interface between the positive material and electrolyte, thereby enhancing Li^+^ transport and inducing a uniform electrochemical reaction throughout the electrode^40–42^. The synergistic roles of SPDX and PAA as DHP binders are depicted in Fig. 1, and they independently contribute to the mechanical integrity and electrochemical performance of the thick NCM811 electrodes. The fabricated NCM811 positive electrode with DHP binders exhibits a uniform distribution of electrode components in the electrode and enhanced cycling and rate performances (>20.0 mg cm^−2^). Despite the low binder content (2 wt%), the electrode achieves a high mass loading of 70 mg cm^−2^ while maintaining stable cycling performance. To ultimately demonstrate the industrial applicability of the DHP binder, a pouch cell was fabricated using a high-loading electrodes with DHP binder. The resulting cell exhibits improved cyclability compared to that with a conventional PVDF-based counterpart, clearly demonstrating the commercial viability of DHP and effectively overcoming the typical processing challenges associated with high-mass-loading electrodes.

## Results

### Design and synthesis of DHP binders

**Fig. 2:**
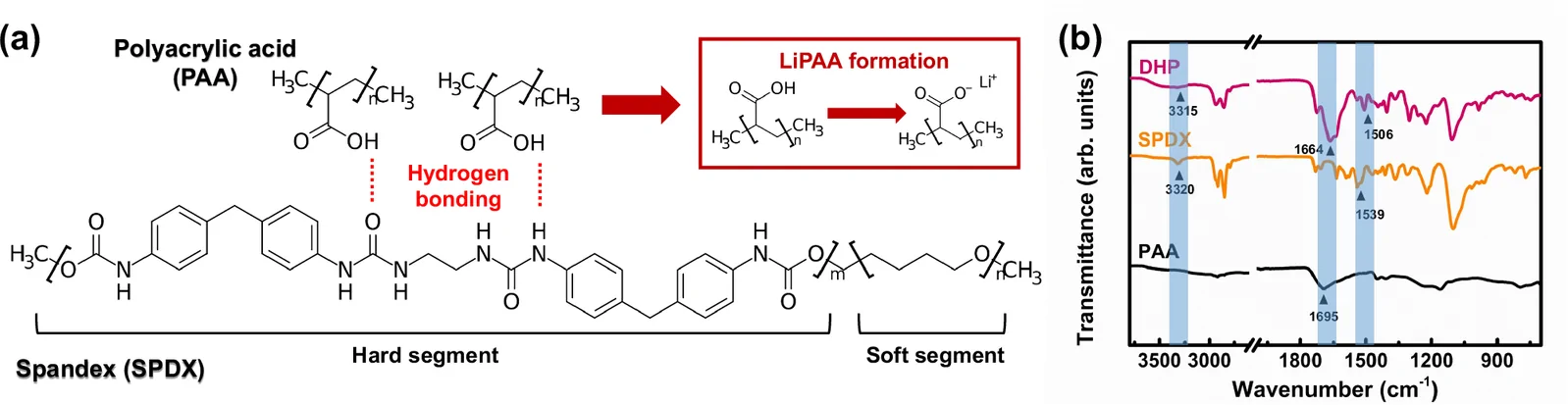
Chemical structures and interactions in the DHP binder. **a** Schematic illustration of SPDX and PAA showing hydrogen bonding interactions and Li–PAA formation through H^+^/Li^+^ exchange in DHP. **b** Fourier transform infrared (FTIR) spectra of PAA, SPDX, and DHP binder films. The blue boxes indicate the main FT-IR peaks shifted by hydrogen bonding.

DHP was synthesized by a straightforward method involving the mechanical stirring of a mixture of SPDX and PAA solutions. Hydrogen bonding is readily formed by the interaction of the unshared electron pairs provided by the urea and urethane functional groups located in the hard segment of SPDX and the carboxyl functional group of PAA (Fig. 2a). To verify the successful synthesis of DHP, various binder materials were fabricated in polymer film form and Fourier-transform infrared spectroscopy (FT-IR) was performed (Fig. 2b). As shown in Fig. 2b, SPDX showed typical peaks at 1539, 1635, and 3320 cm^−1^ corresponding to N-H bending, C = O stretching, and N-H stretching, respectively^43^. PAA exhibited traditional peaks at 1162, 1413, and 1695 cm^−1^ corresponding to C-O stretching, C-O-H in-plane bonding, and C = O stretching, respectively^44^. For DHP, the characteristic peaks of SPDX and PAA were observed, confirming the presence of both polymers. Notably, the C = O stretching peak of PAA shifts from 1695 cm^−1^ to 1664 cm^−1^, and that the corresponding peak of SPDX shifts from 1539 cm^−1^ to 1506 cm^−1^, respectively, in the DHP spectrum. In addition, the N–H stretching vibration of SPDX shifts from 3320 to 3315 cm^−1^ toward a lower vibrational frequency. These shifts indicate successful crosslinking via hydrogen-bonding interactions between the urea and urethane functional groups of SPDX and the carboxyl group of PAA, as illustrated in Fig. 2a^45^. Meanwhile, the urethane, urea, and carboxyl groups, which are abundant in DHP, may form additional hydrogen bonds with the NCM811 particles and the Al current collector, potentially enhancing both cohesion within the electrode and adhesion to the current collector.

The viscosity of the binder solution is an important factor in determining whether a high-mass-loading electrode can be fabricated using a conventional slurry-casting electrode fabrication process. A high-viscosity slurry induces strong shear stress during mixing, which promotes the uniform dispersion of the components and simultaneously enables thick casting without undesired spreading^39,46,47^. To evaluate the effect of the crosslinked structures of SPDX and PAA on viscosity, the rheological properties of the DHP binder solution and comparison groups were examined by measuring the shear viscosity using a rheometer (Fig. S1, Supporting Information). PVDF and PAA exhibited lower viscosities than SPDX and DHP, implying that low-viscosity binders such as PVDF and PAA are not suitable for maintaining the structural stability of high-mass-loading electrodes during conventional slurry-casting processes. By contrast, owing to the high viscosity and shear stress of SPDX, DHP containing the cross-linked structures of both SPDX and PAA showed a relatively high viscosity compared to the PAA-only binder solution. The high viscosity and shear stress of the DHP binder are expected to facilitate the homogeneous dispersion of electrode components during slurry mixing and drying, and it is well suited for the thick casting of the slurry, enabling the formation of a uniformly thick electrode.

### Structural and electrochemical properties of Ni-rich electrode using DHP binders

**Fig. 3:**
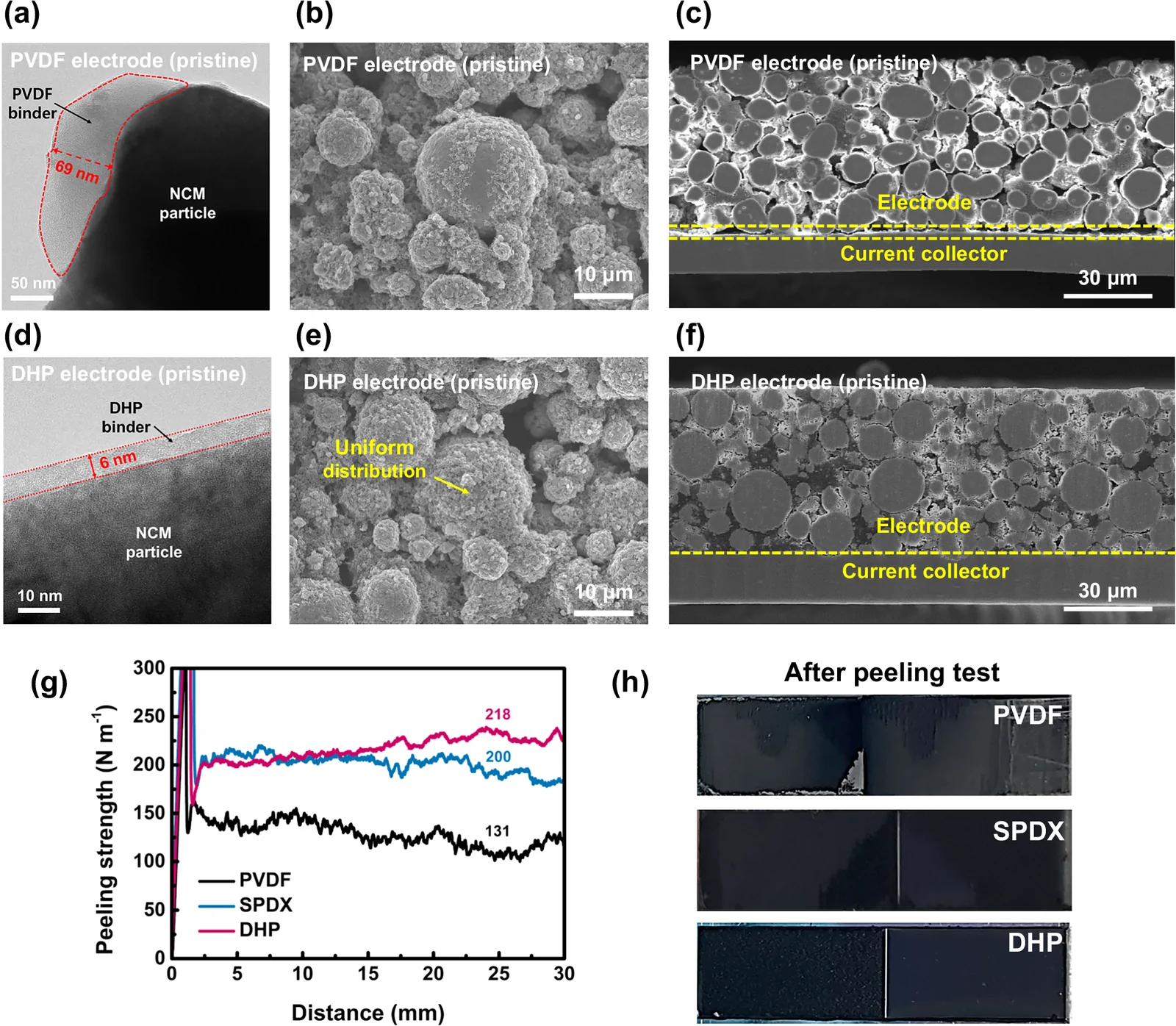
Morphological and mechanical characterization of NCM electrodes. TEM images of the binder coverage on NCM particles in pristine electrodes using **a** PVDF and **d** DHP. Top-view SEM images of pristine electrodes using **b** PVDF and **e** DHP. Cross-sectional SEM images of the pristine electrodes using **c** PVDF and **f** DHP. **g** 180° peeling test curves of the PVDF-, SPDX- and DHP-based electrodes. **h** Photographs after the peel test of the PVDF, SPDX, and DHP electrodes.

The morphologies of the DHP binder and commercial PVDF on the NCM surface were examined using transmission electron microscopy (TEM) analysis. As shown in Fig. 3a, d, the PVDF binder displayed a thick and non-uniform distribution, whereas the DHP binder formed a thin and uniform layer on the NCM811 particle surface. This uniform coverage was attributed to the characteristics of SPDX in the DHP, which formed a thin and uniform layer on the NCM particles, as demonstrated in our previous study^39^. The observed uniform coverage of the DHP binder suggests that its functional groups are more effectively engaged, contributing to enhanced cohesion and adhesion properties and suppressing Ni-rich positive material deterioration. Additionally, top-view scanning electron microscopy (SEM) images of the NCM electrodes with different binders were analyzed to clarify the effect of the binder on the electrode morphology and structure. As shown in Fig. 3b, e, the PVDF electrode exhibited a nonuniform distribution of conductive agents around the NCM surface. By contrast, the DHP electrode showed a uniform distribution of conductive agents on the NCM surface, which was attributed to the uniform covering characteristics and favorable rheological properties of DHP. This well-dispersed distribution of conductive agents in the DHP electrode suggests improved electroconductivity and facilitates a uniform electrochemical reaction throughout the electrode. The cross-sectional SEM images further confirmed the uniform distribution of the electrode components and the well-structured morphology of the NCM electrodes with the DHP binder. The DHP electrode exhibited strong interfacial contact and adhesion between the active material and the current collector because of the uniform dispersibility of the binder and functional groups in DHP, which formed hydrogen bonds with the Al current collector (Fig. 3c). By contrast, the PVDF electrode showed relatively poor adhesion between the active material and current collector, indicating inferior adhesive properties compared to the DHP electrode (Fig. 3f). Furthermore, a comparison of Ni and C EDS mapping images for each electrode revealed that the DHP electrodes achieved a more uniform dispersion of active materials, binders, and conductive materials without binder migration than the PVDF electrode (Fig. S2, Supporting Information). These SEM, TEM, and EDS results collectively demonstrate that the favorable rheological properties and multiple functional groups of the DHP binder lead to a robust electrode with strong adhesion and homogeneous component distribution.

The cohesion and adhesion properties of a binder are largely influenced by its dispersion behavior, degree of surface coverage, and the resulting electrode structure. These properties are crucial factors that determine electrochemical performance, including cell lifespan. To quantify the adhesion strengths of the electrodes prepared using DHP and PVDF, 180° peel tests were conducted using a universal testing machine (Fig. 3g). Electrodes using PVDF, PAA, SPDX, and DHP binders were fabricated with the same composite ratio (active material loading = 20 mg cm^−2^). The DHP electrode exhibited the highest average peeling strength (218 N m^−1^), nearly twice that of the PVDF electrode (131 N m^−1^), and even higher than that of the SPDX electrode (200 N m^−1^), which is well known for its strong adhesion. By contrast, the PAA electrode exhibited poor mechanical properties, with peeling and physical damage occurring after drying at the same loading level of 20 mg cm^−2^ (Fig. S3, Supporting Information). Moreover, unlike the PVDF electrode, in which the Al current collector surface was exposed after the peel test, the SPDX and DHP electrodes maintained strong adhesion (Fig. 3h). These results suggest that the previously confirmed well-constructed electrode structure, combined with the strong interactions of its various functional groups, enhanced the adhesion strength and mechanical integrity.

**Fig. 4:**
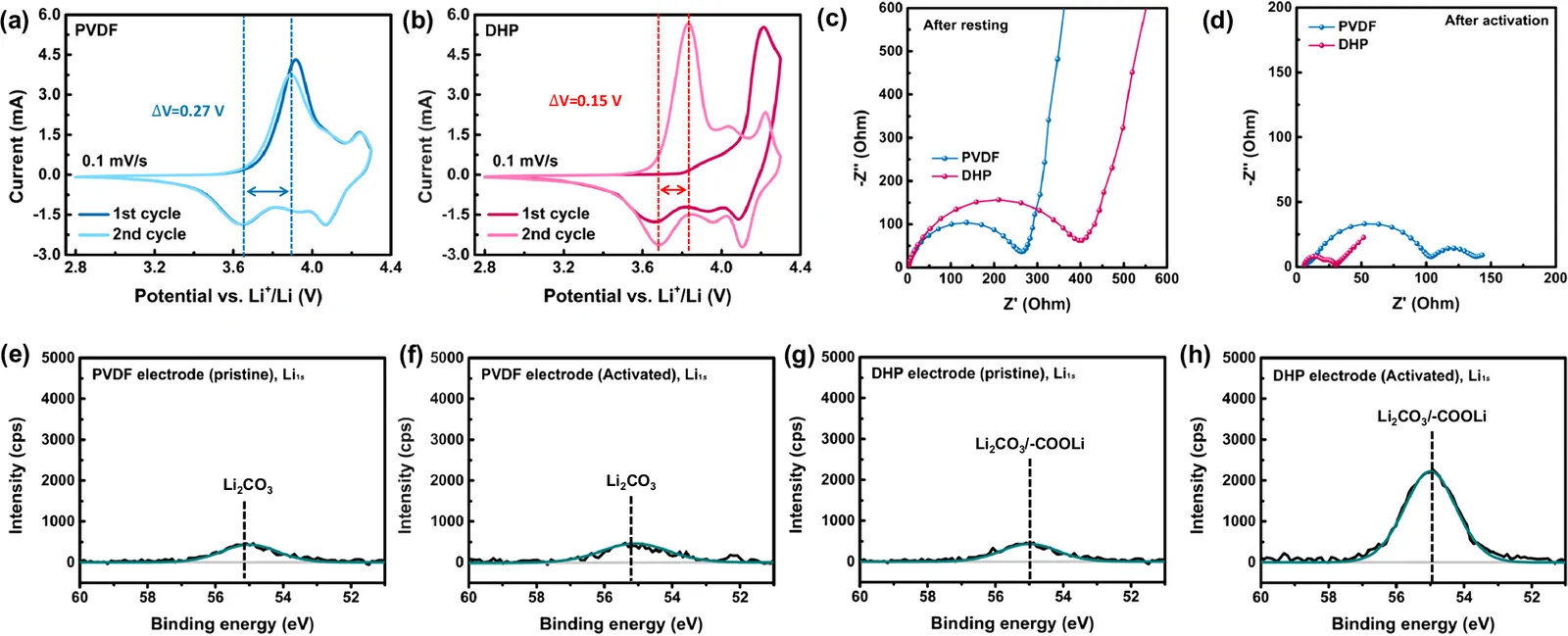
Analysis of the Li–PAA formation mechanism in NCM electrodes using the DHP binder. CV curves of the NCM electrodes using **a** PVDF and **b** DHP at the scan rate of 0.1 mV s^-1^. Nyquist impedance plots of the Ni-rich positive electrodes using PVDF and DHP after **c** 12 h resting and **d** 1st activation cycle in Li | |NCM cells cycled at 0.2 C (1 C = 185 mA g^-1^) within the voltage window of 2.8–4.3 V. Li 1 s XPS spectra of the Ni-rich positive electrodes using (**e**,**f**) PVDF and (**g**,**h**) DHP binder before and after activation cycle in Li | |NCM cells cycled at 0.2 C (1 C = 185 mA g^-1^) within the voltage window of 2.8–4.3 V.

As depicted in Fig. 2a, previous studies have established that the carboxyl groups of PAA undergo ion exchange with Li^+^ ions from the NCM, resulting in the formation of Li-PAA. The Li-PAA formation reaction and the associated change in interfacial resistance were verified by cyclic voltammetry (CV), electrochemical impedance spectroscopy (EIS), and X-ray photoelectron spectroscopy (XPS) analyses. As shown in Fig. 4a, characteristic peaks corresponding to the anodic peak of NCM 811 near 3.8 V were similarly observed in the first and second cycles of the CV results for the electrode using PVDF. By contrast, the CV analysis (Fig. 4b) revealed a sharp increase in current at nearly 4.0 V during the first cycle for DHP. This behavior is attributed to the H^+^/Li^+^ exchange reaction in PAA occurring at around 4.0 V, as previously reported in the literature, suggesting an in situ ion exchange reaction occurring in the NCM active material with DHP^40–42^. Notably, this sharp current increase disappeared in the second cycle, during which the DHP electrode exhibited a significantly narrower potential interval between redox peaks (ΔV = 0.15 V) compared to the PVDF electrode (ΔV = 0.27 V) (Fig. 4b), indicating reduced polarization during charge–discharge processes. These results suggest that the ion exchange between H⁺ and Li⁺ occurs within a specific voltage range, and the irreversible formation of Li-PAA during the initial charge process can reduce electrode polarization, thereby enhancing Li^+^ transport. The formation of Li-PAA on the NCM surface was further supported by EIS analysis. As shown in Fig. 4c, the initial resistance of the pristine DHP electrode was slightly higher than that of the PVDF electrode, which was attributed to the poor ionic conductivity of the DHP binder that uniformly covered the NCM surface prior to the formation of Li-PAA. Following the activation process, however, both the high-frequency CEI resistance (R_CEI_) and the lower-frequency charge-transfer resistance (R_CT_) decreased significantly (Fig. 4d), suggesting that the ionic conductivity of the DHP binder was enhanced by the in situ formation of a lithiophilic Li-PAA interface on the NCM surface^48^.

To further confirm the formation of Li-PAA, XPS analysis was performed on the PVDF and DHP electrodes before and after the first cycle. Figure 4e, f present the Li 1 *s* XPS profiles of PVDF before and after the first cycle, and Fig. 4g, h exhibit the Li 1 *s* peaks of DHP before and after the first cycle. A peak corresponding to Li_2_CO_3_ was observed for both the PVDF and DHP electrodes before cycling, likely due to the residual lithium-containing alkaline species remaining on the NCM surface after synthesis. After the activation cycle at 0.1 C, the Li 1 *s* peak at 55 eV in the PVDF electrode did not change significantly (Fig. 4e, f). By contrast, a notable increase in the Li 1 *s* peak was observed for the DHP electrodes after activation (Fig. 4g, h). This peak increase is mainly attributed to the formation of -COO^-^Li^+^ and not to Li_2_CO_3_, providing clear evidence of in situ Li-PAA formation during the activation process^40^. The Li 1 *s* peak at 55 eV in the DHP electrode increased after activation, indicating the formation of -COO^-^Li^+^ rather than Li_2_CO_3_. The post-cycling FT-IR analysis further supports the XPS results, as shown in Fig. S4. The characteristic –COOH peak of PAA observed in the pristine DHP electrode disappears after the first charge and discharge process, while a distinct –COOLi peak emerges at around 1550 cm^-1^. These results clearly demonstrate the formation of Li-PAA on the NCM surface during the first electrochemical cycle. In addition, the structural changes in NCM resulting from the activation process were investigated by the X-ray diffraction (XRD) analysis of the PVDF, SPDX, and DHP electrodes after activation. As shown in Fig. S5 (Supporting Information), no structural changes were observed in any of the electrodes compared to the pristine electrode, indicating that the ion-exchange step between the optimized DHP binder and NCM does not alter the crystal structure of NCM. Consequently, these experimental results confirmed the successful formation of Li-PAA on the NCM surface without compromising its structural integrity.

**Fig. 5:**
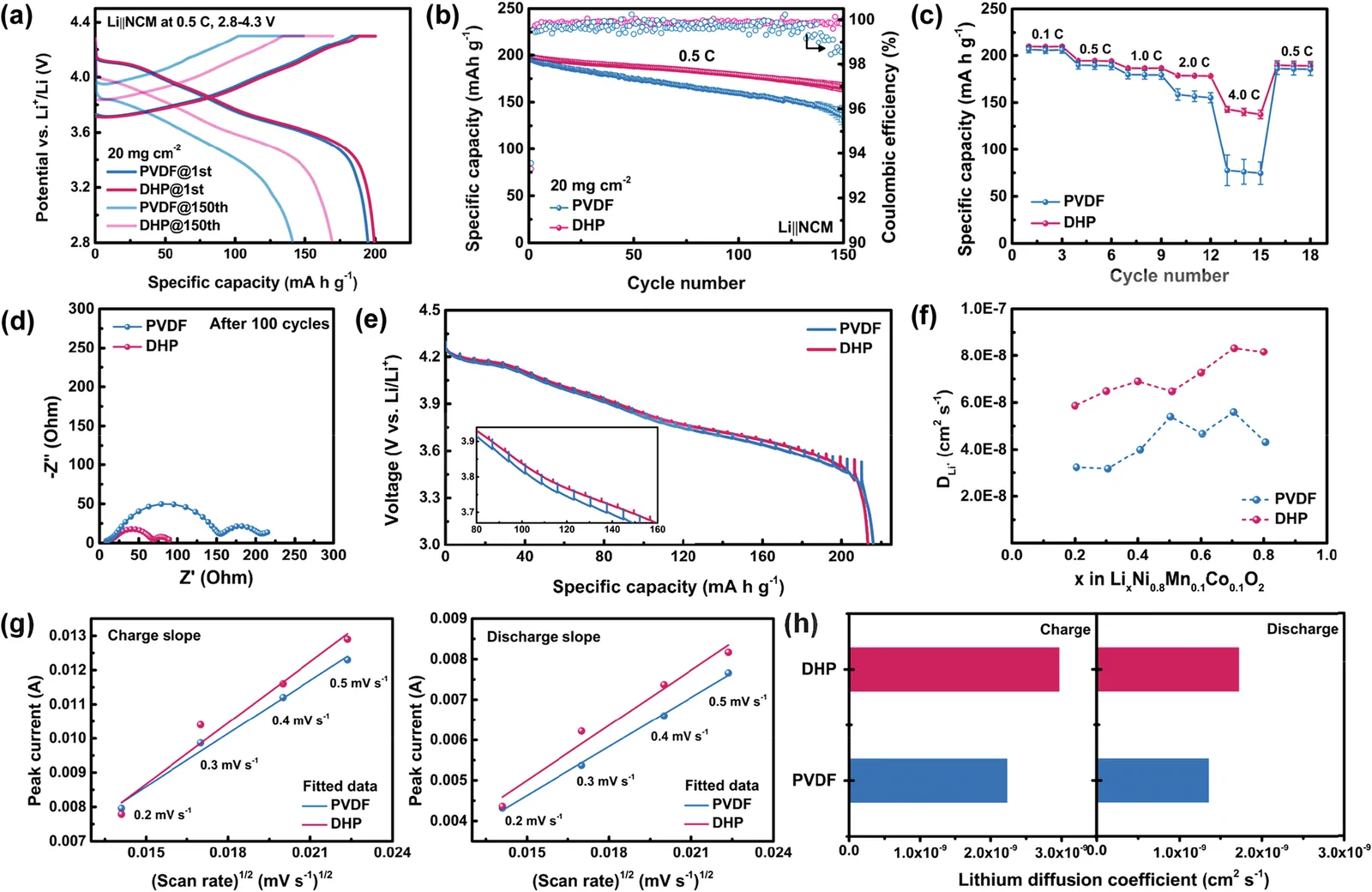
Electrochemical characterization of NCM electrodes. **a** Galvanostatic charge/discharge profiles of Ni-rich positive electrodes using PVDF and DHP in their first and 150th cycles at 0.5 C (1 C = 185 mA g^-1^). **b** Cycling performances of Ni-rich positive electrodes using PVDF and DHP, presented as discharge capacity, in the voltage window of 2.8–4.3 V when measured at 0.5 C, data are presented as mean ± standard deviation from three independent cells (*n* = 3). **c** Rate performance of both electrodes from 0.1 to 4 C in the voltage window of 2.8–4.3 V, data are presented as mean ± standard deviation from three independent cells (*n* = 3). **d** Nyquist plots of both electrodes after 100 cycles. **e** Transient Galvanostatic charge/discharge profiles obtained by GITT during the discharge process. **f** D_Li_^+^ of both electrodes as a function of the state of charge, which was estimated from the GITT. **g** Slopes of the redox peaks current of both electrodes from the CV results versus a square-root function of sweep rates. **h** D_Li_^+^ of both electrodes, which was estimated from the CV results.

To evaluate the efficacy of the DHP binder on the electrochemical performance, electrodes were prepared using an active material (NCM811), conductive agent (Super P), and binder (PVDF and DHP) in a weight ratio of 95:3:2. The resulting electrodes had a mass loading of 20.0 mg cm^-2^, which is the typical mass loading used in industry. Electrochemical tests were performed using coin-type Li | |NCM cells within a potential window of 2.8–4.3 V vs. Li/Li^+^. As shown in Fig. 5a, b, the cyclability of the positive electrodes was evaluated by conducting charge and discharge cycles at 0.5 C, following three initial formation cycles at 0.1 C (Fig. S6). After the formation process, the initial galvanostatic charge/discharge profiles indicated that the PVDF and DHP electrodes exhibited similar charge/discharge behaviors. However, after 150 cycles, the capacity retention of the PVDF electrode was measured to be 68.5%, whereas that of the DHP electrode was measured to be 83.5%, highlighting the significant difference in capacity retention between the PVDF and DHP binders. Additionally, the DHP electrodes exhibited improved rate performances compared to the PVDF electrode, as shown in Fig. 5c and Fig. S7 (Supporting Information). Across a range of C-rates from 0.1 to 4 C, the DHP electrodes consistently delivered higher discharge capacities than the PVDF electrode. Specifically, at 4 C, the DHP electrode delivered 142.8 mAh g^-1^, whereas the PVDF electrodes achieved 77.5 mAh g^-1^.

To comprehensively analyze the different electrochemical performances of the DHP and PVDF electrodes, EIS, galvanostatic intermittent titration technique (GITT), and cyclic voltammetry (CV) analyses were performed. As shown in Fig. 5d (EIS spectra after cycling), the Nyquist plots indicate that the DHP electrode exhibited a lower resistance of the CEI layer (R_CEI_) in the high-frequency region and lower resistance to charge transfer (R_CT_) in the lower-frequency region than the PVDF electrode^48^. The distinct difference between the semicircles is attributed to the formation of a highly Li^+^-conductive Li-PAA interface between the NCM and DHP binder, in contrast to the conventional PVDF electrode during cycling. To compare the electrode kinetics induced by Li-ion transport in the DHP and PVDF electrodes, GITT measurements were conducted at a fixed C-rate (0.1 C) with a sufficient relaxation period (30 min), enabling direct comparison of concentration polarization. In addition, the apparent Li^+^ diffusion coefficient (D_Li_^+^) was estimated using the following equation^49,50^:

1 $${D}_{{{Li}}^{+}}=\frac{4}{\pi }{\left(\frac{{R}_{S}}{3}\right)}^{2}{\left[\frac{\Delta {V}_{s}}{\Delta {V}_{t}}\right]}^{2}\left(\tau \ll \frac{{{R}_{s}}^{2}}{{D}_{S}}\right)$$

Figure 5e presents the GITT profiles of the PVDF and DHP electrodes measured at 30 °C. The DHP electrode exhibited a higher apparent Li-ion diffusion coefficient at the electrode level across the entire state-of-charge range than that of the PVDF electrode (Fig. 5f). This result indicates that the in situ-formed Li-PAA in the DHP electrode effectively reduces overall polarization, which is attributed to the accelerated lithium-ion diffusion within the electrode. As shown in Fig. 5g, h, the enhanced Li⁺ transport kinetics of DHP electrodes also exhibited similar trends in the CV curve measurements at various scan rates (0.2–0.5 mV s⁻¹). The results showed that both electrodes exhibited similar electrochemical reaction potentials, whereas the DHP electrode demonstrated a higher current response in all scan-rate ranges, as shown in Fig. S8 (Supporting Information). The D_Li_^+^ of the DHP electrode was found to be 2.972 × 10^-9^ and 1.728 × 10^-9^ cm^2^ s^-1^ in the charge/discharge stage, as calculated using the Randles–Sevcik equation (Fig. 5g, h)^51^:

2 $${i}_{p}=0.4463{\left(\frac{{n}^{3}{F}^{3}}{{RT}}\right)}^{\frac{1}{2}}A.C{(D.v)}^{1/2}$$

These values are higher than the D_Li_^+^ of the PVDF electrode calculated as 2.238 × 10^-9^ and 1.364 × 10^-9^ cm^2^ s^-1^, respectively (Fig. 5g, h). Accordingly, the CV and GITT results indicated that the DHP binder was more effective than the PVDF binder in improving the ion transport kinetics of the electrodes. Consequently, the enhanced electrochemical performance of the DHP electrodes can be attributed to the fact that the DHP binder not only facilitates uniform electrochemical behavior across the electrode by ensuring a homogeneous distribution of electrode components but also a more stable interface through the coverage effect of SPDX and the highly Li⁺-conductive Li-PAA.

### Postmortem analysis

**Fig. 6:**
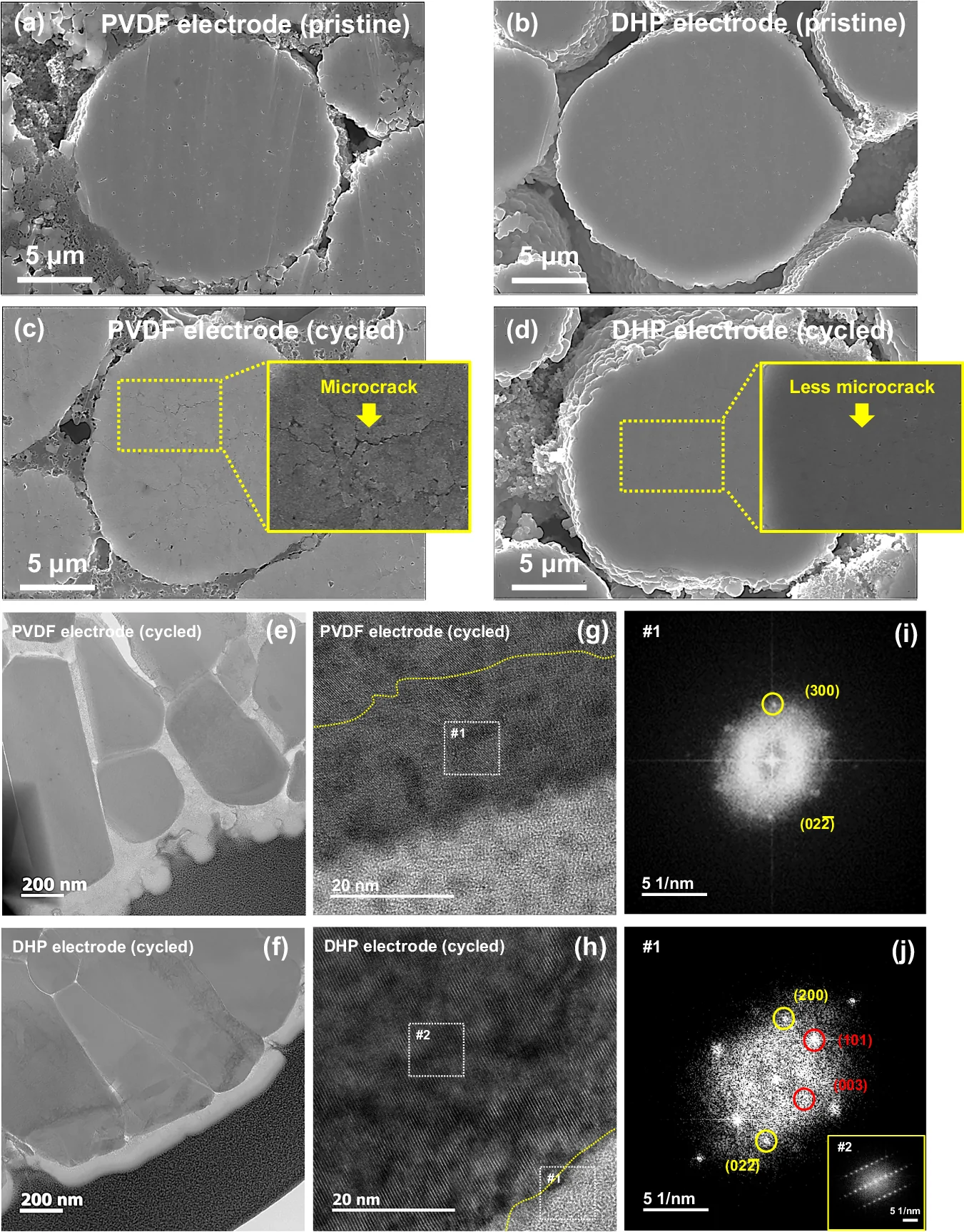
Post-mortem morphological analysis of NCM electrodes (20 mg cm^-2^) after 100 cycles in Li | |NCM cells cycled at 0.5 C (1 C = 185 mA g^-1^) under the voltage window of 2.8–4.3 V. Focused ion beam–scanning electron microscopy (FIB-SEM) images of the pristine Ni-rich positive electrodes using **a** PVDF and **b** DHP. FIB-SEM images of the cycled Ni-rich positive electrodes using **c** PVDF and **d** DHP. STEM images of the cycled NCM in **e** PVDF- and **f** DHP-based positive electrodes. HR-TEM images of the cycled NCM in **g** PVDF- and **h** DHP-based positive electrodes. Corresponding FFT patterns of the cycled NCM in **i** PVDF- and **j** DHP-based positive electrodes.

A postmortem analysis was conducted on the cycled DHP and PVDF electrodes to further confirm the protective effect of the DHP binder, which is composed of highly Li⁺-conductive Li-PAA and SPDX, against the structural degradation of the NCM positive materials. Cross-sectional SEM images were obtained using the focused ion beam (FIB) technique to assess the extent of microcracking in the cycled NCM811 particles within the PVDF and DHP electrodes. As shown in Fig. 6a, b, no microcracks were observed on the surface or bulk regions of the pristine NCM811 particles in either the PVDF or DHP electrode. However, after 100 cycles, clear morphological differences were observed between the two electrodes. Microcracks extending from the surface to the bulk region were observed on the PVDF electrode, whereas the DHP electrode exhibited significantly less structural damage (Fig. 6c, d). The well-maintained NCM particle morphology in the DHP electrode is attributed to the SPDX groups in DHP, which provide high elasticity and uniform binder coverage on the active materials, thereby mitigating the mechanical degradation of NCM. This was further supported by TEM analysis. As shown in the scanning TEM (STEM) images of the cross-sectioned NCM811 particles in the DHP electrode (Fig. 6f), the grain boundaries between the primary particles remained well preserved after cycling, without any signs of structural collapse. By contrast, the NCM811 particles in the cycled PVDF electrode (Fig. 6e) exhibited a structural collapse between the primary particles, accompanied by the formation of amorphous byproduct layers at the collapsed grain boundaries. This structural degradation of the PVDF electrode suggests that severe microcracks developed in the NCM during continuous cycling, facilitating electrolyte penetration and accelerating the degradation of the positive material^11,52,53^. These results demonstrate that the thin and uniform DHP coating layer formed on the surface of the positive material particles effectively suppresses structural degradation of NCM 811, including microcracks, caused by volume expansion during repeated lithiation and delithiation. To confirm the effect of DHP on the phase transitions of NCM811, high-resolution TEM (HR-TEM) and fast Fourier transform (FFT) analyses were conducted on positive electrodes cross sections after 100 cycles. As shown in Fig. 6g, in the case of the NCM in the PVDF electrode, the surface was evidently covered by a thick rock-salt structure (Fm̄3m) up to ~20 nm, which was confirmed by FFT analysis (Fig. 6i), indicating that this is due to the cation mixing in which Ni^2+^ having a similar ionic radius to Li^+^ moves to the existing Li site^11,54^. By contrast, the DHP electrode maintained a disordered spinel structure of ~5 nm and a complete rhombohedral layer structure (R̄3m) beyond this range (Fig. 6h, j), indicating that cation mixing was suppressed.

**Fig. 7:**
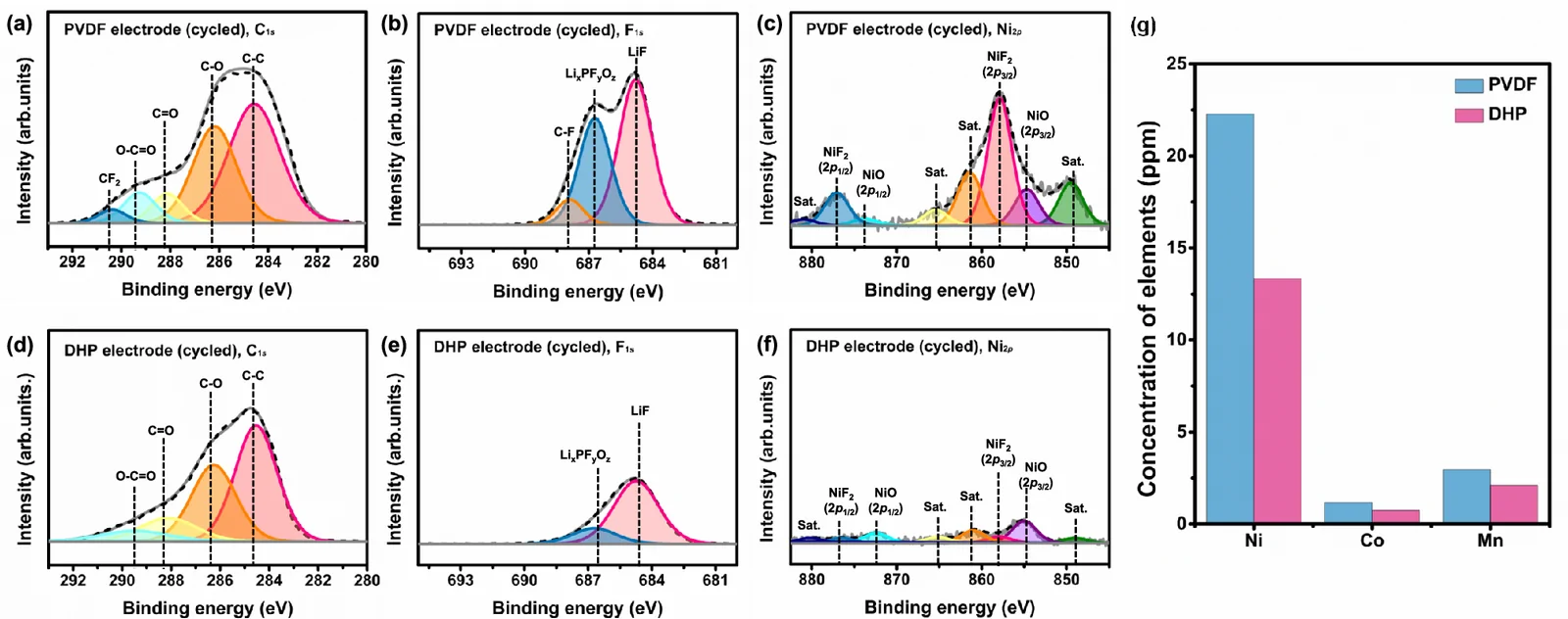
Post-mortem interfacial analysis of NCM electrodes (20 mg cm^-2^) after 100 cycles in Li | |NCM cells cycled at 0.5 C (1 C = 185 mA g^-1^) under the voltage window of 2.8–4.3 V. C 1 *s* XPS spectra of the cycled Ni-rich positive electrodes using **a** PVDF and **d** DHP. F 1 *s* XPS spectra of the cycled Ni-rich positive electrodes using **b** PVDF and **e** DHP. Ni 2*p* XPS spectra of the cycled Ni-rich positive electrodes using **c** PVDF and **f** DHP. For the XPS spectra, the black dotted lines, gray solid lines, and colored peaks represent the raw data, fitted curves, and deconvoluted components, respectively. **g** Concentration of metallic Ni, Co, and Mn ions deposited on the surface of Li metal electrode of the PVDF and DHP cell determined by ICP-MS.

To further confirm the inhibitory effect of DHP on the surface degradation of the NCM positive electrodes, XPS analysis was conducted on the surface of the NCM electrodes after cycling, as shown in Fig. 7a–f and S9. In the C 1 s spectra, both the PVDF and DHP electrodes showed a C-O peak at 286.3 eV, associated with the decomposition of the carbonate-based electrolyte, with the PVDF electrodes displaying a notably higher peak intensity (Fig. 7a, d). In the F 1 s spectra (Fig. 7b, e), two peaks corresponding to LiF and Li_x_PF_y_O_z_, major decomposition products of the electrolyte on the NCM surface, were observed at 685.5 and 686.7 eV, respectively. The DHP electrodes (Fig. 7e) exhibited significantly reduced peak intensities for both species compared to the PVDF electrodes (Fig. 7b), indicating the effective inhibition of electrolyte decomposition and suppression of unstable CEI formation owing to the surface coverage provided by the DHP binder^55^. Further evidence of structural stabilization by the DHP binder was observed in the Ni 2*p* spectra. For the cycled PVDF electrode, a prominent NiO peak at 854.8 eV and a NiF_2_ peak at 877.1 eV were observed for the cycled PVDF electrode. The pronounced formation of NiF_2_ indicates significant degradation of the NCM positive electrodes, resulting from transition metal dissolution and electrolyte-induced side reactions (Fig. 7c)^55,56^. By contrast, the cycled DHP electrodes exhibited significantly lower intensities for the major peaks owing to the effective coverage of the binder, and NiF_2_ peak was hardly detected (Fig. 7f). To complement these findings, inductively coupled plasma–mass spectrometry (ICP-MS) analysis was performed to investigate the effect of DHP on transition metal (TM) ion chelation during cycling. As shown in Fig. 7g, the DHP electrode exhibited lower concentrations of Ni, Co, and Mn on the Li metal surface than the PVDF electrode, confirming the suppression of TM dissolution through chelation by the enriched carboxyl groups from the PAA component in the DHP binder^57,58^. Based on these structural and compositional postmortem analyses, we suggest that the DHP binder prevents direct contact between the electrolyte and the highly reactive positive material surface, thereby suppressing the formation of the impedance-increasing disordered rock-salt phase and transition metal (TM) dissolution. Moreover, it mitigates the accumulation of surface residues during cycling, which ultimately contributes to the structural stabilization of NCM.

### Evaluation of high-mass-loading electrodes with DHP

The previous section confirmed that the DHP binder improves the electrode structural integrity and Li^+^ transport kinetics while suppressing the degradation of Ni-rich positive electrodes. Based on these findings, the feasibility of the DHP binder for high-mass-loading electrodes was further investigated to enhance the areal capacity of the positive electrodes and the energy density of the battery. NCM811 positive electrodes with DHP were fabricated with areal loading levels ranging from 20 to 70 mg cm^-2^, corresponding to 130–260 μm thicknesses. For comparison with the high-mass-loading DHP electrode, electrodes based on PAA, PVDF, and SPDX binders were also prepared. However, the NCM811 positive electrode with PAA exhibited poor mechanical properties owing to the intrinsic rigidity of PAA, which makes it difficult to fabricate high-mass-loading electrodes. Even at a relatively low loading level of 20 mg cm^-2^, the PAA-based electrode exhibited nonuniform and weak adhesion to the current collector (Fig. S3). By contrast, at a high-mass-loading of 45 mg cm^-2^, both the SPDX and DHP electrodes displayed clean surface morphologies and favorable rheological behaviors, while the PVDF electrode showed severe cracking (Fig. S10). Notably, the DHP electrode maintained a stable and intact surface morphology even at a high-mass loading of 70 mg cm^-2^, whereas the PVDF electrode exhibited severe cracking across more than half of its surface area (Fig. S11).

**Fig. 8:**
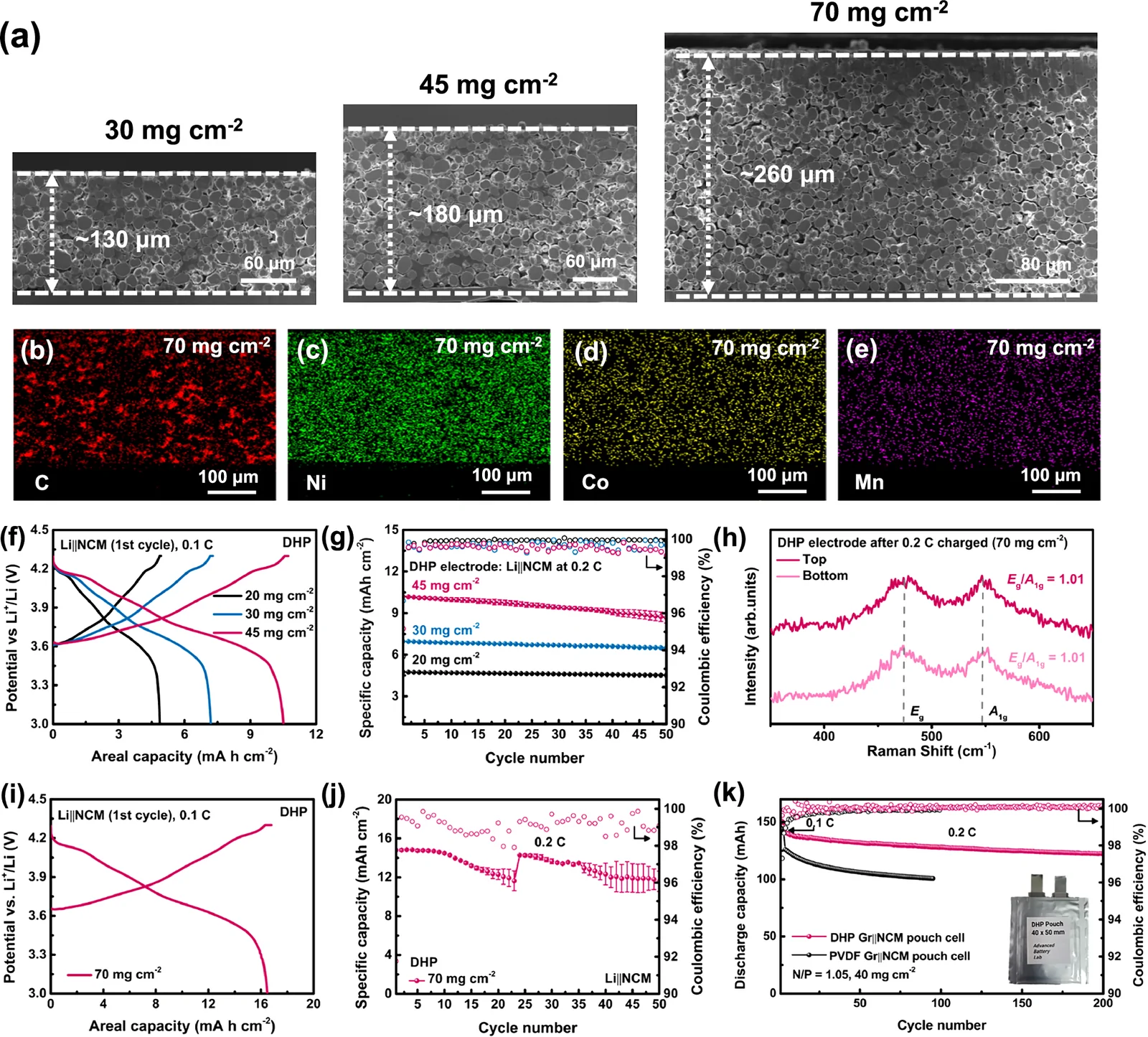
Electrochemical and structural characterization of high-mass-loading NCM electrodes using the DHP binder. **a** Cross-sectional SEM images of the DHP electrodes with different areal mass loadings. Energy dispersive X-ray spectroscopy (EDS) images of the high areal mass-loading DHP electrodes (70 mg cm^-2^); **b** carbon, **c** nickel, **d** cobalt, **e** manganese. **f** Galvanostatic charge/discharge profiles of the cells (Li | |DHP electrode coin cell) as a function of the areal capacity at 0.1 C (1 C = 185 mA g^-1^). **g** Cycling performance of the DHP electrodes with different areal mass loadings, presented as discharge capacity, in the voltage window of 2.8–4.3 V measured at 0.2 C, data are presented as mean ± standard deviation from three independent cells (*n* = 3). **h** Raman spectra and the corresponding *E*_g_/*A*_1g_ intensity ratio obtained from the top and bottom regions of the DHP positive electrodes with 70 mg cm^-2^, after being charged to 4.3 V (100% SOC) at 0.2 C-rate. **i** Galvanostatic charge/discharge profiles of the cells (Li | |70 mg cm^-2^ DHP electrode coin cell) as a function of the areal capacity at 0.1 C. **j** Cycling performances of electrodes with high areal mass loadings, presented as discharge capacity, in the voltage window of 2.8–4.3 V measured at 0.2 C, data are presented as mean ± standard deviation from three independent cells (*n* = 3). k Cycling performance of the Graphite | |NCM pouch cells using DHP- and PVDF-based positive electrodes (40 mg cm^-2^) in the voltage window of 2.7–4.2 V measured at 0.2 C.

The robust and highly adhesive structural characteristics of the DHP electrode were validated through cross-sectional SEM images of electrodes with mass loadings of 30, 45, and 70 mg cm^-2^. As shown in Fig. 8a, the high-mass-loading DHP electrode exhibited strong cohesion and adhesion between the electrode components. Notably, at a high mass loading of 70 mg cm^-2^ (thickness: 260 μm), a uniform distribution between the electrode materials was clearly observed, which was confirmed by the EDS analysis (Fig. 8b–e). These results demonstrate that the previously described rheological properties and functional advantages of the DHP binder, such as its ability to construct a robust, well-dispersed electrode structure and promote strong interfacial adhesion, remain effective even for high-mass-loading electrodes.

Electrochemical testing of the high-mass-loading electrodes was performed in galvanostatic mode using coin-type Li | |NCM cells. The PVDF-, SPDX-, and DHP-based electrodes with mass loadings of 30 mg cm^-2^ were fabricated, and their electrochemical performances were evaluated. As shown in Fig. S12, the DHP-based electrode exhibited a stable cycling performance up to 50 cycles, whereas the PVDF- and SPDX-based electrodes exhibited noticeable capacity fading after 30 cycles. Interestingly, although high-mass-loading electrodes were successfully fabricated with the SPDX binder, the ion transport issues in the thick electrodes were not resolved by the SPDX binder alone, leading to rapid performance degradation. Therefore, the SPDX-based electrode exhibited a sharp increase in polarization and lower cyclability than the DHP-based electrode even at a mass loading of 20 mg cm^-2^ (Fig. S13), and severe polarization in the Galvanostatic charge/discharge profile from the first cycle at 45 mg cm^-2^ was observed (Fig. S14). These findings clearly demonstrate that the SPDX binder cannot effectively mitigate the ion transport issues in thick electrodes, making it unsuitable for high-mass-load electrodes. In sharp contrast, the DHP electrodes demonstrated low polarization in the Galvanostatic charge/discharge profile during formation cycle and stable cycling performance up to 50 cycles at a high mass loading of 45 mg cm^-2^ (Fig. 8f, g). More notably, DHP-based electrodes exhibited stable electrochemical performance even at high mass loading of 70 mg cm^-2^. Before the cycling test, the redox uniformity of the DHP-based electrode at a high mass loading of 70 mg cm^-2^ was investigated. The DHP-based positive electrode was charged to 4.3 V at a 0.2 C-rate, and Raman measurements were conducted at both the top and bottom regions of the electrode, based on their distance from the current collector, to determine the local state of charge (SOC) of the active material. After charging, the DHP-based electrode exhibited two distinct Raman peaks corresponding to the *E*_g_ (475 cm^-1^) and A_1g_ (550 cm^-1^) modes, with an intensity ratio (*E*_g_/*A*_1g_) of 1.01 (Fig. 8h). Since the *E*_g_/*A*_1g_ intensity ratio is known to increase proportionally with the state of charge (SOC) and reflects the degree of delithiation in NCM, the consistency of this ratio at different regions of the electrode confirms the uniformity of the local SOC^59,60^. Accordingly, the Raman results indicate uniform redox behavior throughout the DHP-based positive electrodes, which is driven by the facile Li^+^ transport in the electrode. Attributed to the uniform electrochemical reactions, the DHP-based electrode with a mass loading of 70 mg cm^-2^ delivered an areal capacity of 16.6 mA h cm^-2^ and maintained a stable cycling performance for up to 25 cycles (Fig. 8i, j). After 25 cycles, to eliminate the influence of Li metal negative electrode degradation, which is a typical issue when pairing a high-mass-loading positive electrodes with a Li metal negative electrode, all cell components except the high-mass-loading positive electrodes were replaced, and the cycling tests were subsequently continued. The initial areal capacity was recovered, and stable cycling performance was maintained for up to 50 cycles, indicating that the observed capacity fading after 25 cycles originated from the Li metal electrode degradation rather than the high-mass-loading positive electrodes. Moreover, as shown in Table S1, the estimated specific energy of a 10 Ah-class pouch cell employing a DHP-based electrode with a mass loading of 70 mg cm^-2^ reached 474 Wh kg^-1^, which is over 20% higher than that of the 25LL electrode (391 Wh kg^-1^) (Calculation details are provided in Table S1).

For ultimately evaluating industrial applicability of the DHP binder, Graphite | |NCM pouch cells were fabricated using DHP- and PVDF-based positive electrodes (40 mg cm^-2^), and the cells were subjected to three formation cycles at 0.1 C, followed by continuous cycling at 0.2 C (Fig. 8k). The pouch cell incorporating a DHP-based electrode exhibited a higher capacity of 149.2 mAh in the first formation cycle compared to PVDF-based cell (144.9 mAh), despite identical areal loading and N/P ratio conditions. Moreover, in the 0.2 C cycling test, the DHP-based pouch cell exhibited both higher capacity and stable cycling performance, retaining 86.8% of its initial capacity after 200 cycles. In sharp contrast, the PVDF-based cell reached its end of life at 95 cycles, with end of life defined as the point at which the capacity drops to 80% of its initial value. These results collectively indicate that the DHP binder serves as a viable strategy to enhance the energy density and electrochemical performance of LIBs for practical applications, beyond the laboratory level. These comprehensive results demonstrate that the DHP binder is well suited for fabricating high-thickness electrodes. The high-mass-loading electrode employing the DHP binder exhibited a competitive performance, achieving minimal binder content, high mass-loading levels, and high areal capacity compared to previously reported binders for NCM electrodes (Table S2).

## Discussion

In an effort to advance the technological maturity of binders for thick electrodes, particularly for achieving a high-mass-loading, thick positive electrodes using a conventional wet process, a DHP binder was investigated. This binder was successfully designed by the simple crosslinking of SPDX and PAA, which were carefully selected to address the fundamental challenges of thick Ni-rich positive electrodes, namely, poor structural stability and limited ionic mobility. Highly elastic SPDX offers a well-dispersed electrode structure and favorable cohesion and adhesion properties, whereas PAA facilitates Li^+^ transport through the in situ formation of Li–PAA. Benefiting from the SPDX component, the DHP binder provided thinner and more uniform coverage on the surface of the NCM811 electrode than the conventional PVDF binder, even after electrode fabrication with the same binder content. These characteristics of the DHP binder contributed to the stabilization of the electrode structure by enhancing the cohesion and adhesion strength, while also suppressing TM dissolution and cation mixing within the positive material particles during cycling. In addition, Li-PAA formed after the formation process enhanced the uniformity of the electrochemical reactions within the high-mass-loaded electrode. Therefore, the DHP binder enabled the fabrication of high-mass-loading positive electrode with stable cycling and improved rate performance. Moreover, owing to its favorable properties, the DHP binder enabled the successful fabrication of crack-free, well-dispersed high-mass-loading electrodes (70 mg cm^-2^) with a binder content of only 2 wt.% while minimizing the electrochemical performance degradation. Such a high loading level is considered unattainable with conventional single-component binders, such as PAA, PVDF, or SPDX. The promising electrochemical performance of the DHP-based positive electrode was further demonstrated by the implementation of a pouch cell with high-loading electrode, underscoring its viability for practical applications. Consequently, we believe that the binder development strategy proposed in this study represents a promising approach in overcoming the limitations of Ni-rich positive materials and enhancing the feasibility of realizing high-energy-density LIBs.

## Methods

### Synthesis of multifunctional crosslinked polymers

SPDX (TK Chemical, Mw ≈ 213 000, Republic of Korea) and PAA (Sigma Aldrich, Mw ≈ 450 000, USA) powders were added to a vial, and a certain amount of N-Methyl-2-Pyrrolidone (Junsei Chemical, 99.0%, Japan) was added. Mechanical stirring was conducted at 60 °C for 12 h to obtain the final DHP binder. The optimal ratio of the two polymers in the DHP binder was determined through electrochemical and peel tests conducted on NCM positive electrodes using binders with various weight ratios of SPDX to PAA (2:1, 1:1, 4:1, and 1:2). Among these, a 2:1 ratio was identified as the most effective because it exhibited a stable electrode morphology and favorable electrochemical and mechanical properties (Fig. S15).

### Preparation of electrodes and cells

DHP-based electrodes were prepared by dispersing LiNi_0.8_Co_0.1_Mn_0.1_ (NCM811, POSCO Future M, D50 = 10 μm, Republic of Korea), Super P (Timcal, Switzerland), and the DHP binder in NMP (Junsei Chemical, 99.0%, Japan) at a weight ratio of 95:3:2. The resulting slurry was applied to an aluminum foil current collector (15 μm) using the doctor blade technique with automatic coating machine (CT-AF300V, CORETECH, Republic of Korea), dried under vacuum at 80 °C for 6 h, roll-pressed to a composite density of 3.3–3.4 g cm^-3^, and then dried overnight at 100 °C. Control electrodes using PVDF (Sigma Aldrich, Mw ≈ 534,000), PAA, and SPDX binders were fabricated similarly. The active material loadings were set to 20, 30, 45, and 70 mg cm^-2^ for both DHP- and PVDF-based samples, corresponding to doctor blade gaps of 220, 350 μm, 600 μm, and 850 μm, respectively. Graphite electrodes were fabricated by slurry casting method. The slurry was prepared by dispersing graphite powder (BTR, S360-L2-H), carboxymethylcellulose (CMC, Sigma Aldrich, Mw ≈ 250 000), and Polymerized Styrene Butadiene Rubber (SBR, MTI Korea, Mw ≈ 534,000) binder at a ratio of 97:1.5:1.5 in deionized water. The prepared slurry was cast onto Cu foil (11 μm, Lotte Energy Materials, Republic of Korea). The obtained film was dried under vacuum at 100 °C for 6 h, roll-pressed to a composite density of 1.5 g cm^-3^, and then dried overnight at 100 °C. Coin-type CR2032 cells (Stainless steel (SUS) cases and springs) using a single-side-coated NCM positive electrode (12 mm in diameter) and lithium metal (200 μm, 14 mm in diameter, Honjo Metal) negative electrode were assembled in an argon-filled glovebox with H_2_O and O_2_ levels maintained below 0.1 ppm. Lithium metal was stored and handled in an argon-filled glovebox with H_2_O and O_2_ levels maintained below 0.1 ppm, and was used within 2 months after opening. A polyethylene (PE) separator (18 mm in diameter, W-scope, WL 16 C, 16 μm thickness, 45.0 ± 5.0% porosity, and an average pore size of 0.21 μm) and an electrolyte consisting of 1 M LiPF_6_ in EC/DEC (3:7 v/v) with 10 wt% FEC (PANAX ETEC) were used, and 60 μL of electrolyte was added to each cell. For the preparation of Gr | |NCM pouch full cells, the NCM positive electrode was cut to dimensions of 40 × 50 mm, and the Gr negative electrode was cut to 45 × 55 mm. The E/C and N/P ratio of pouch cells were set to 2.0 g Ah^-1^ and 1.05, respectively. Pouch cells were assembled in dry room with a dew point of −50 °C.

### Characterization

Rheological measurements were conducted using an Advanced Rheometric Expansion System (ARES-G2, TA Instruments, USA) using 6 wt% binder solutions, with steady shear sweep measurements performed over a shear rate range of 0.1–300 s^-1^. The samples were sealed under an Ar atmosphere during transport and transferred immediately before the measurements to minimize air exposure. Mechanical characteristics of the electrodes were assessed using a universal testing machine (UTM, JSV-H1000), including 180° peel tests using 3 M double-sided tape at a peel rate of 25 mm min^-1^. All samples used for the peel tests were prepared with a fixed width of 1 cm, and peeling strength normalized by sample width (N m^-1^). FT-IR spectroscopy (TEMSOR27, Bruker, Germany) was employed to investigate the polymeric bonding structures. Electrode morphology was observed through FE-SEM (SU8010, HITACHI) and cross-sectional imaging via FIB (Helios 650, FEI, USA). Crystalline features of the active materials were analyzed using XRD (Empyrean, PANalytical, Cu-Kα source). Transition metal dissolution was studied via EDS mapping (JSM-7800F Prime, JEOL, Japan) and quantified using ICP-MS (NexION 350D, Perkin-Elmer, USA), with samples preconditioned in fresh electrolyte at 60 °C. XPS (ESCALAB-250, Thermo Scientific, Al Kα) was utilized for surface composition analysis, and the binding energies of all XPS spectra were calibrated by referencing the C-C bond peak corresponding to C1s to 284.8 eV. HR-TEM (JEOL ARM-200F) provided atomic-resolution imaging at 200 kV. The XPS spectra were processed using Shirley background subtraction and fitted using Gaussian–Lorentzian functions. Raman spectroscopy (LabRamn Soleil, Horiba) analysis was conducted using a 532 nm laser over a wavenumber range of 350–650 cm^−1^ with a resolution of 2 cm^−1^. For post-mortem analyses (XRD, XPS, SEM, TEM, and Raman), the NCM electrode was collected from the disassembled cycled coin cell, washed with DEC, and fully dried in an argon-filled glovebox with H_2_O and O_2_ levels maintained below 0.1 ppm at 25 ± 3 °C. The harvested samples were sealed under an Ar atmosphere during transport and transferred immediately before the measurements to minimize air exposure.

### Electrochemical measurements

Cells were rested for 12 h before testing to ensure complete electrolyte infiltration. Formation cycles (first three cycles) were performed at 0.1 C to stabilize the cathode–electrolyte interphase (CEI). Galvanostatic charge/discharge tests were conducted in a potential window of 2.8–4.3 V vs. Li/Li^+^ using a battery cycler (WBCS 3000, WonAtech, South Korea) at 30 °C ± 1 °C in an environmental chamber (Hanbaek Scientific Technology, South Korea). Cyclability tests followed at 0.5 C, while rate capability was assessed by varying the current rate from 0.1 C to 4 C. For reproducibility, all coin cell tests were conducted in triplicate, and the results are presented as the mean values with standard deviations (SD). The cycling performance of the Graphite | |DHP electrode pouch full cells was evaluated in a voltage window of 2.7–4.2 V at 0.2 C, without applying a controlled external stack pressure during testing. The electrolyte composition and operating temperature used for the pouch cell evaluation were identical to those used in the coin cell evaluation. GITT measurements were conducted at a discharge rate of 0.1 C with a relaxation time of 30 min for each step. Voltage data during the GITT measurements were recorded at 2 points s^-1^. Electrochemical stability of the polymer binders was evaluated through CV at a scan rate of 0.1 mV s^-1^ over the same potential range using a potentiostat (VSP, Bio-Logic, France). EIS measurements were conducted in potentiostatic mode using a potentiostat with a 10 mV amplitude over a frequency range from 10 to 1 MHz with ten points per decade under open-circuit voltage conditions.

## Supplementary information

Supplementary Information

Transparent Peer Review file

## Source data

Source Data

## Data Availability

The data generated in this study are provided in the Source Data files. Source data are provided with this paper.
