# Supplementary material for: Wet-processed high-areal-capacity electrodes via transformative spandex–poly(acrylic acid) binder toward 450 Wh kg−1 lithium-ion batteries
Source: Nat Commun. 2026 Jul 23;17:8986. doi: 10.1038/s41467-026-76014-4 (PMC13507357; doi:10.1038/s41467-026-76014-4)
Supplement: Supplementary file 1 [file 41467_2026_76014_MOESM1_ESM.pdf]

Supporting Information for

Wet-processed high-areal-capacity electrodes via transformative spandex–poly(acrylic acid) binder toward 450 Wh kg^{-1} lithium-ion batteries

Bo Keun Park[1‡], Yoon Bo Shim[1‡], Jong Uk Won[1], Sung Joon Park[1], Yeon Jeong Kim[2], Byeongjin Park[2], Jang Wook Choi[3*], Ki Jae Kim[1,2,4*]

[1] Department of Energy Science, Sungkyunkwan University, 2066, Seobu-ro, Jangan-gu, Suwon-si, Gyeonggi-do, 16419, Republic of Korea

[2] Department of Future Energy Engineering, Sungkyunkwan University, 2066, Seobu-ro, Jangan-gu, Suwon-si, Gyeonggi-do, 16419, Republic of Korea

[3] School of Chemical and Biological Engineering and Institute of Chemical Process, Seoul National University, Gwanak-ro, Gwanak-gu, Seoul 08826, Republic of Korea

[4] SKKU Institute of Energy Science and Technology (SIEST), Sungkyunkwan University, 2066, Seobu-ro, Jangan-gu, Suwon-si, Gyeonggi-do, 16419, Republic of Korea

[‡] These authors equally contributed to this work.

[*]Corresponding authors: E-mail: jangwookchoi@snu.ac.kr, kijaekim@skku.edu

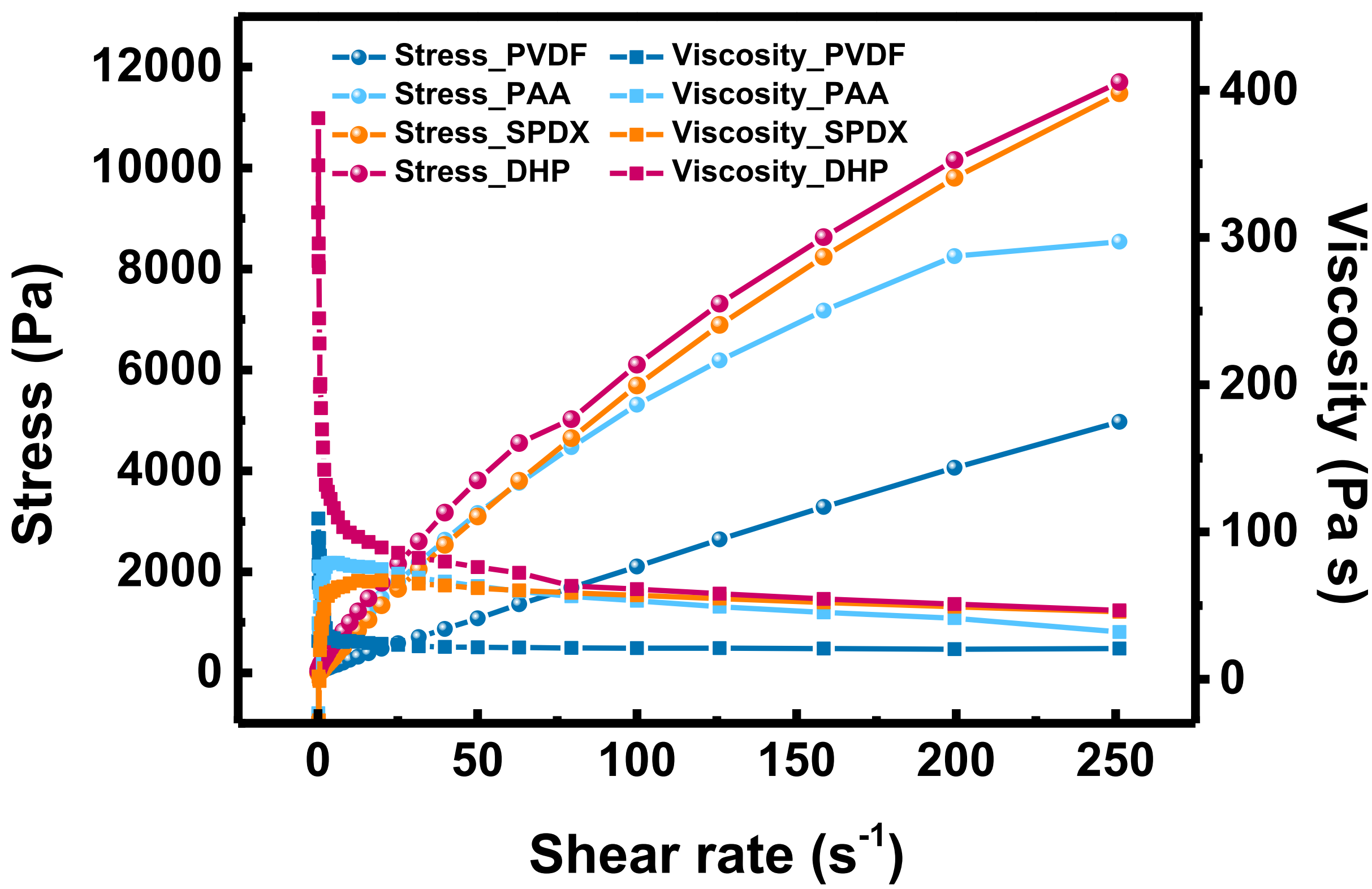

Fig. S1. Rheological data of the PVDF, PAA, SPDX, and DHP electrode slurries prepared using the same process for the active material, conductive agent, and binder.

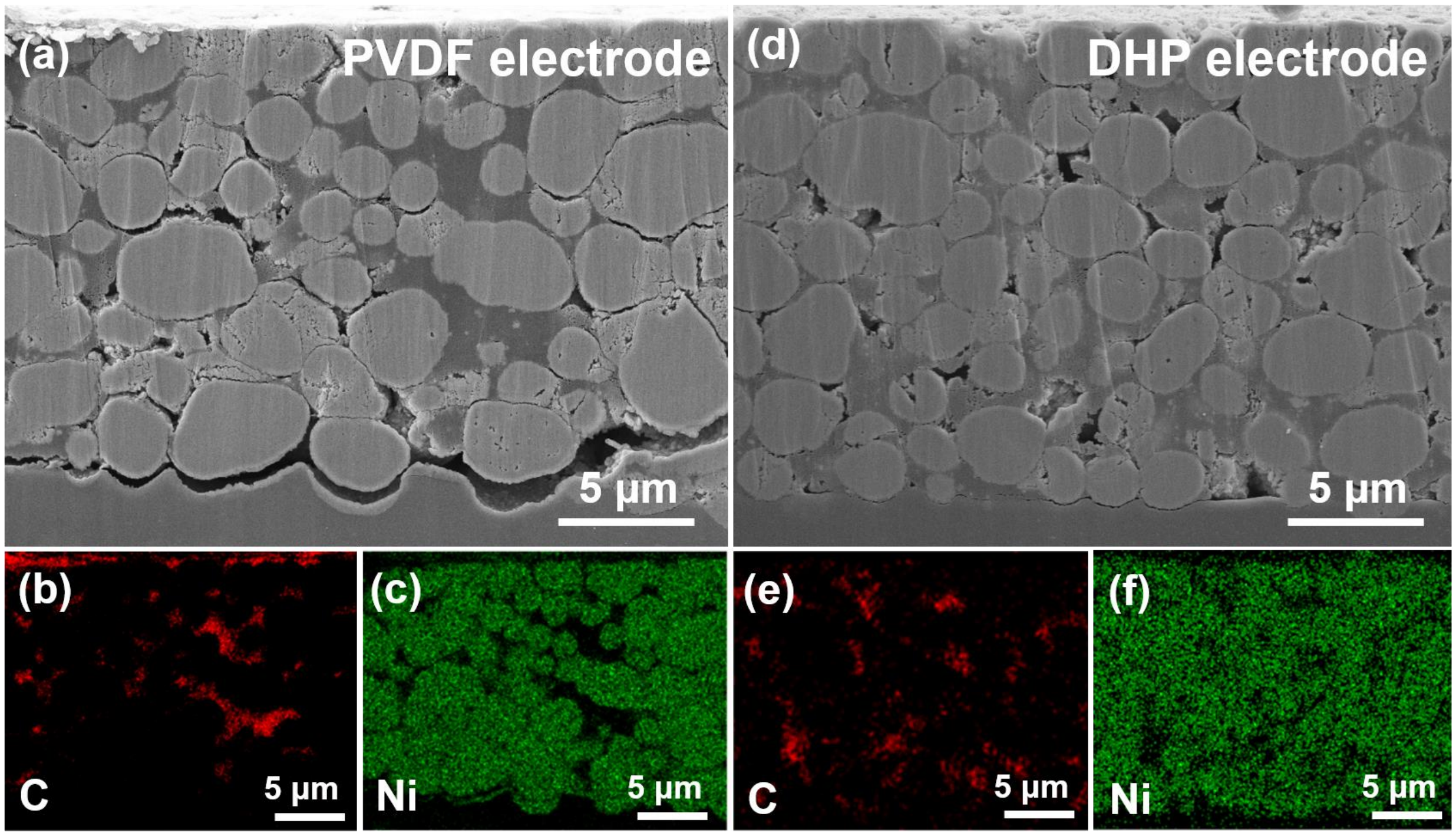

Fig. S2. Cross-sectional SEM images of the NCM electrode prepared with (a) PVDF and (d) DHP binders and the corresponding EDS maps: (b), (e) C (in red) and (c), (f) Ni (in green).

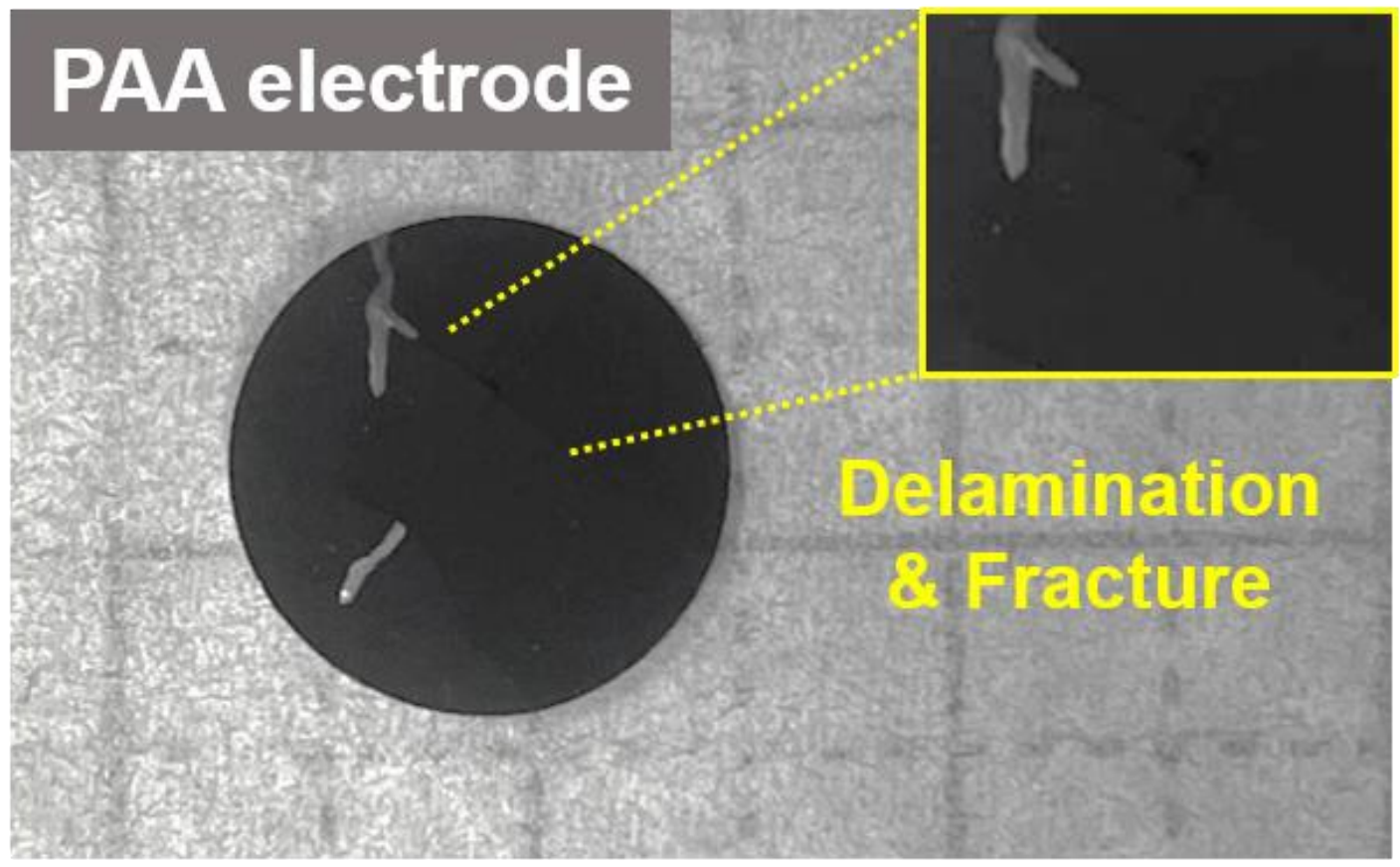

Fig. S3. Photograph of the PAA electrode at 20 mg cm^{-2} (NCM811/Super P/binder = 95/3/2 (w/w/w)).

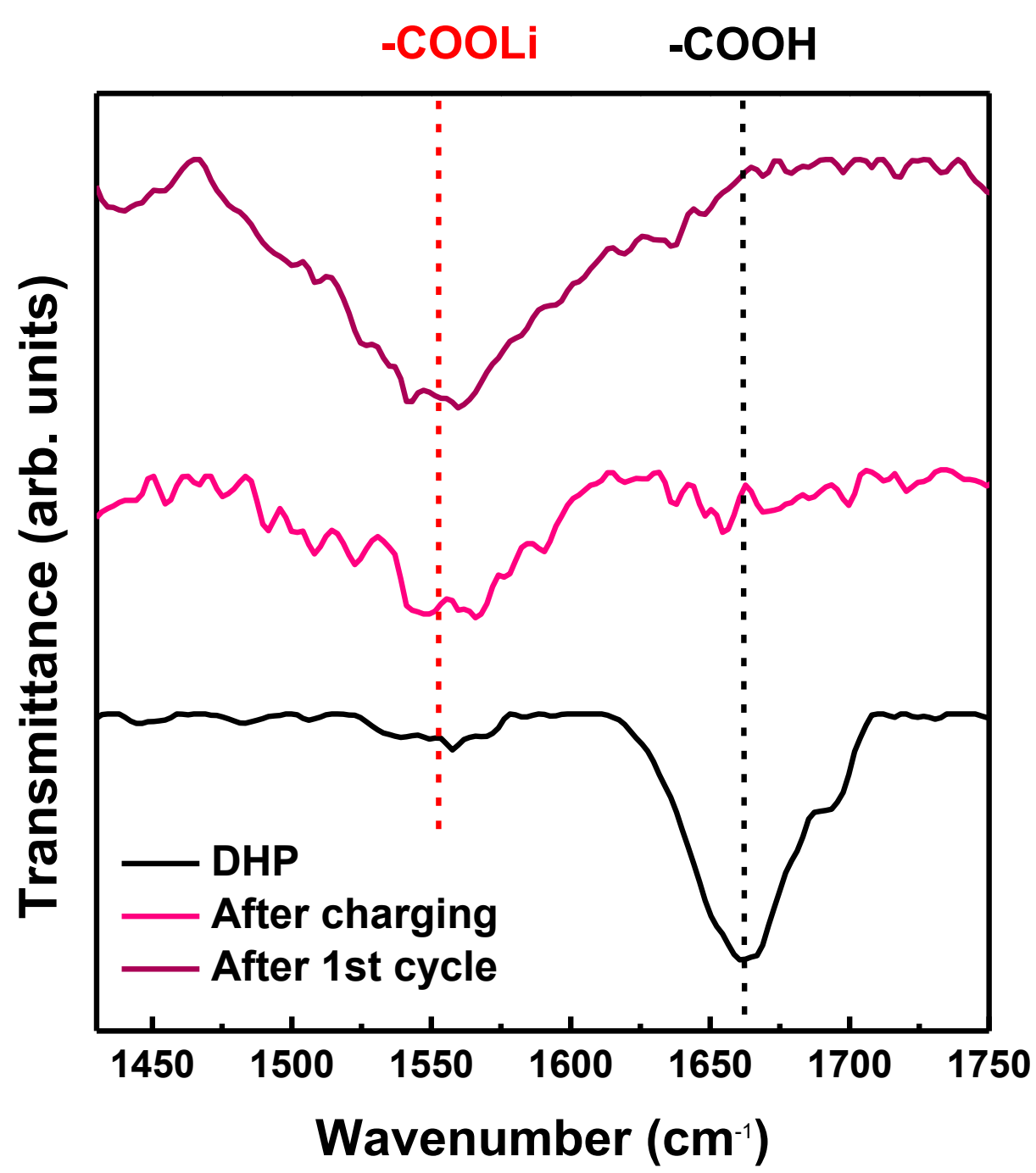

Fig. S4. FT-IR spectra of the pristine DHP electrode and cycled DHP electrodes (20 mg cm^{-2}) after charging and the first cycle in Li||NCM cells cycled at 0.2 C (1 C = 185 mA g^{-1}) in the voltage window of 2.8–4.3 V.

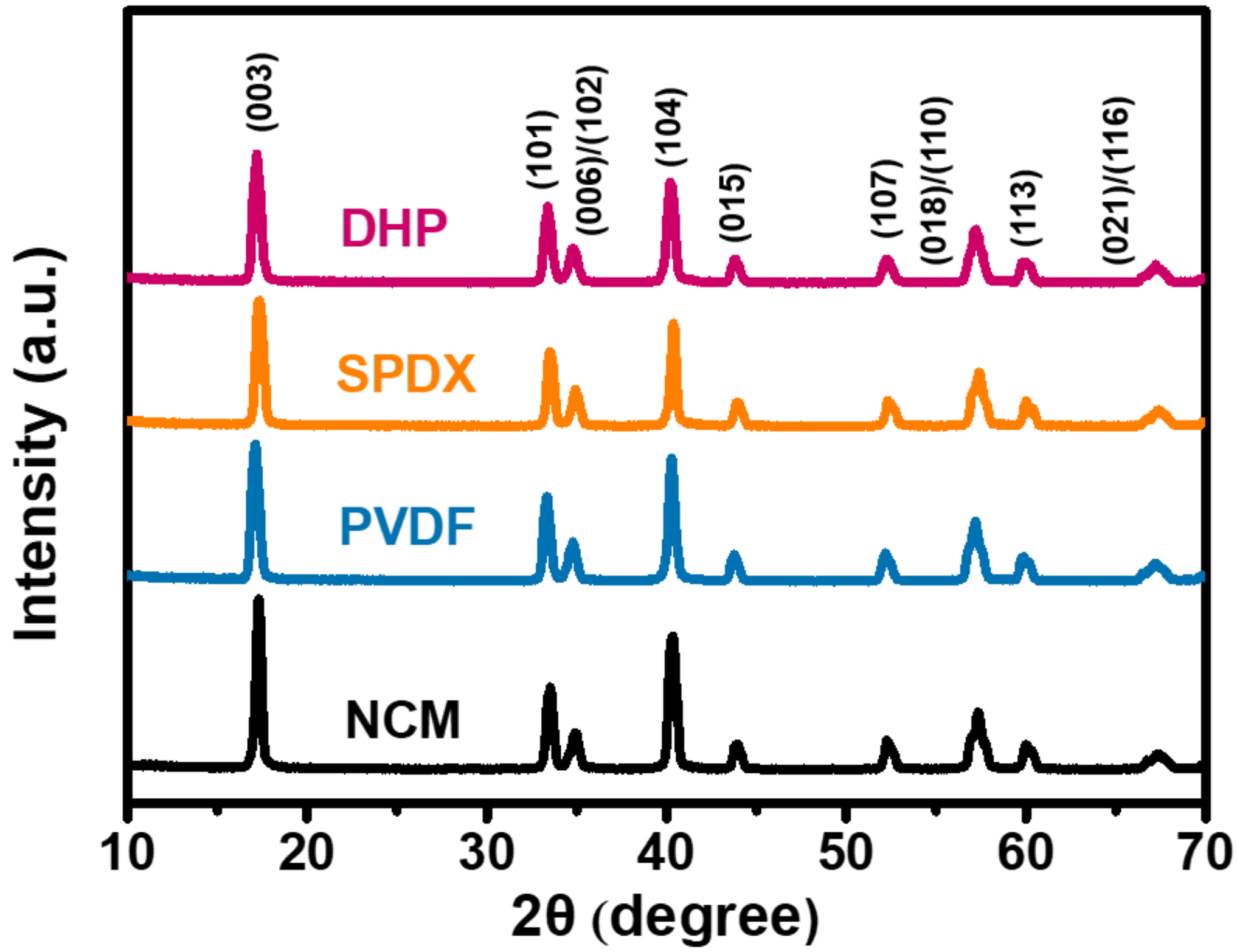

Fig. S5. X-ray diffraction (XRD) patterns of pristine NCM, PVDF, SPDX and DHP electrodes (20 mg cm^{-2}) after activation cycle in Li||NCM cells measured at 0.2 C (1 C = 185 mA g^{-1}) under the voltage window of 2.8–4.3 V.

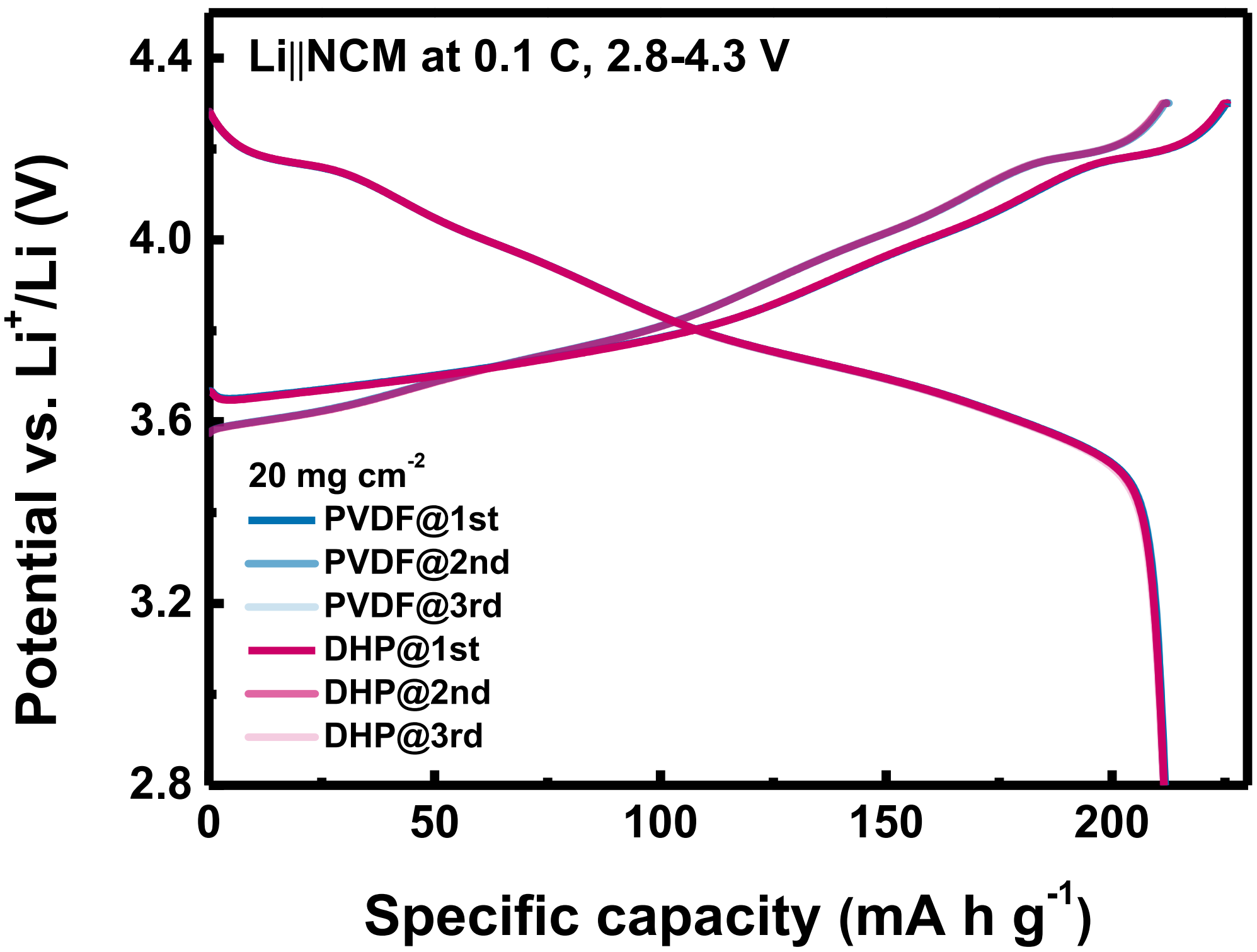

Fig. S6. Galvanostatic charge/discharge profiles of Li||NCM cells employing (a) PVDF and (b) DHP electrodes (20 mg cm^{-2}) during formation cycle at 0.1 C (1 C = 185 mA g^{-1}).

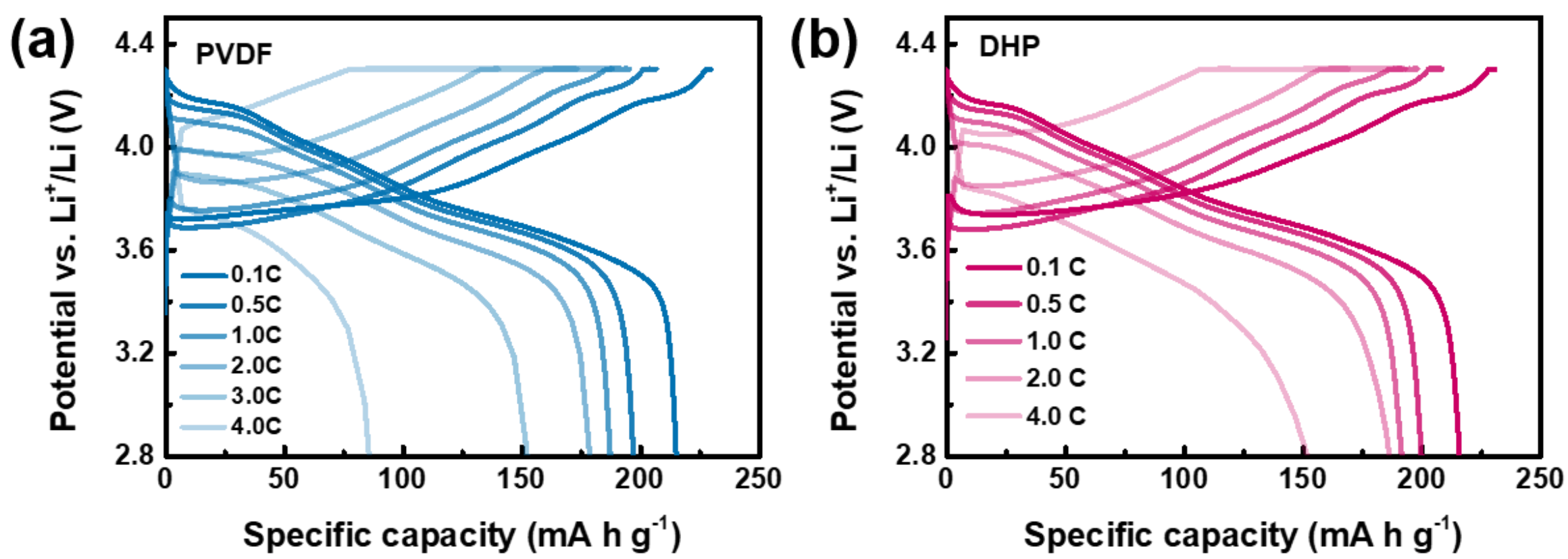

Fig. S7. Galvanostatic charge/discharge profiles of Li||NCM cells employing (a) PVDF and (b) DHP electrodes (20 mg cm^{-2}) under varying discharge current rates of 0.1–4.0 C (1 C = 185 mA g^{-1}).

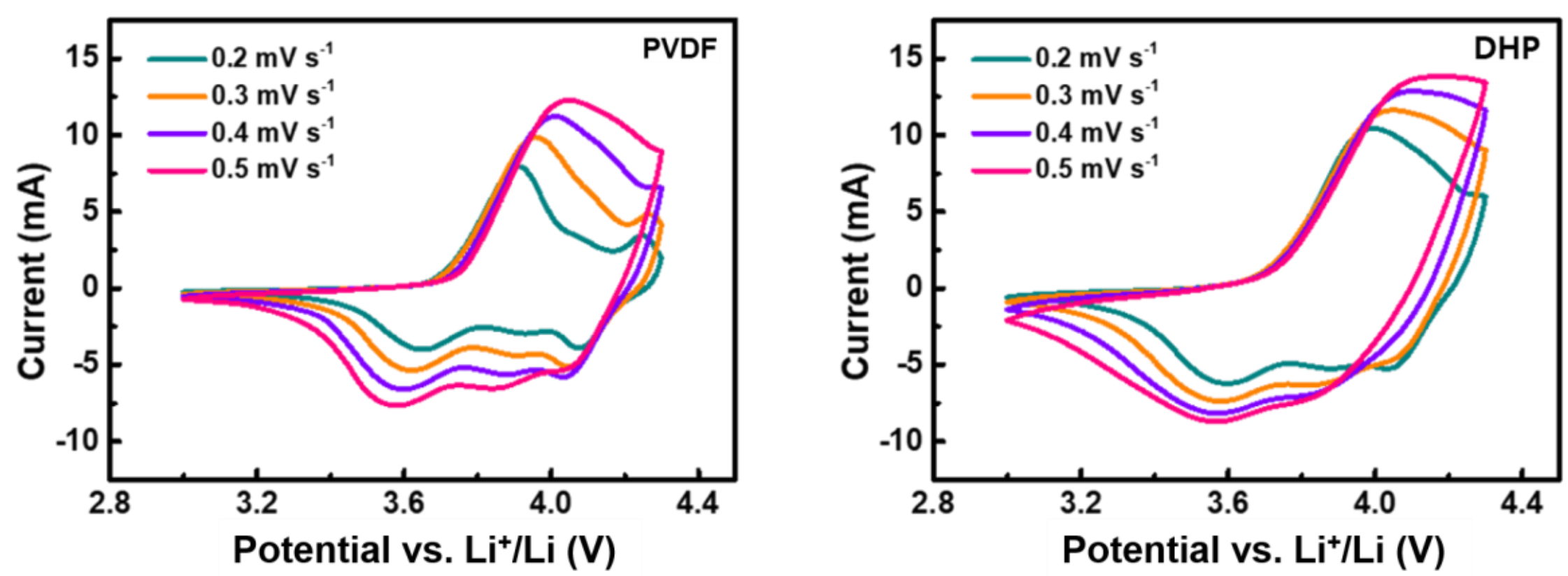

Fig. S8. CV curves of Li||NCM cells employing PVDF and DHP electrodes (20 mg cm^{-2}) at scan rates of 0.2 to 0.5 mV s^{-1}.

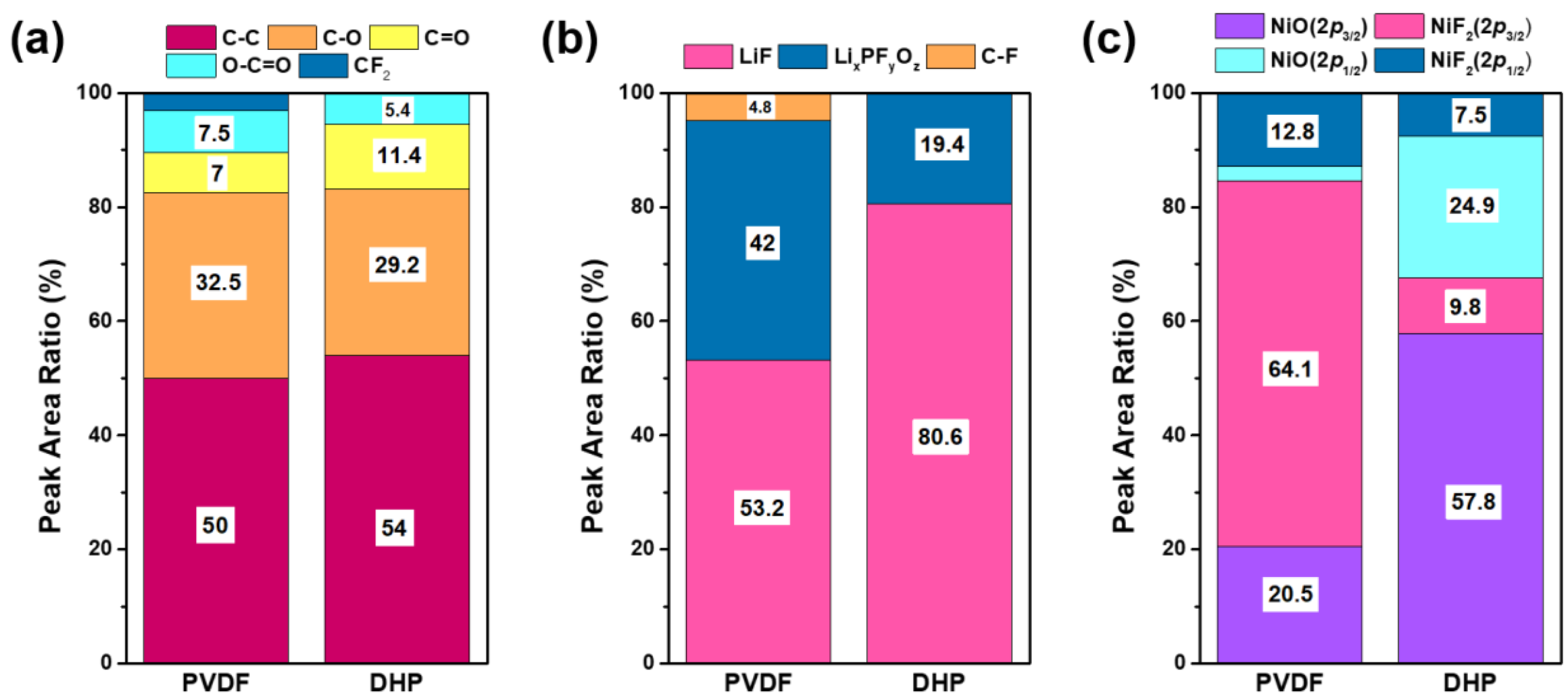

Fig. S9. Peak area ratios obtained from the XPS spectra of (a) C 1*s*, (b) F 1*s*, and (c) Ni 2*p*.

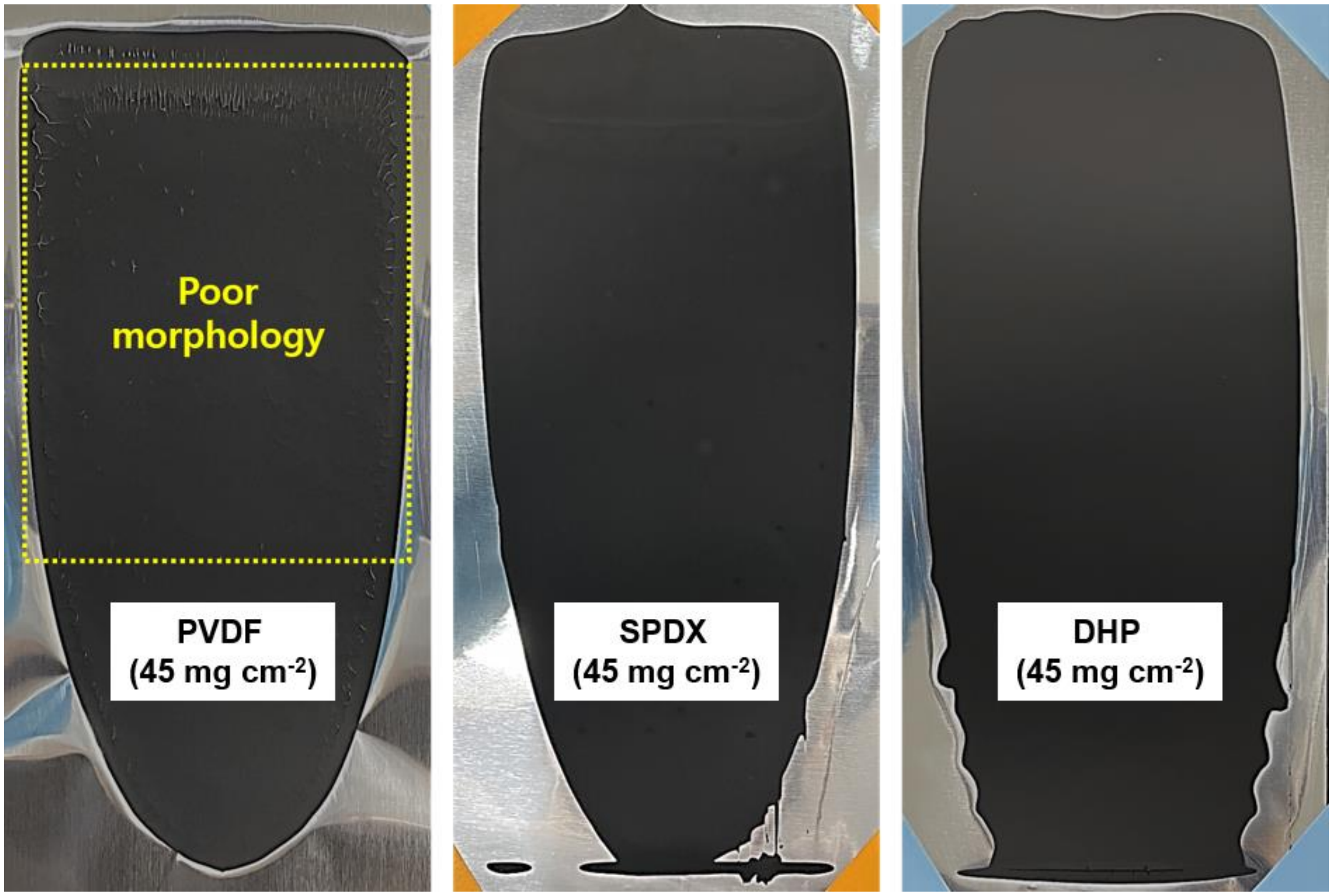

Fig. S10. Photographs of high-mass-loading (45 mg cm^{-2}) PVDF, SPDX, and DHP electrodes (NCM811/Super P/binder = 95/3/2 (w/w/w)).

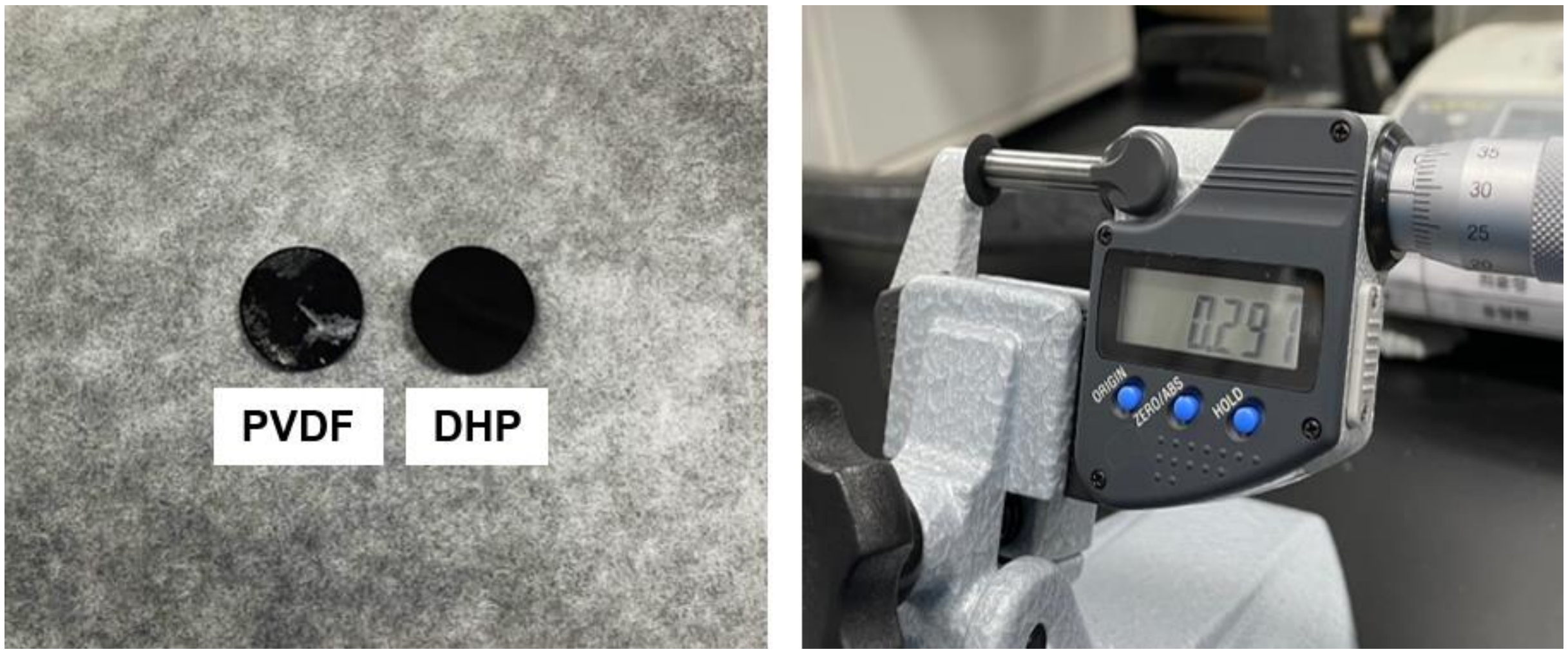

Fig. S11. Photographs of high-mass-loading (70 mg cm^{-2} (corresponding to ≥291 μm)) PVDF and DHP electrodes (NCM811/Super P/binder = 95/3/2 (w/w/w)).

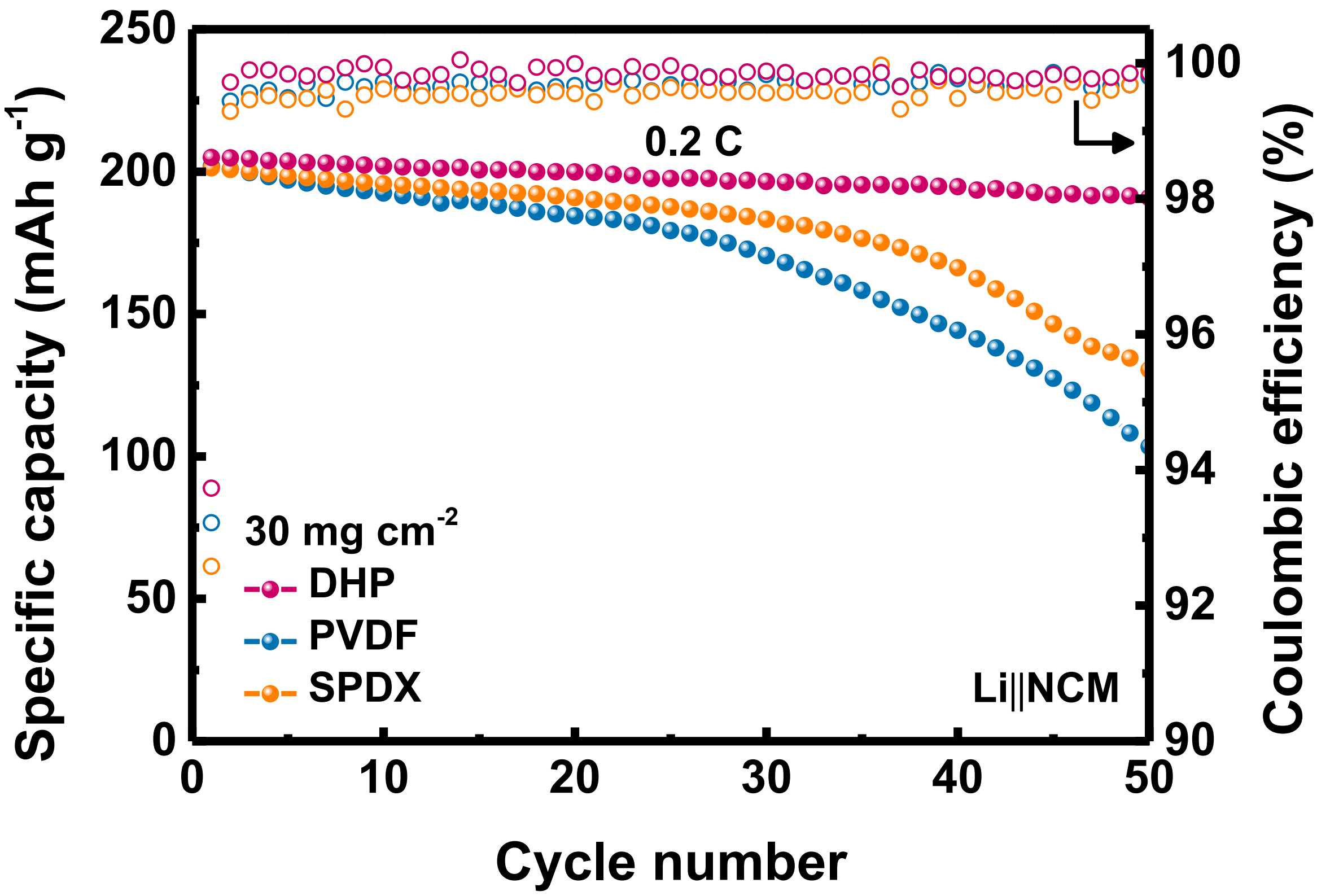

Fig. S12. Cycling performances of the PVDF, SPDX, and DHP electrodes (30 mg cm^{-2}) in Li||NCM cells configuration in the voltage window of 2.8–4.3 V measured at 0.2 C (1 C = 185 mA g^{-1}).

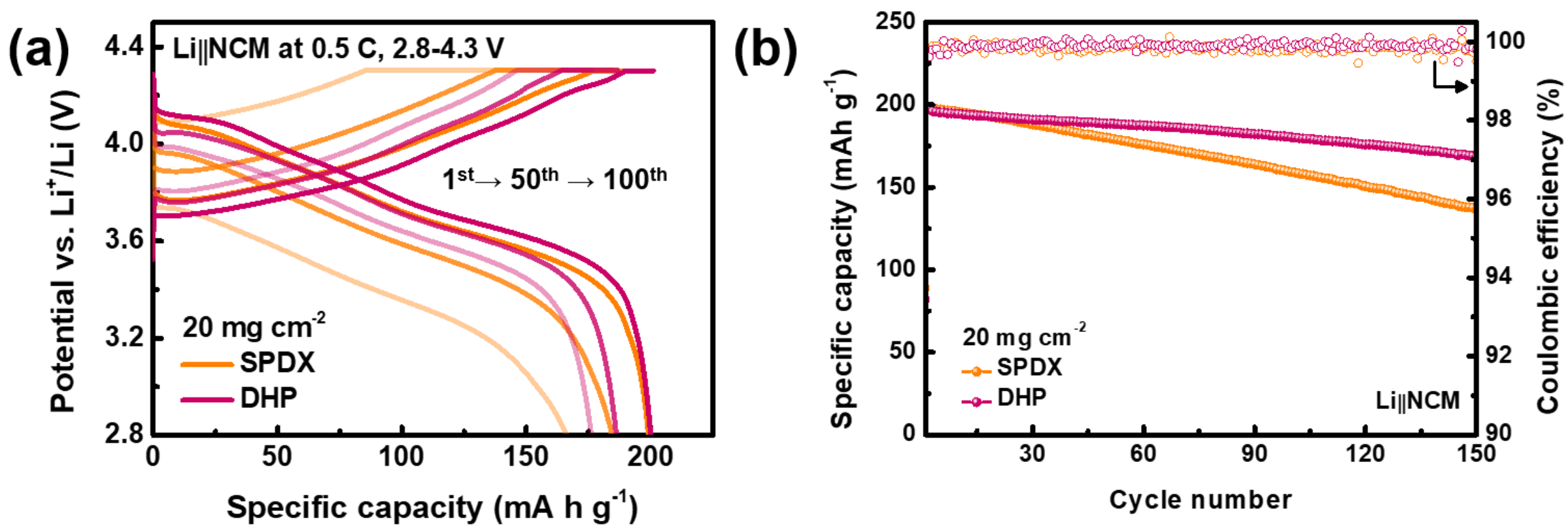

Fig. S13. Electrochemical performances of the SPDX and DHP electrodes in Li||NCM cells configuration. (a) Galvanostatic charge/discharge profiles of the SPDX and DHP electrodes in their first to 100th cycle at 0.5 C (1 C = 185 mA g^{-1}). (b) Cycling performances of both electrodes, presented as discharge capacity, in the voltage window of 2.8–4.3 V measured at 0.5 C.

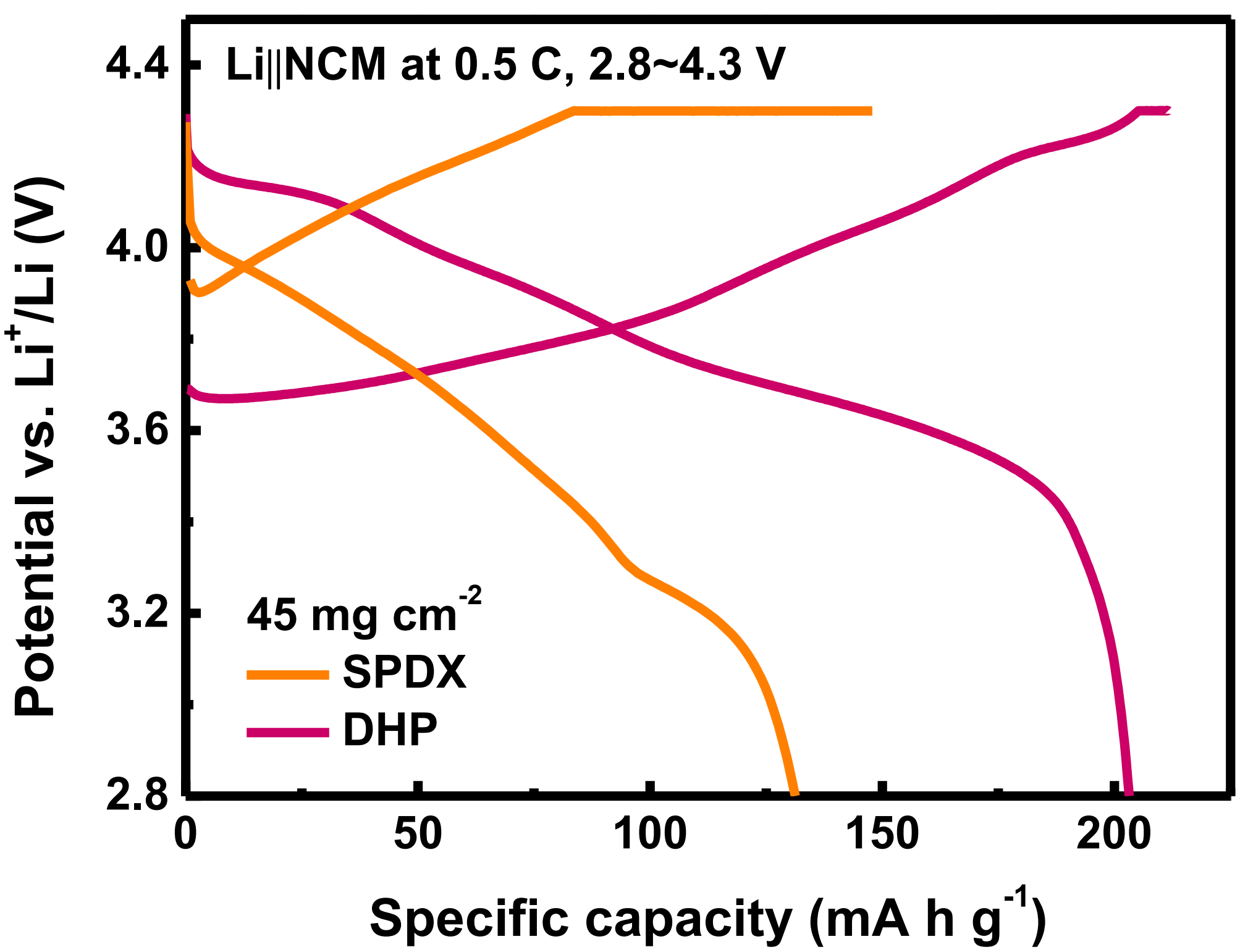

Fig. S14. Galvanostatic charge/discharge profiles of the high-mass-loading SPDX and DHP electrodes (45 mg cm^{-2}) in Li||NCM cells during their first cycle at 0.5 C (1 C = 185 mA g^{-1}).

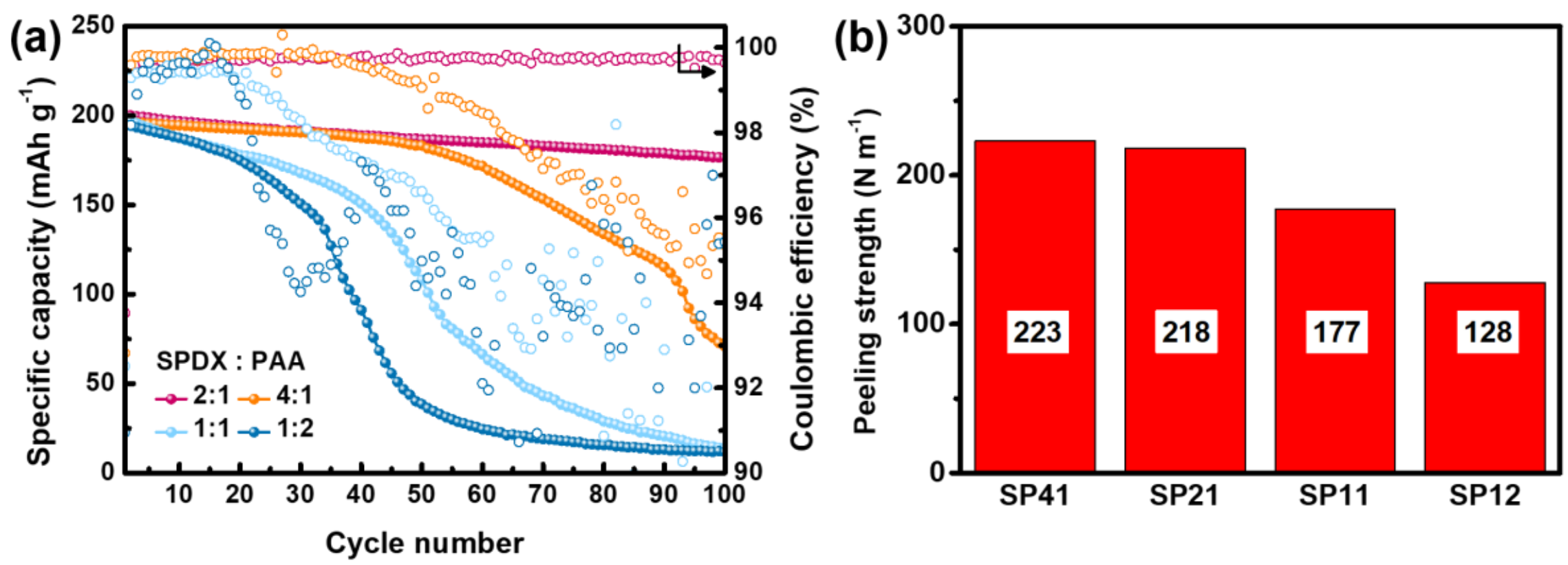

Fig. S15. (a) Cycle performances of Li||NCM cells (20 mg cm^{-2}), presented as discharge capacity, at 0.5 C and (b) 180° peeling tests of 20 mg cm^{-2} DHP electrodes prepared using various combinations of SPDX and PAA binders.

Table S1. Specific energy of the fabricated >10 Ah-class pouch cell (calculated as an estimated value).

Loading level of active material	Stack	Areal capacity (mAh cm^{-2})	Capacity (mAh)	M_p (mg)	M_n (mg)	M_s (mg)	M_a (mg)	M_e (mg)	M_t (mg)	Specific energy (Wh kg^{-1})
**25**	91	5.55	10,101	51,560	20,381	1,950	1043	20,202	340	391
**70**	33	15.5	10,230	49,935	7,383	707	1043	20,460	340	474

$$\text{Specific energy (Wh kg}^{-1}) = \frac{\text{Energy}}{\text{Mass of cell}} = \frac{\text{Nominal voltage x } C}{M_{positive\ electrode} + M_{negative\ electrode} + M_{separator} + M_{tab} + M_{alumium\ pouch} + M_{electrolyte}}$$

M_p = mass of the positive electrode including the Al current collector (3.9 mg cm^{-2}, dimensions of 40 mm × 50 mm)

M_n = mass of the 0.1T Li-metal negative electrode (5.3 mg cm^{-2}) including the Cu current collector (7.3 mg cm^{-2}, dimensions of 45 mm × 55 mm)

M_s = mass of the PE separator (0.8 mg cm^{-2})

M_t = masses of the Ni (170 mg) and Al tab (170 mg)

M_a = mass of Al pouch film (18.04 mg cm^{-2})

M_e = mass of the injected electrolyte (E/C ratio of 2.0 g Ah^{-1})

The cells were composed of positive electrode with various NCM loading values and 0.1T Li metal (40 mAh cm^{-2}, N/P ratio= 1.29 for 70 LL electrode)

The nominal voltage of the cell was set as 3.7 V

Table S2. Comparison of the electrochemical performances between the DHP electrode fabricated in this study and previously reported electrodes containing various binders.

Materials	Active materials	Composition ratio [active materials: conductive agent: binder]	Maximum areal mass loading [mg cm^{-2}]	Maximum areal capacity [mA h cm^{-2}]	Ref.
DHP	$LiNi_{0.8}Co_{0.1}Mn_{0.1}O_2$	95:3:2	70	16.6	This study
PNCI333	$LiNi_{0.8}Co_{0.1}Mn_{0.1}O_2$	90:5:5	21.5	4.0	S1
DPGP-PEI	$LiNi_{0.8}Co_{0.1}Mn_{0.1}O_2$	90:5:5	10	2.0	S2
Cross-linked poly(imidesiloxane)	$LiNi_{0.8}Co_{0.1}Mn_{0.1}O_2$	58.6:3.2:38.2	5.5	1.1	S3
PVDF-CTFE-g-PEGA-co-PAA	$LiNi_{0.8}Co_{0.1}Mn_{0.1}O_2$	93:4:3	5.0	1.1	S4
PI(OBO) and PI(BBP)	$LiNi_{0.8}Co_{0.1}Mn_{0.1}O_2$	94:3:3	9.0	1.8	S5
PAA–PN	$LiNi_{0.8}Co_{0.1}Mn_{0.1}O_2$	80:10:10	2.5	0.5	S6
LTPI	$LiNi_{0.8}Co_{0.1}Mn_{0.1}O_2$	92:5:3	6	1.0	S7
PI-AS44	$LiNi_{0.8}Co_{0.1}Mn_{0.1}O_2$	80:10:10	4	0.8	S8
dextran sulfate sodium-*co*-poly(acrylic acid) lithium (DSS-*co*-PAA(Li))	$LiNi_{0.8}Co_{0.1}Mn_{0.1}O_2$	90:5:5	10	2.0	S9

[S1] D. Jeong, D. S. Kwon, H. J. Kim, J. Shim, Striking a Balance: Exploring Optimal Functionalities and Composition of Highly Adhesive and Dispersing Binders for High-Nickel Cathodes in Lithium-Ion Batteries, *Adv. Energy Mater*. 13 (2023) 2302845, https://doi.org/10.1002/aenm.202302845.

[S2] B. Jin, Z. Cui, A. Manthiram, In situ interweaved binder framework mitigating the structural and interphasial degradations of High-nickel cathodes in lithium-ion batteries, Angew. *Chem. Int. Ed.* 62 (2023) e202301241, https://doi.org/10.1002/ange.202301241.

[S3] Y. Wang, N. Dong, B. Liu, G. Tian, S. Qi, D. Wu, Self-adaptive Gel Poly(imide-siloxane) Binder Ensuring Stable Cathode-Electrolyte Interface for Achieving High-

Performance NCM811 Cathode in Lithium-ion Batteries, *Energy Storage Mater*. 56 (2023) 621–630, https://doi.org/10.1016/j.ensm.2023.02.002

[S4] T. Liu, R. Parekh, P. Mocny, B. P. Bloom, Y. Zhao, S. Y. An, B. Pan, R. Yin, D. H. Waldeck, J. F. Whitacre, K. Matyjaszewski, Tailored PVDF graft copolymers via ATRP as high-performance NCM811 cathode binders, *ACS Mater. Lett.* 5 (2023) 2594–2603, https://doi.org/10.1021/acsmaterialslett.3c00485.

[S5] K. Qi, Y. Wang, N. Dong, B. Liu, G. Tian, S. Qi and D. Wu, Novel polyimide binders integrated with soft and hard functional segments ensuring long-term high-voltage operating stability of high-energy NCM811 lithium-ion batteries up to 4.5 V, *Appl. Energy* 320 (2022) 119282, https://doi.org/10.1016/j.apenergy.2022.119282.

[S6] H. Wang, F. Zhang, N. Qin, Z. Wang, Y. Wang, Z. Wang, C. Zeng, H. Li, Q. Liu, Y. Li, Z. Lu, D. Luo, H. Cheng, Sodium Phytate Cross-Linked Polyacrylic Acid as Multifunctional Aqueous Binder Stabilizes LiNi0.8Co0.1Mn0.1O2 to 4.6 V, *ACS Energy Lett.* 10 (2025) 136–144, https://doi.org/10.1021/acsenergylett.4c02796.

[S7] C. Q. Wang, Y. J. Kang, B. X. Liu, D. L. Lin, X. Y. Zhao, Z. H. Chen, S. P. Li, Low-Temperature Imidized Polyimide Binder Enabling Structural and Electrochemical Stability of NCM811 Cathode for High-Safety and Long-Cycling Lithium-Ion Batteries, *ACS Sustainable Chem. Eng.* 13 (2025) 7687-7697, https://doi.org/10.1021/acssuschemeng.5c02283.

[S8] L. Chen, D. Yang, J. Xin, X. Zhou, Y. Zhang, Z. Lv, T. Guo, Q. Chen, R. Gao, Y. Wang, N. Wang, S. Kang, N. Li, Z. Xu, Q. Zhang, High dielectric sulfonyl-containing polyimide binders optimize the long-term stability and safety of NCM811 lithium-ion

batteries at high voltages, *Chem. Eng. J.* 503 (2025) 158670, https://doi.org/10.1016/j.cej.2024.158670.

[S9] J. Chen, K. Hu, Z. Wang, H. Xu, Y. Huang, X. Hu, Enhancing Stability and 6C Fast Charging in μSi||LiNi0.8Co0.1Mn0.1O2 Lithium-Ion Batteries Using Conductive Binders With Multiple Hydrogen Bonds, *Small* 21 (2025) e2410118, https://doi.org/10.1002/smll.202410118.
