# Supplementary material for: Wet-processed high-areal-capacity electrodes via transformative spandex–poly(acrylic acid) binder toward 450 Wh kg−1 lithium-ion batteries
Source: Nat Commun. 2026 Jul 23;17:8986. doi: 10.1038/s41467-026-76014-4 (PMC13507357; doi:10.1038/s41467-026-76014-4)
Supplement: Supplementary file 2 [file 41467_2026_76014_MOESM2_ESM.pdf]

nature portfolio

Peer Review File

Wet-processed ultra-high-areal-capacity electrodes via transformative spandex–poly(acrylic acid) binder toward 450 Wh kg-1 lithium-ion batteries

Corresponding Author: Professor Ki Jae Kim

This file contains all reviewer reports in order by version, followed by all author rebuttals in order by version.

Version 0:

Reviewer comments:

Reviewer #1

(Remarks to the Author)
In this work, the authors report a SPDX-PAA composite binder (DHP) to enhance the electrochemical performance of NCM811 cathode for lithium-ion batteries. Compared to the traditional PVDF binders, DHP exhibits superior viscosity and adhesion, enhancing the uniformity and peel strength of thick NCM811 electrodes. Additionally, DHP can form a thin and uniform coating layer on the NCM811 surface, thereby mitigating surface degradation and transition metal ion dissolution while promoting lithium-ion transport. NCM811 cathodes with a mass loading of 40 mg cm-2 were successfully fabricated using DHP binder. Pouch cells prepared with this cathode exhibited excellent cycling stability. This job contains some important results that merit publication. However, the manuscript contains some vague and controversial statements, and there are some comments and suggestions for the authors. So I would recommend major revisions.
1, On page 3, line 85, the author mentions that binder migration poses serious challenges for the preparation of thick electrodes. So, what causes the migration of the binder during the drying process? This is crucial for understanding how DHP binders address the binder migration issue.
2, On page 4, line 128, the author suggests that SPDX's abundant hydrogen-bonding functional groups confer a strong affinity toward high-nickel cathodes. However, compared to SPDX, PAA contains more carboxyl functional groups that exhibit stronger hydrogen-bonding interactions. In fact, besides providing lithium-ion conductivity, the carboxyl groups can also form hydrogen bonds with the cathode surface. Therefore, how does the author understand the contribution of PAA to surface protection?
3, On page 6, line 174, the author claims that the peaks corresponding to N-H bending and C=O stretching were shifted to 1664 and 1506 cm-1, respectively, indicating hydrogen-bonding and crosslinking network formation. However, these peak positions cannot be correlated with N-H bending and C=O stretching signals in SPDX, and shifts in different directions fail to prove hydrogen bond formation.
4, On page 9, line 261, the author claims that the CV peak around 4V during the first charge-discharge cycle represents the H+/Li+ exchange reaction in PAA. However, NCM811 also undergoes delithiation at this potential, raising the question of how to confirm the occurrence of H+/Li+ exchange.
5, The scale text in Figure 5g is too small and differs from that in other diagrams.

Reviewer #2

(Remarks to the Author)
This manuscript reports the development of an aqueous and dual-functional hybrid polymer (DHP) binder composed of spandex (SPDX) and poly(acrylic acid) (PAA), enabling the fabrication of ultra–high–mass–loading Ni-rich cathodes (up to 70 mg cm^{-2}) via a conventional wet process. The study demonstrates significantly improved adhesion strength, Li^+ transport kinetics, and structural integrity, resulting in outstanding areal capacities and an impressive pouch-cell energy density of 451 Wh kg^{-1}. Overall, the work presents a technically robust and industrially potential approach. However, several critical issues require clarification and additional validation before the manuscript can be considered for publication. The study would benefit from deeper mechanistic insights, more transparent data presentation, and further control experiments to reinforce the reliability of the conclusions.

1. The authors should clearly elucidate the distinct roles of PAA and SPDX within the DHP system and explain why each

single-component binder fails to achieve comparable performance. It is strongly recommended to include electrochemical results for electrodes using only PAA and only SPDX as controls, to verify the claimed synergistic effect.

2. As TEM images serve as essential evidence supporting the homogeneous distribution of the binder, Figures 2(a) and 2(d) are too indistinct to substantiate this claim. The authors should provide higher-resolution TEM images or complementary microscopic characterization (e.g., AFM or STEM mapping) to strengthen the conclusion regarding uniform binder dispersion.

3. Although the authors propose that Li–PAA is formed at the interface based on XPS and CV analyses, these are indirect indicators. The manuscript would be substantially strengthened by post-cycling FTIR or solid-state NMR studies, which can directly confirm the chemical bonding evolution and validate the presence of Li–PAA.

4. The interpretation of the Li_2CO_3 signal as originating from a "native CEI layer" (line 283) is not sufficiently justified. It is more plausible that the signal arises from residual alkaline species remaining on the NCM particle surface after synthesis. Therefore, the Li–PAA formation should be reinterpreted as an interfacial reaction between PAA and these surface residues, rather than with a pre-formed CEI layer.

5. Since peeling strength is inherently dependent on the specimen width, the adhesion results in Figure 2(g) should be normalized by width and expressed in N m^{-1}, to ensure quantitative consistency and fair comparison among different samples.

6. To facilitate a more convincing comparison of CEI compositions, the authors are encouraged to provide fitted peak areas and/or atomic ratios derived from XPS spectra. Such quantitative data would enhance the credibility and clarity of the compositional analysis.

7. The cycling performance of the 70 mg cm^{-2} electrode, limited to only 25–50 cycles, is insufficient to substantiate the claimed long-term stability. The authors should extend the cycling test to at least 200 cycles or include post-mortem structural characterization to confirm durability. Additionally, to eliminate possible artifacts from Li-metal degradation, full-cell tests using a more stable counter electrode (e.g., Si-based or graphite anode) are strongly recommended.

Reviewer #3

(Remarks to the Author)
Review of NCOMMS-25-79709

In this article the authors report a new promising binder allowing the wet-processing of very high areal capacity positive electrodes, which would allow the obtaining of lithium-ion batteries with higher energy density, which is sought by the battery industry. This new binder also shows promising properties allowing for a longer cyclability of Ni-rich cathode material by helping better cycling stability of the structure of such cathode material. Overall the results presented by the authors are making sense. However, for convincing the reader some complementary data or experiments are required as below:

Major modifications :
- In Figure 1b, the PAA spectrum is unexpected, as the characteristic bands that include broad O-H stretching in the 3500 to 2500 cm-1, and CH2 asymmetric and symmetric stretching at 2950 and 2800 cm-1 are strikingly lacking
- The description of the preparation of both positive and negative electrodes is far from being complete: protocole (steps, duration of each setp, equipment used for each step). All the important experimental details necessary to reproduce the work must be given (dry solid content of the slurries, doctor blade gap, SPDX provider and SPDX characteristics such as molar mass, Graphite powder & CMC & SBR providers and characteristics, current collectors thicknesses)
- The description of the pouch cells is also laconic, as well as the samples preparations for characterization techniques, in particular port-mortem ones. More information must be given.
- SEM, TEM, EDS visualisation of the morphology of the pristine and cycled electrodes depending on the binder are thoroughly used to assess the advantages of the new binder. It is well known that such observations are subjective and dependent on the sampling. However, only one image is generally shown per technique and sample (eg. in Fig. 2a-c and in Fig. 5 a-i and in Fig. S2). Complementary images taken at various spots of the sample must be shown in supporting information as element of proof. At least 2 more per characterization techniques.
- In Figure S2 the electrodes appear non porous or having very few porosities. What are the electrodes porosities ? Is it possible to measure the distribution in pore size ?
- In Figure 3b, one see a strong peak during the 1st lithiation for the new binder that is attributed to an in situ ion exchange reaction H+/Li+ occurring between the NCM cathode and the PAA of the new binder. Could the authors calculate the amount of electric charges associated (consummed) with this peak and relate it to the amount of available H+ from the -COOH moities of PAA (given its mass quantity in the electrode composition) to assess their interpretation ?
- In Figure 3e-h, the intensity of the Li 1s peak is used to assess the formation of Li-PAA. The intensity of this peak must be sensitive to the XPS acquisition conditions, which is not described in the experimental method. Futhermore, what normalization procedure was adopted to secure a fair comparison between the samples ?
- Figure 4b-c and 7g,j,k : reproducibility must be evaluated and results shown for at least 3 identical cells
- The satisfactory cyclability of DHP cathode half and full cells are shown. However, what are they rate performance (in

particular for full cells) ? Indeed, there is little interest of achieving good cyclability of very high areal loading of electrodes if they cannont achieve good performance for example @1C.

Minor modifications :
- In their introduction, the authors cite the dry processing methods utilizing PTFE that can allow achieving very high areal capacity electrodes. Some very promising results were also obtained through such dry-processing methods but utilizing PVdF, which can be acknowledged, see for example J. A. Barreras-Uruchurtu, N. Besnard, C. Paul, L. Marchal, S. Devisme, B. Lestriez, "Effect of the physicochemical properties of PVdF on dry-sprayed graphite electrodes for lithium-ion batteries for electric vehicle applications", J. Electrochem. Soc., 2024, 171, 12053
- The peel strength results must be given in N/m or N/cm
- After 150 cycles, capacity retention of the PVDF electrode was measured to be 71.9%, which is low compared to the state of the art for a NMC811/PVdF electrode and may results from unoptimized preparation of the electrode or unsuitable PVdF grade. This should be discussed.
- Page 14 : the authors write that the new binder suppresses the structural changes in NMC811, such as volume expansion during repeated lithiation and delithiation. This is not possible as lithiation and delithiation must result in volume expansion due to Li insertion and desinsertion in the crystalline host structure of NMC811. However, the new binder may prevent the cracking mechanism in a certain way.

Reviewer #4

(Remarks to the Author)
This manuscript reports development of dual binder system to enable thick electrodes with improved performance. Below are some comments for improvement.
1) In the introduction section, the authors ascribed challenges in thick electrodes to binder migration during electrode drying. However, same process was used with the dual binder system. Why isn't the binder migration a problem any more?
2) The authors performed a series of postmortem analysis and ascribed the difference to the binder. However, how does the binder affect cation mixing in NMC?
3) Miscroscopy images could be misleading. In Fig S2, there seem more particle cracks in the DHP electrode than that PVDF electrode, which is contradictory to the statement in the context.
4) The authors mentioned that NMC particles were fully covered by the dual binder. The electrodes would be more resistive similar to that after 100 cycles in figure 4d. However, why was the overpotential lower compared to the PVDF electrode as shwon in Fig 4a.
5) The impedance unit in Fig 4 d is incorrect.
6) Since there Li-H exchange, what's the coulombic efficiency in the first cycle, particularly in full cells?
7) Fabricating 45 mg/cm2 NMC cathode with PVDF as binder should not be a problem especially with doctor blade as shown in Fig. S7. The baseline was very poor.
8) In Table S1, the tabs will be much larger for 10 Ah cells and should be much more heavier.

Version 1:

Reviewer comments:

Reviewer #1

(Remarks to the Author)
The improvements are substantial and the revised manuscript can be accepted for publication in Nat. Commun.

Reviewer #2

(Remarks to the Author)
The responses are appropriate and the revised manuscript can be accepted.

Reviewer #4

(Remarks to the Author)
The authors have addressed my comments.

Open Access This Peer Review File is licensed under a Creative Commons Attribution 4.0 International License, which permits use, sharing, adaptation, distribution and reproduction in any medium or format, as long as you give appropriate credit to the original author(s) and the source, provide a link to the Creative Commons license, and indicate if changes were made.
In cases where reviewers are anonymous, credit should be given to 'Anonymous Referee' and the source.
The images or other third party material in this Peer Review File are included in the article's Creative Commons license, unless indicated otherwise in a credit line to the material. If material is not included in the article's Creative Commons license and your intended use is not permitted by statutory regulation or exceeds the permitted use, you will need to obtain permission directly from the copyright holder.
To view a copy of this license, visit https://creativecommons.org/licenses/by/4.0/

SungKyunKwan University

Dept. of Energy Science
Natural Sciences Campus, 2066, Seobu-ro, Jangan-gu,
Suwon-si, Gyeonggi-do, Republic of Korea (16419)

March 02, 2026

Dear Editor and Reviewers

Thank you for considering our manuscript entitled, "***Wet-processed ultra-high-areal-capacity electrodes via transformative spandex–poly(acrylic acid) binder toward 450 Wh kg^{-1} lithium-ion batteries***" for publication in ***Nature Communications***. The manuscript has now been revised according to the reviewers' suggestions. The changes are summarized and highlighted in yellow in the revised manuscript. The suggestions made by the reviewers have enabled the creation of a much more informative manuscript, and we again thank them for their excellent comments. We believe that these revisions have significantly improved the quality and readability of the manuscript. Again, we thank you for the opportunity to revise the manuscript.

Sincerely,

Ki Jae Kim, Ph.D./Professor

Department of Energy Science

Sungkyunkwan University

2066, Seobu-ro, Jangan-gu, Suwon-si, Gyeonggi-do, 16419, Republic of Korea

E-mail: kijaekim@skku.edu

Responses to the comments by Reviewer #1:

In this work, the authors report a SPDX-PAA composite binder (DHP) to enhance the electrochemical performance of NCM811 cathode for lithium-ion batteries. Compared to the traditional PVDF binders, DHP exhibits superior viscosity and adhesion, enhancing the uniformity and peel strength of thick NCM811 electrodes. Additionally, DHP can form a thin and uniform coating layer on the NCM811 surface, thereby mitigating surface degradation and transition metal ion dissolution while promoting lithium-ion transport. NCM811 cathodes with a mass loading of 40 mg cm-2 were successfully fabricated using DHP binder. Pouch cells prepared with this cathode exhibited excellent cycling stability. This job contains some important results that merit publication. However, the manuscript contains some vague and controversial statements, and there are some comments and suggestions for the authors. So I would recommend major revisions.

Comment 1

On page 3, line 85, the author mentions that binder migration poses serious challenges for the preparation of thick electrodes. So, what causes the migration of the binder during the drying process? This is crucial for understanding how DHP binders address the binder migration issue.

Our response

Thank you for your valuable comments. Binder migration toward the electrode surface is primarily driven by capillary forces generated during solvent evaporation, which induce solvent flow toward the drying surface and promote binder redistribution [R1]. This

phenomenon becomes more significant in thick electrodes because increased electrode thickness requires longer drying times for complete solvent removal, thereby allowing more extensive capillary-driven transport and increasing binder accumulation at the surface [R2]. As a result, the adhesion between the Al current collector and the electrode film can deteriorate, and the accumulated binder materials on the electrode surface may severely hinder lithium-ion transport. The proposed DHP suppresses binder migration through two primary mechanisms. First, compared with PVDF, DHP forms a significantly high-viscosity slurry (380 Pa·s vs 106 Pa·s for the PVDF slurry at a shear rate of 250 s^{-1}, as shown in Fig. S1). Since binder migration is generally more severe in low-viscosity slurries due to enhanced convective flow, DHP is effective in reducing binder migration under the identical solvent conditions [R3]. Second, the functional groups of DHP can interact strongly with both the Al current collector and the electrode materials, providing enhanced adhesion and cohesion. These strong interactions improve binder immobilization during solvent evaporation, thereby mitigating capillary-driven redistribution and suppressing binder migration [R1,4]. To improve the readers' understanding, a detailed explanation of the origin of binder migration has been added to the Introduction section of the revised manuscript, as shown below.

[R1] Chang, J. H. et al. Binder migration: Frequently observed yet overlooked phenomena in electrode processing for lithium-ion batteries, *J. Energy Storage* **83**, 110729 (2024).

[R2] Park, K. et al. Mitigation of binder migration behavior during the drying process by applying an electric field for fast-charging in lithium-ion batteries, *Batteries & Supercaps* **6**, 2300170 (2023).

[R3] Klemens, J. et al. Drying of NCM cathode electrodes with porous, nanostructured particles versus compact solid particles: Comparative study of binder migration as a function of drying

conditions, *Energy Technol.* **10**, 2100985 (2022).

[R4] Heo, S.-W. et al. Core-shell structure of polyacrylate-based binder enhancing the wet-adhesion strength and the kinetics of lithium-ion diffusion, *Electrochim. Acta* **455**, 142387 (2023).

Correction in the revised manuscript

1) Correction in Introduction section.

The main challenges associated with thick electrodes primarily stem from binder migr ation during the drying process and limited electrolyte penetration caused by the incre ased electrode thickness.[17,18,19,20] Binder migration toward the electrode surface is drive n by capillary forces generated during solvent evaporation during the drying process a nd becomes more severe in thicker electrodes owing to prolonged solvent removal. Th is phenomenon leads to delamination by weakening the adhesion between the electrod e and current collector and causes agglomeration of the binder and conductive materia ls, significantly hindering the lithium-ion and electron conductivity of the electrode.[17,18] Moreover, poor electrolyte penetration, caused by the formation of a thick binder laye r on the electrode surface due to binder migration, further impedes lithium-ion mobilit y within the electrode.[19,20]

Comment 2

On page 4, line 128, the author suggests that SPDX's abundant hydrogen-bonding functional groups confer a strong affinity toward high-nickel cathodes. However, compared to SPDX,

PAA contains more carboxyl functional groups that exhibit stronger hydrogen-bonding interactions. In fact, besides providing lithium-ion conductivity, the carboxyl groups can also form hydrogen bonds with the cathode surface. Therefore, how does the author understand the contribution of PAA to surface protection?

Our response

We greatly appreciate your valuable comments on our work. As the reviewer pointed out, PAA contains abundant carboxyl groups and therefore may form hydrogen bonds with the cathode surface. However, the effectiveness of surface protection is not solely determined by the number of hydrogen-bonding functional groups, but also by the binder's ability to form a uniform interfacial network and maintain mechanical integrity during cycling. As shown in Fig. S3, due to the rigid and brittle nature of PAA, the PAA electrode exhibited surface cracking and weakened interparticle adhesion after drying. Such brittle mechanical properties are known to cause structural instability and pose challenges in preventing electrode cracking during cycling in NCM electrodes [R5-6]. Compared to PAA, SPDX can form a more homogeneous and conformal coverage on the surface of NCM particles, enabling effective interfacial interactions with both the Al current collector and adjacent NCM particles. The uniform coverage promotes strong particle-to-particle cohesion and particle-to-collector adhesion, thereby contributing to structural stability. In this regard, SPDX plays a more significant role in surface protection by establishing a uniform interfacial network and preserving the mechanical integrity of the electrode. However, since SPDX extensively covers the NCM particles, this conformal coverage may increase interfacial resistance and induce cell polarization, particularly in thick electrodes. Consistently, the SPDX electrode showed more severe polarization in the voltage profiles and faster capacity decay during cycling than the DHP electrode (Fig. S10 and S11).

Therefore, within the DHP network, PAA was incorporated as a complementary component at half the proportion of SPDX to improve Li^+ transport properties rather than primarily reinforce surface protection, while SPDX served as the backbone component to protect the electrode surface and enhance mechanical robustness.

[R5] Pillai, A. M. et al. Aqueous binders for cathodes: A lodestar for greener lithium ion cells, *Energy & Fuels* **36**, 5063–5087 (2022).

[R6] Wang, H. et al. Sodium phytate cross-linked polyacrylic acid as multifunctional aqueous binder stabilizes LiNi0.8Co0.1Mn0.1O2 to 4.6 V, *ACS Energy Lett.* **10**, 136–144 (2025).

Comment 3

On page 6, line 174, the author claims that the peaks corresponding to N-H bending and C=O stretching were shifted to 1664 and 1506 cm^{-1}, respectively, indicating hydrogen-bonding and crosslinking network formation. However, these peak positions cannot be correlated with N-H bending and C=O stretching signals in SPDX, and shifts in different directions fail to prove hydrogen bond formation.

Our response

Thank you for your valuable comments. Upon careful reviewing the reviewer's comments, we found that the notations for N–H bending and C=O stretching were mistakenly reversed in the manuscript, and that the explanation of the hydrogen bonding interaction was not sufficiently clear. We sincerely apologize for any confusion arising from our previous description. As the

reviewers noted, hydrogen bond formation typically results in a shift toward lower wavenumbers, because hydrogen bonding leads to bond elongation and a decrease in the force constant, resulting in a red shift to lower wavenumbers in the FT-IR spectrum. We believe that the confusion may have arisen from the lack of clarity in our previous description. In the FT-IR spectrum of the DHP film, the C=O stretching peak of PAA shifts from 1695 cm^{-1} to 1664 cm^{-1}, and that the corresponding peak of SPDX shifts from 1539 cm^{-1} to 1506 cm^{-1}, both exhibiting a red shift to lower wavenumber, which is consistent with hydrogen bond formation. To prevent any misunderstanding among readers, Fig. 1b has been revised to more clearly highlight these peak shifts, and the related explanation in the manuscript has been revised as follows. We once again appreciate the reviewer's valuable comments, which have contributed to improving the quality of our work.

Correction in the revised manuscript

1) Correction in Results and Discussion section.

For DHP, the characteristic peaks of SPDX and PAA were observed, confirming the presence of both polymers. Notably, the C=O stretching peak of PAA shifts from 1695 cm^{-1} to 1664 cm^{-1}, and that the corresponding peak of SPDX shifts from 1539 cm^{-1} to 1506 cm^{-1}, respectively, in the DHP spectrum. In addition, the N–H stretching vibration of SPDX shifts from 3320 cm^{-1} to 3315 cm^{-1} toward a lower vibrational frequency. These shifts indicate successful crosslinking via hydrogen-bonding interactions between the urea and urethane functional groups of SPDX and the carboxyl group of PAA, as illustrated in Fig. 1a.[45]

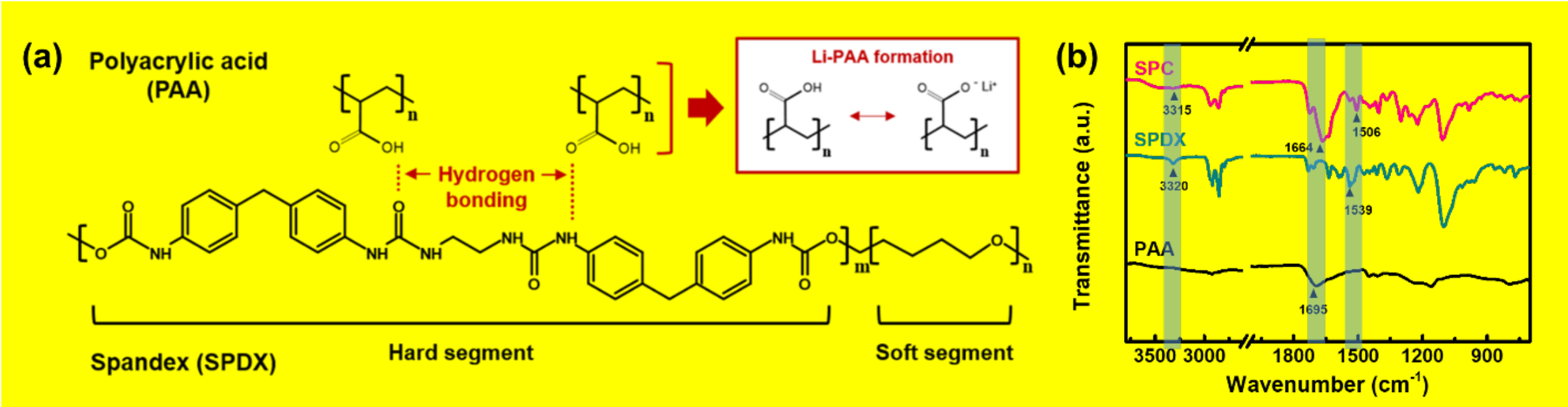

Fig. 1. (a) Chemical formulas of SPDX and PAA with hydrogen bonding interactions and Li-PAA formation by H^+/Li^+ exchange to stabilize the surface structure. (b) Fourier transform infrared (FTIR) spectroscopy of PAA, SPDX, and DHP binder films.

Comment 4

On page 9, line 261, the author claims that the CV peak around 4V during the first charge-discharge cycle represents the H+/Li+ exchange reaction in PAA. However, NCM811 also undergoes delithiation at this potential, raising the question of how to confirm the occurrence of H+/Li+ exchange.

Our response

We greatly appreciate your valuable comments on our work. As the reviewer pointed out, since the voltage region near 4 V corresponds to the delithiation of NCM811, the initial oxidation profile of the DHP electrode inevitably includes contributions from both Li-PAA formation and NCM delithiation, which necessitates a clear confirmation of the H^+/Li^+ exchange reaction. As shown in Fig. 3a and b, the DHP and PVDF electrodes exhibit markedly different CV curves during the first oxidation process, and this discrepancy is limited to the first cycle, whereas similar peak shapes and positions are observed from the second cycle. Considering this behavior, the difference in the first oxidation suggests that the DHP electrode undergoes an additional irreversible electrochemical process that does not occur in the PVDF electrode.

These characteristics are consistent with the reported Li-PAA formation via the H^+/Li^+ exchange reaction of PAA during the activation cycle, as described in previous studies employing PAA as a binder [R7-9]. In this study, the H^+/Li^+ exchange reaction was further confirmed via XPS analysis, which revealed the formation of enriched -COOLi (Li-PAA) species on the surface of the DHP electrode after the activation cycle compared to the pristine DHP electrode.

To more directly verify the evolution of Li–PAA associated with the H^+/Li^+ exchange reaction during the first charging process, post-cycling FT-IR analysis was performed on the DHP electrode in both the pristine state and after the initial charging. As shown in **Fig. R1**, the pristine DHP electrode exhibits a –COOH peak originating from PAA. This peak disappears after the initial charging process, accompanied by the formation of a –COOLi peak at around 1550 cm^{-1}. The disappearance of the –COOH signal and the concurrent emergence of the –COOLi provide direct spectroscopic evidence of the H^+/Li^+ exchange reaction of PAA. In addition, to clearly provide evidence of the H^+/Li^+ exchange reaction, the post-cycling FT-IR analysis results have been added to the revised manuscript and the Supporting Information, as shown below.

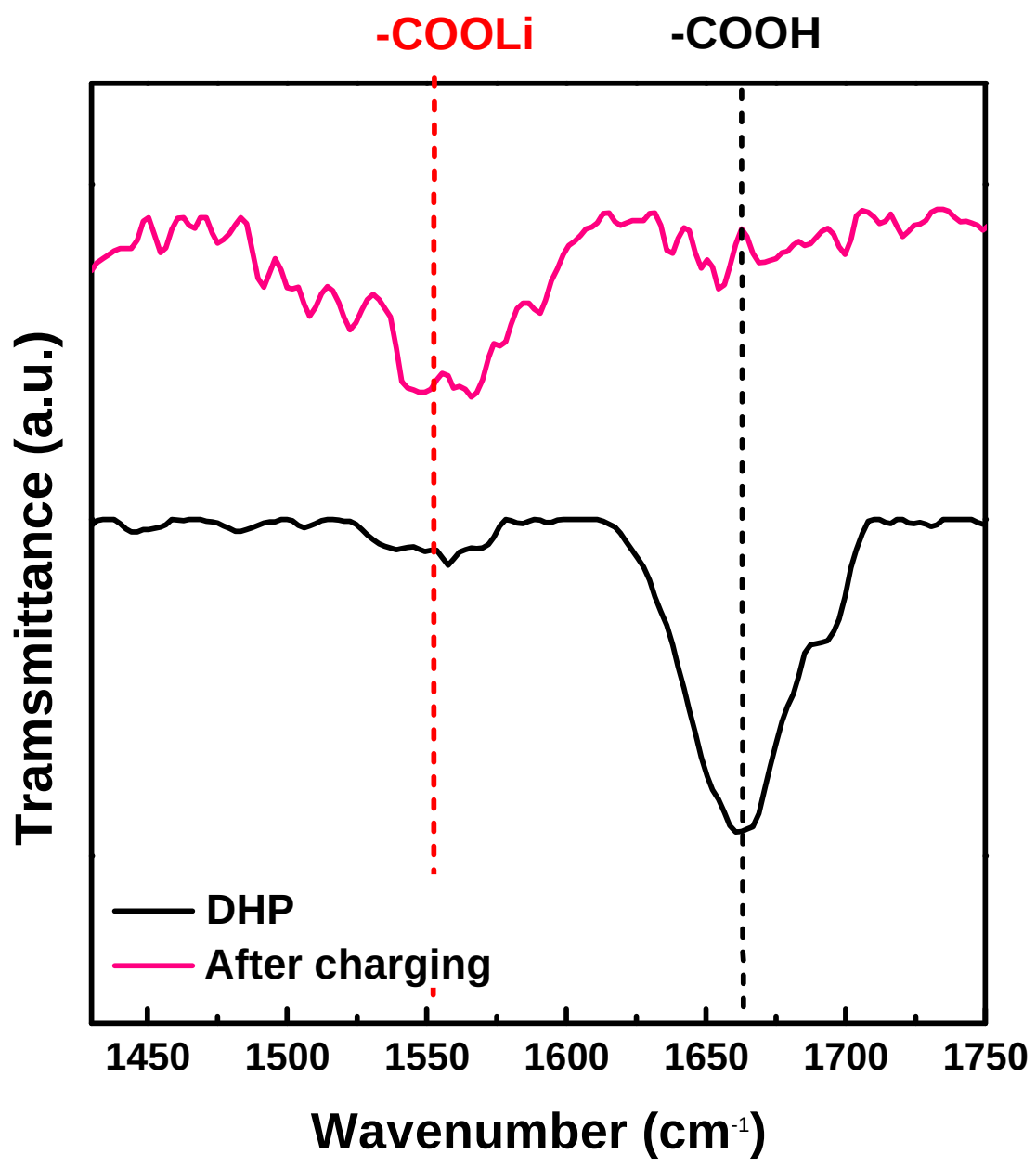

Fig. R1. FT-IR spectra of DHP and DHP electrodes after charging in the first cycle.

[R7] Yi, M. et al. In-situ coating and surface partial protonation co-promoting performance of single-crystal nickel-rich cathode in all-solid-state batteries, *J. Energy Chemistry* **89**, 137–143 (2024).

[R8] Yang, J. et al. Suppressing voltage fading of Li-rich oxide cathode via building a well-protected and partially-protonated surface by polyacrylic acid binder for cycle-stable Li-ion batteries, *Adv. Energy Mater.* **10**, 1904264 (2020).

[R9] Zhang, Q. et al. High-performance PEO-based solid-state $LiCoO_2$ lithium metal battery enabled by poly(acrylic acid) artificial cathode electrolyte interface, *Mater. Today Energy* **26**, 101128 (2022).

Correction in the revised manuscript

1) Correction in Results and Discussion section.

The Li 1s peak at 55 eV in the DHP electrode increased after activation, indicating the formation of -COO^-Li^+ rather than Li_2CO_3. The post-cycling FT-IR analysis further supports the XPS results, as shown in **Fig. S4**. The characteristic –COOH peak of PAA observed in the pristine DHP electrode disappears after the first charge and discharge process, while a distinct –COOLi peak emerges at around 1550 cm^{-1}. These results provide strong evidence for the formation of Li-PAA on the NCM surface during the first electrochemical cycle. In addition, the structural changes in NCM resulting from the activation process were investigated by the X-ray diffraction (XRD) analysis of the PVDF, SPDX, and DHP electrodes after activation.

2) Correction in Supporting information.

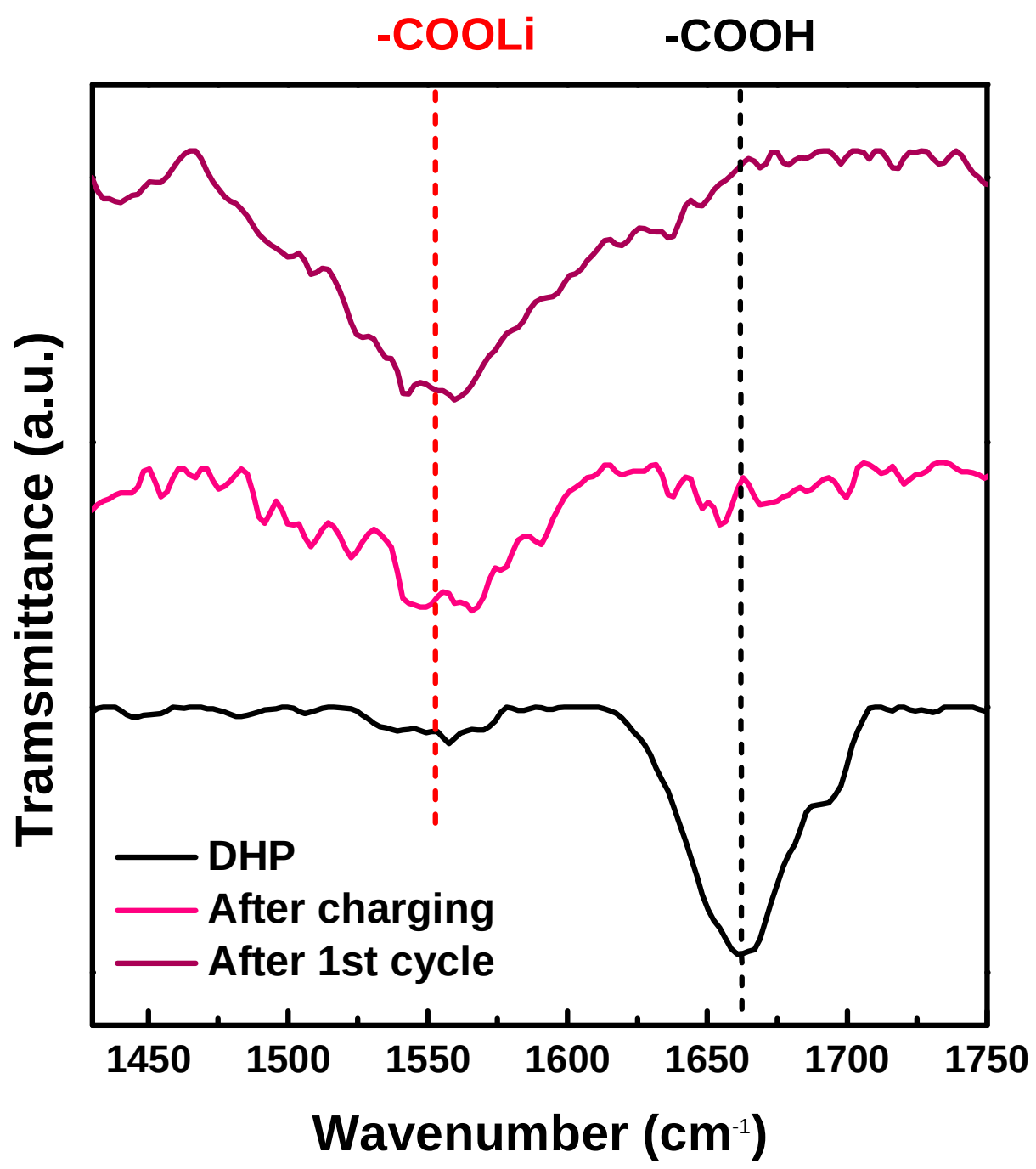

Fig. S4. FT-IR spectra of the pristine DHP electrode and DHP electrodes after charging and the first cycle.

Comment 5

The scale text in Figure 5g is too small and differs from that in other diagrams.

Our response

In accordance with the reviewer’s suggestion, we have revised the scale text in Fig. 5g. The corresponding modifications have been incorporated into the revised 'Results and Discussion' section, as provided below.

Correction in the revised manuscript

1) Correction in Results and Discussion section.

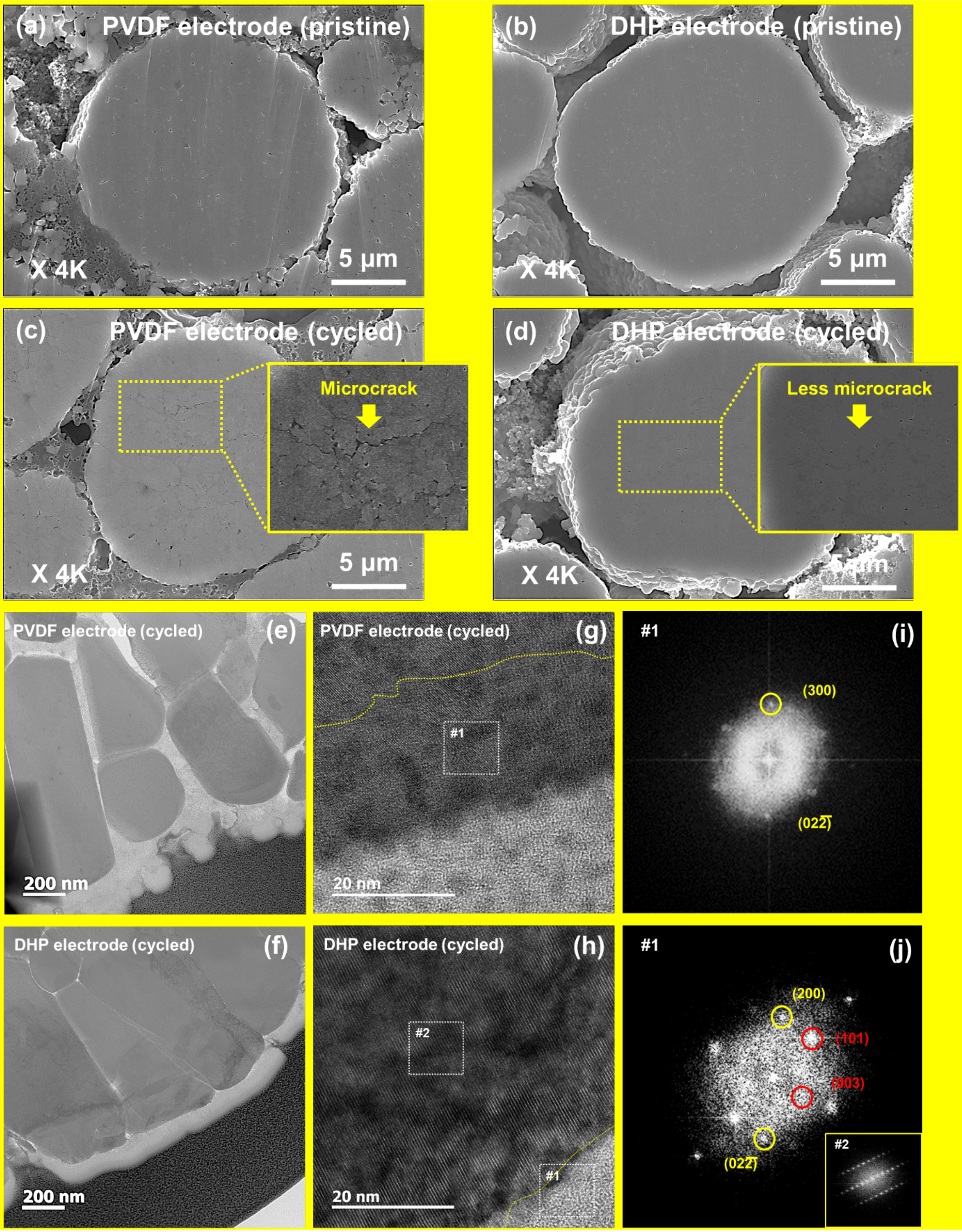

Fig. 5. Focused ion beam–scanning electron microscopy (FIB-SEM) images of the pristine Ni-rich cathodes using (a) PVDF and (b) DHP. FIB-SEM images of the cycled Ni-rich cathodes using (c) PVDF and (d) DHP. STEM images of the cycled NCM in (e) PVDF- and (f) DHP-based cathode. High-resolution transmission electron microscopy (HR-TEM) images of the

cycled NCM in (g) PVDF- and (h) DHP-based cathode. Corresponding fast Fourier transform (FFT) patterns of the cycled NCM in (i) PVDF- and (j) DHP-based cathode.

Responses to the comments by Reviewer #2:

This manuscript reports the development of an aqueous and dual-functional hybrid polymer (DHP) binder composed of spandex (SPDX) and poly(acrylic acid) (PAA), enabling the fabrication of ultra–high–mass–loading Ni-rich cathodes (up to 70 mg cm^{-2}) via a conventional wet process. The study demonstrates significantly improved adhesion strength, Li^+ transport kinetics, and structural integrity, resulting in outstanding areal capacities and an impressive pouch-cell energy density of 451 Wh kg^{-1}. Overall, the work presents a technically robust and industrially potential approach. However, several critical issues require clarification and additional validation before the manuscript can be considered for publication. The study would benefit from deeper mechanistic insights, more transparent data presentation, and further control experiments to reinforce the reliability of the conclusions.

Comment 1

The authors should clearly elucidate the distinct roles of PAA and SPDX within the DHP system and explain why each single-component binder fails to achieve comparable performance. It is strongly recommended to include electrochemical results for electrodes using only PAA and only SPDX as controls, to verify the claimed synergistic effect.

Our response

As the reviewer pointed out, to demonstrate the effectiveness of DHP, it is essential to show that each single-component binder fails to achieve comparable performance. We appreciate this

insightful suggestion and have further clarified the comparative results of the single-component binders. As shown in Fig. S3, due to the rigid and brittle nature of PAA, the PAA electrode exhibited surface cracking and weakened interparticle adhesion after drying. Such brittle mechanical properties are known to cause structural instability and pose challenges in preventing electrode cracking during cycling in NCM electrodes [R1-2]. In addition, since SPDX extensively covers the NCM particles, this conformal coverage may increase interfacial resistance and induce cell polarization, particularly in thick electrodes. Consistently, the SPDX electrode showed more severe polarization in the voltage profiles and faster capacity decay during cycling than the DHP electrode (Fig. S10 and S11). In this regard, DHP was designed by employing SPDX as the backbone structure to protect the electrode surface and enhance mechanical robustness, while PAA was incorporated as a complementary component at half the proportion of SPDX to improve Li^+ transport properties. Through this design, the limitations of each single-component binder were minimized while their respective advantages were maximized. To more clearly elucidate the influence of single-component binders on the electrochemical performance of NCM electrodes, cycling tests of Li half-cells with 20 mg cm^{-2} NCM cathodes were further conducted at 0.5 C. As shown in **Fig. R1**, after 100 cycles, DHP, SPDX, and PAA exhibited capacity retentions of 90.1%, 80.4%, and 70.5%, respectively. These results clearly demonstrate that neither PAA nor SPDX alone can achieve the cycling stability delivered by DHP. The inferior cycling performance of PAA electrode can be attributed to its intrinsic brittleness, whereas that of the SPDX electrode is associated with increased polarization arising from extensive surface coverage of NCM particles, which may hinder efficient Li^+ transport. In contrast, DHP achieves improved overall electrochemical stability through the synergistic integration of mechanical reinforcement (from SPDX) and enhanced ionic transport (from PAA), thereby enabling stable cycling even under relatively high mass-loading conditions.

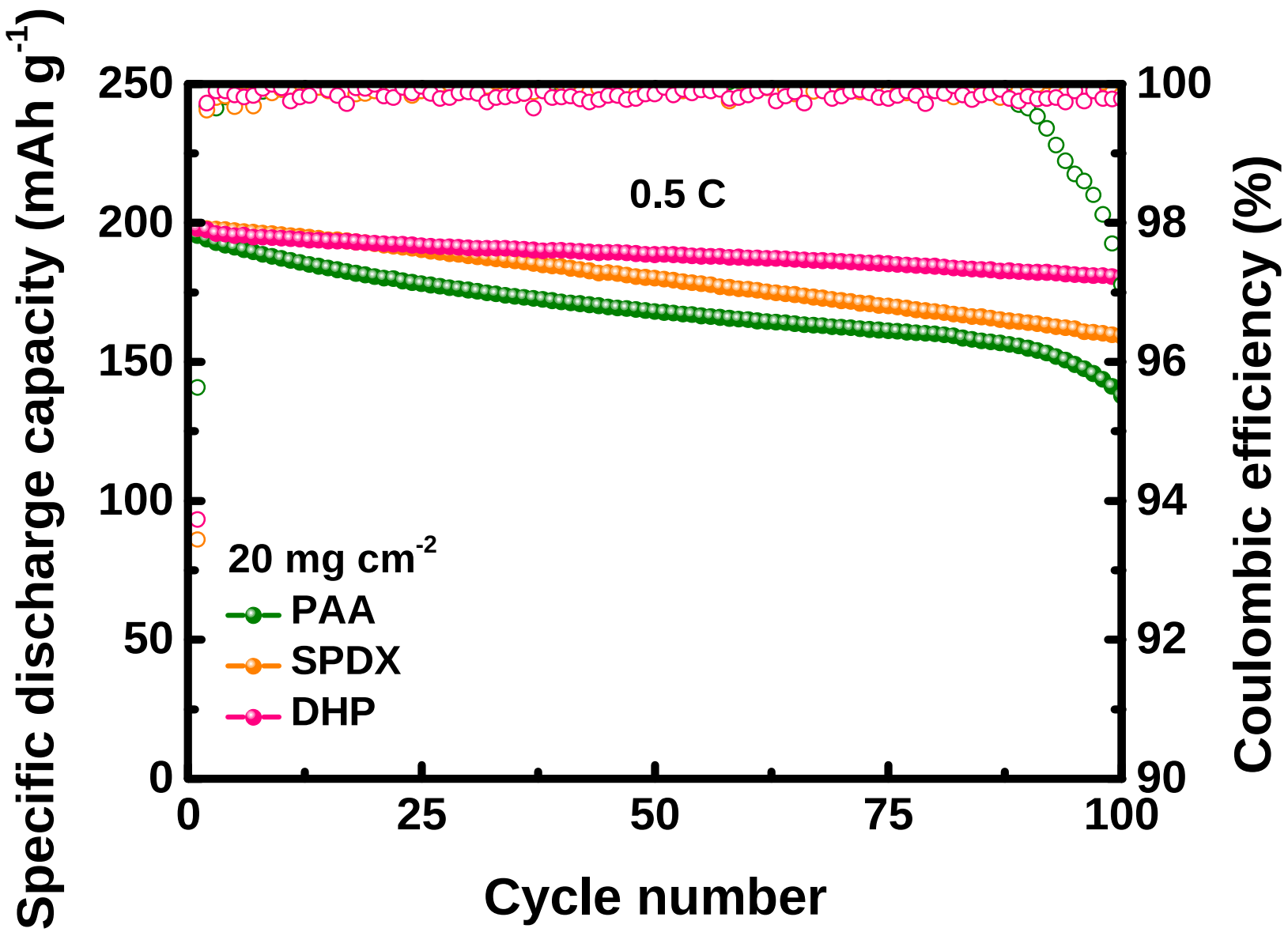

Fig. R1. Cycling performance of half cells employing different electrodes.

[R1] Pillai, A. M. et al. Aqueous binders for cathodes: A lodestar for greener lithium ion cells, *Energy & Fuels* **36**, 5063–5087 (2022).

[R2] Wang, H. et al. Sodium phytate cross-linked polyacrylic acid as multifunctional aqueous binder stabilizes LiNi0.8Co0.1Mn0.1O2 to 4.6 V, *ACS Energy Lett.* **10**, 136–144 (2025).

Comment 2

As TEM images serve as essential evidence supporting the homogeneous distribution of the binder, Figures 2(a) and 2(d) are too indistinct to substantiate this claim. The authors should provide higher-resolution TEM images or complementary microscopic characterization (e.g., AFM or STEM mapping) to strengthen the conclusion regarding uniform binder dispersion.

Our response

Thank you for your valuable comment. In response, additional TEM analysis was performed to verify the homogeneous distribution of the binder using higher-resolution TEM images. As shown in **Fig. R2**a, the PVDF electrode exhibits a relatively thick and non-uniform binder layer on the NCM surface, with a localized thickness of approximately 69 nm. In contrast, the DHP electrode shows a uniformly distributed and significantly thinner binder layer, with a thickness of approximately 6 nm (Fig. R2b). These results are consistent with the uniform and thin binder coverage of DHP described in our work, providing direct microscopic evidence for improved binder dispersion in the NCM electrode. In addition, we have revised the TEM images in Fig. 2 to enhance clarity and resolution, as provided below.

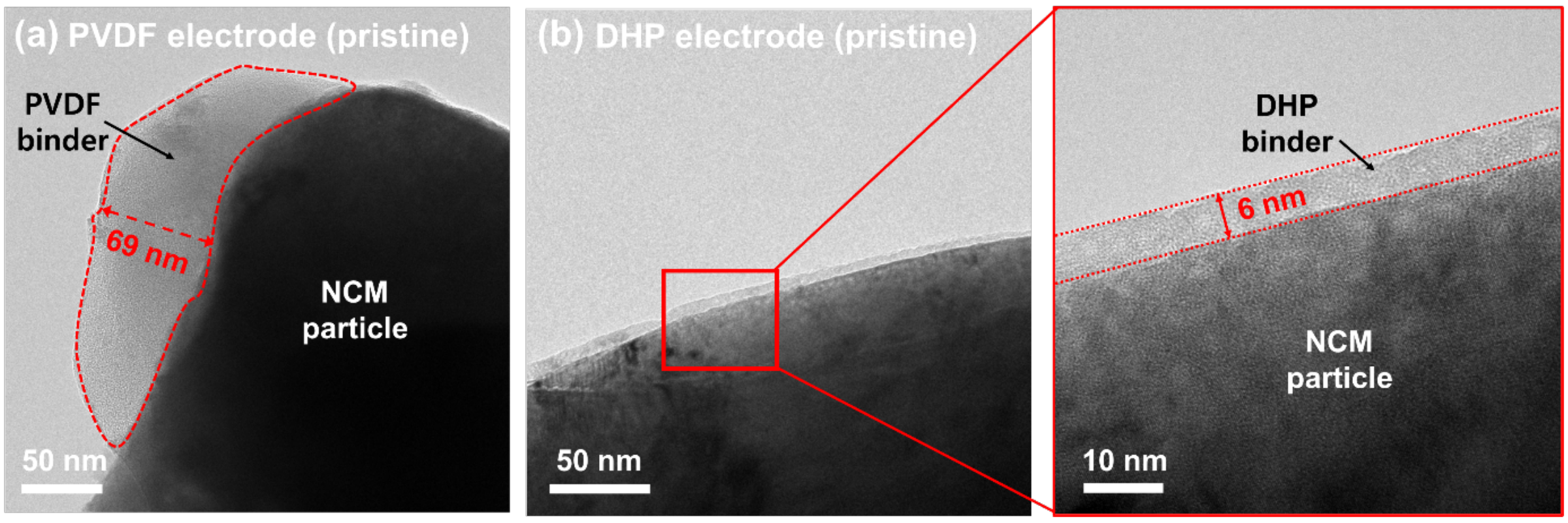

Fig. R2. High-resolution transmission electron microscopy (HR-TEM) images of the binder coverage on NCM particles in pristine electrodes using (a) PVDF and (d) DHP.

Correction in the revised manuscript

1) Correction in Results and Discussion section.

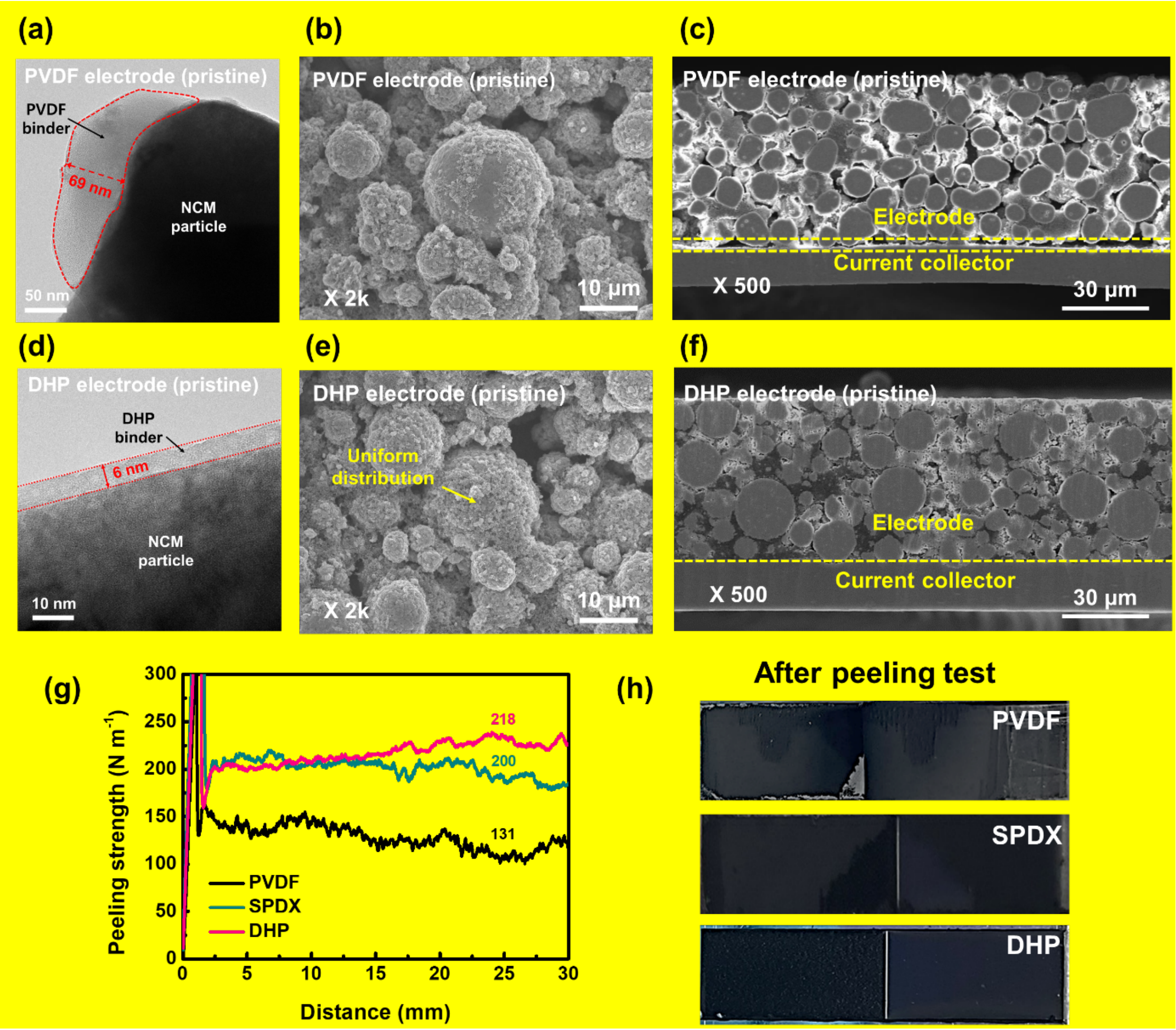

Fig. 2. Transmission electron microscopy (TEM) images of the binder coverage on NCM particles in pristine electrodes using (a) PVDF and (d) DHP. Top-view SEM images of pristine electrodes using (b) PVDF and (e) DHP. Cross-sectional SEM images of the pristine electrodes using (c) PVDF and (f) DHP. (g) 180° peeling test curves of the PVDF-, SPDX- and DHP-based electrodes. (h) Photographs after the peel test of the PVDF, SPDX and DHP electrodes.

Comment 3

Although the authors propose that Li–PAA is formed at the interface based on XPS and CV analyses, these are indirect indicators. The manuscript would be substantially strengthened by post-cycling FTIR or solid-state NMR studies, which can directly confirm the chemical

bonding evolution and validate the presence of Li–PAA.

Our response

Thank you for your valuable comment. In accordance with the reviewer's suggestion, post-cycling FT-IR analysis was performed on the DHP electrode after initial charging and after the first cycle to verify the evolution of chemical bonding and the formation of Li–PAA. As shown in **Fig. R3**, the pristine DHP electrode exhibits a –COOH peak originating from PAA. This peak disappears after the initial charging process, accompanied by the formation of a –COOLi peak at around 1550 cm^{-1}. In addition, after the first cycle, the –COOLi peak intensity further increases compared to that observed after charging. The disappearance of the –COOH signal and the concurrent emergence and growth of the –COOLi peak provide direct spectroscopic evidence for the formation of Li–PAA during the first activation process. Accordingly, the post-cycling FT-IR results have been added to the revised manuscript and Supporting information, as shown below.

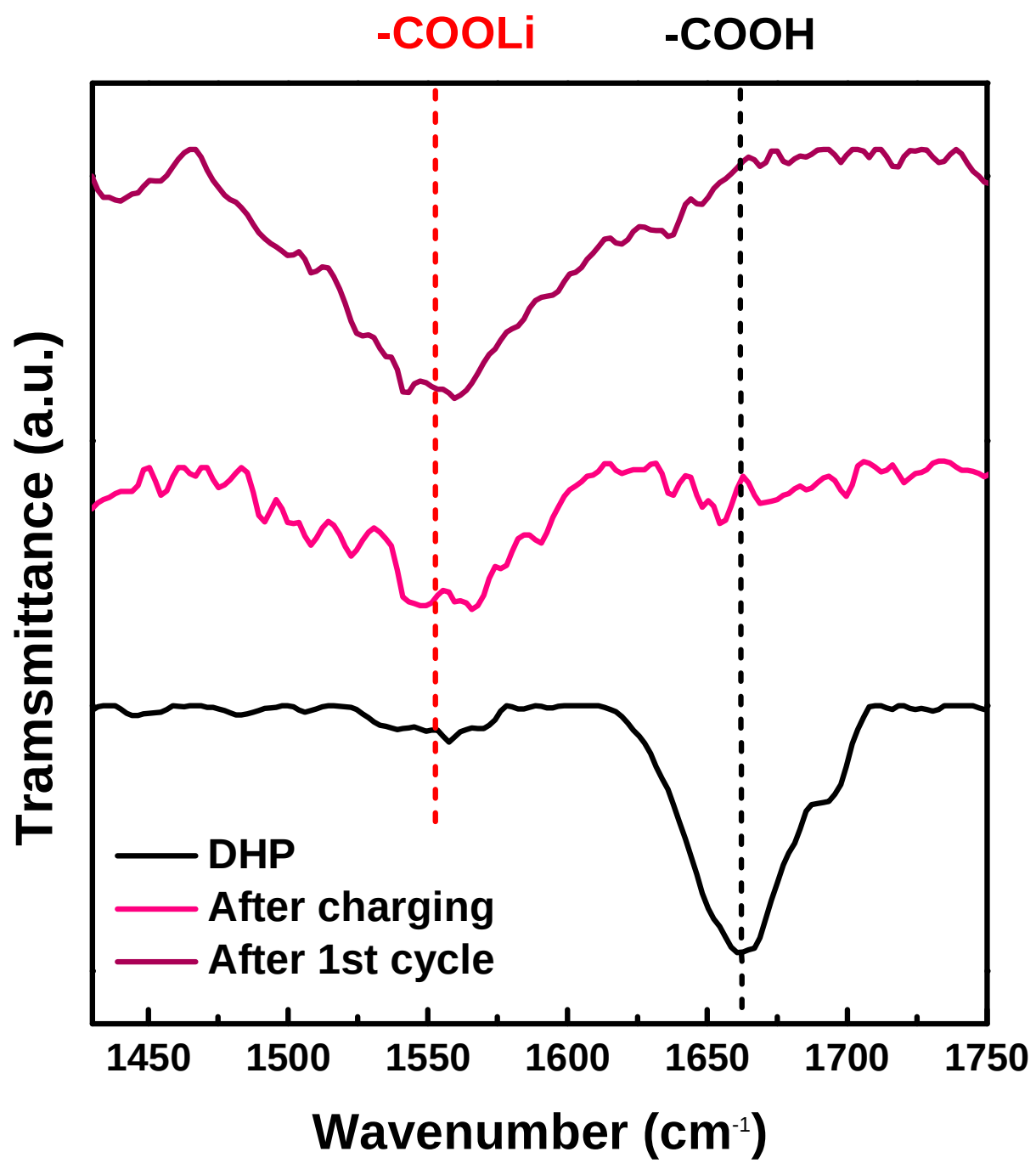

Fig. R3. FT-IR spectra of DHP and DHP electrodes after charging and the first cycle.

Correction in the revised manuscript

1) Correction in Results and Discussion section.

The Li 1s peak at 55 eV in the DHP electrode increased after activation, indicating the formation of -COO^-Li^+ rather than Li_2CO_3. The post-cycling FT-IR analysis further supports the XPS results, as shown in **Fig. S4**. The characteristic –COOH peak of PAA observed in the pristine DHP electrode disappears after the first charge and discharge process, while a distinct –COOLi peak emerges at around 1550 cm^{-1}. These results provide strong evidence for the formation of Li-PAA on the NCM surface during the first electrochemical cycle. In addition, the structural changes in NCM resulting from the activation process were investigated by the X-ray diffraction (XRD) analysis of the PVDF, SPDX, and DHP electrodes after activation.

2) Correction in Supporting information.

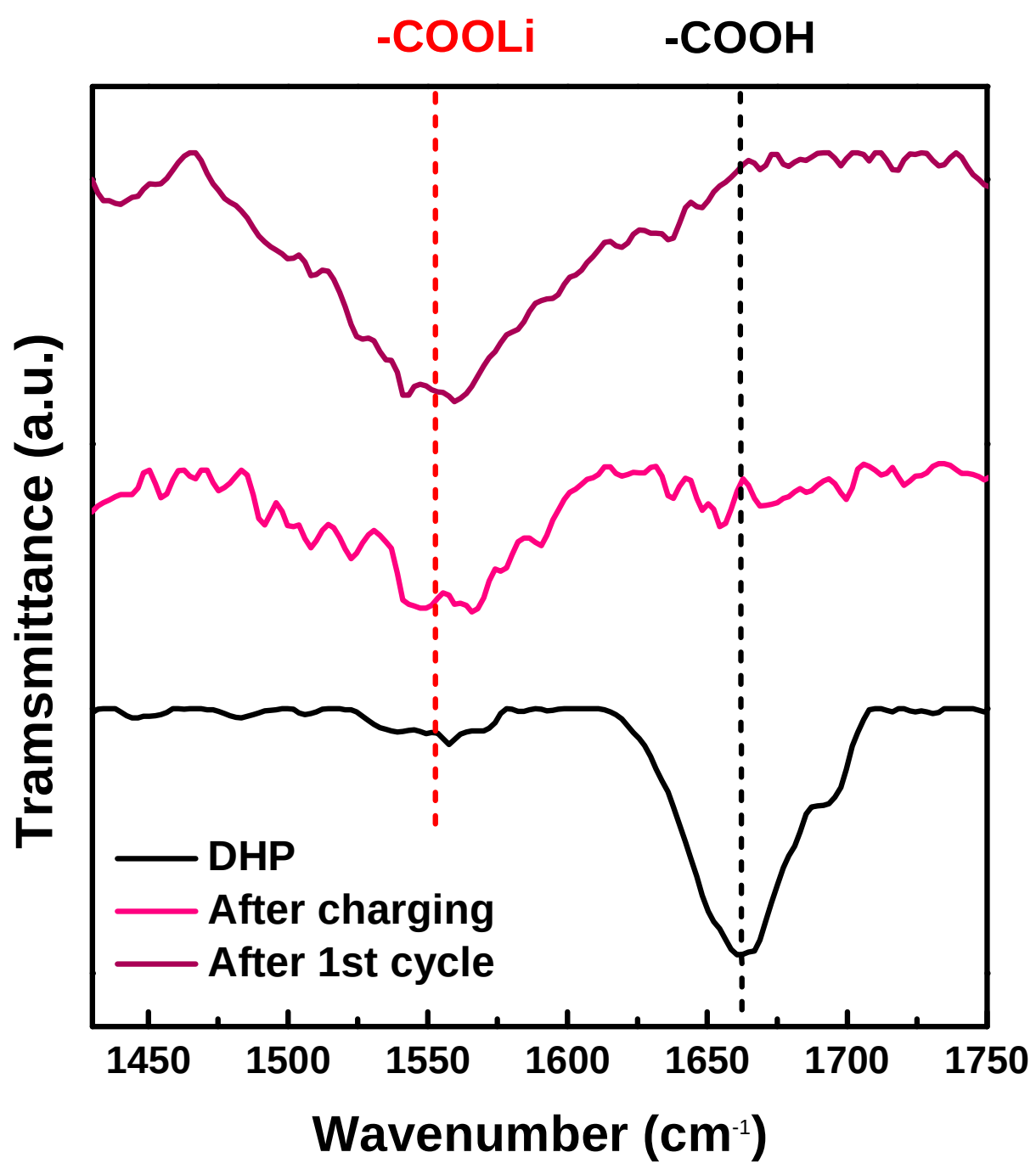

Fig. S4. FT-IR spectra of the pristine DHP electrode and DHP electrodes after charging and the first cycle.

Comment 4

The interpretation of the Li_2CO_3 signal as originating from a “native CEI layer” (line 283) is not sufficiently justified. It is more plausible that the signal arises from residual alkaline species remaining on the NCM particle surface after synthesis. Therefore, the Li–PAA formation should be reinterpreted as an interfacial reaction between PAA and these surface residues, rather than with a pre-formed CEI layer.

Our response

We greatly appreciate your valuable comments on our work. As the reviewer pointed out, the

Li_2CO_3 species observed in the surface layer of pristine electrode should be more precisely interpreted as a residual lithium-containing alkaline species remaining on the NCM particle surface after synthesis. In the original manuscript, we intended to indicate that the surface layer observed in the pristine electrode was a naturally formed layer, distinct from the Li-PAA layer. To clarify this point and avoid potential misunderstanding, we have revised the relevant description in the Results and Discussion section of the manuscript, as shown below.

However, based on the combined electrochemical and spectroscopic evidence, the formation of Li–PAA is primarily attributed to the H^+/Li^+ exchange reaction occurring during the first electrochemical cycle, rather than to a direct interfacial reaction between PAA and pre-existing surface residual species. This interpretation is supported by the distinct electrochemical behavior of the DHP electrode observed in the CV results, as well as by the formation of Li-PAA after the first cycle in the XPS analysis. Additionally, post-cycling FT-IR analysis further corroborates these findings. As shown in **Fig. R4**, the pristine DHP electrode exhibits a –COOH peak originating from PAA. This peak disappears after the initial charging process, accompanied by the formation of a –COOLi peak at around 1550 cm^{-1}. In addition, after the first cycle, the –COOLi peak intensity increases compared to that observed after charging. The disappearance of the –COOH signal and the concurrent emergence and growth of the –COOLi peak provide direct spectroscopic evidence for the formation of Li–PAA during the first activation process. Collectively, these results support the conclusion that Li–PAA formation predominantly occurs during the first electrochemical activation cycle. We once again appreciate the reviewer's valuable comments, which have contributed to improving the quality of our work.

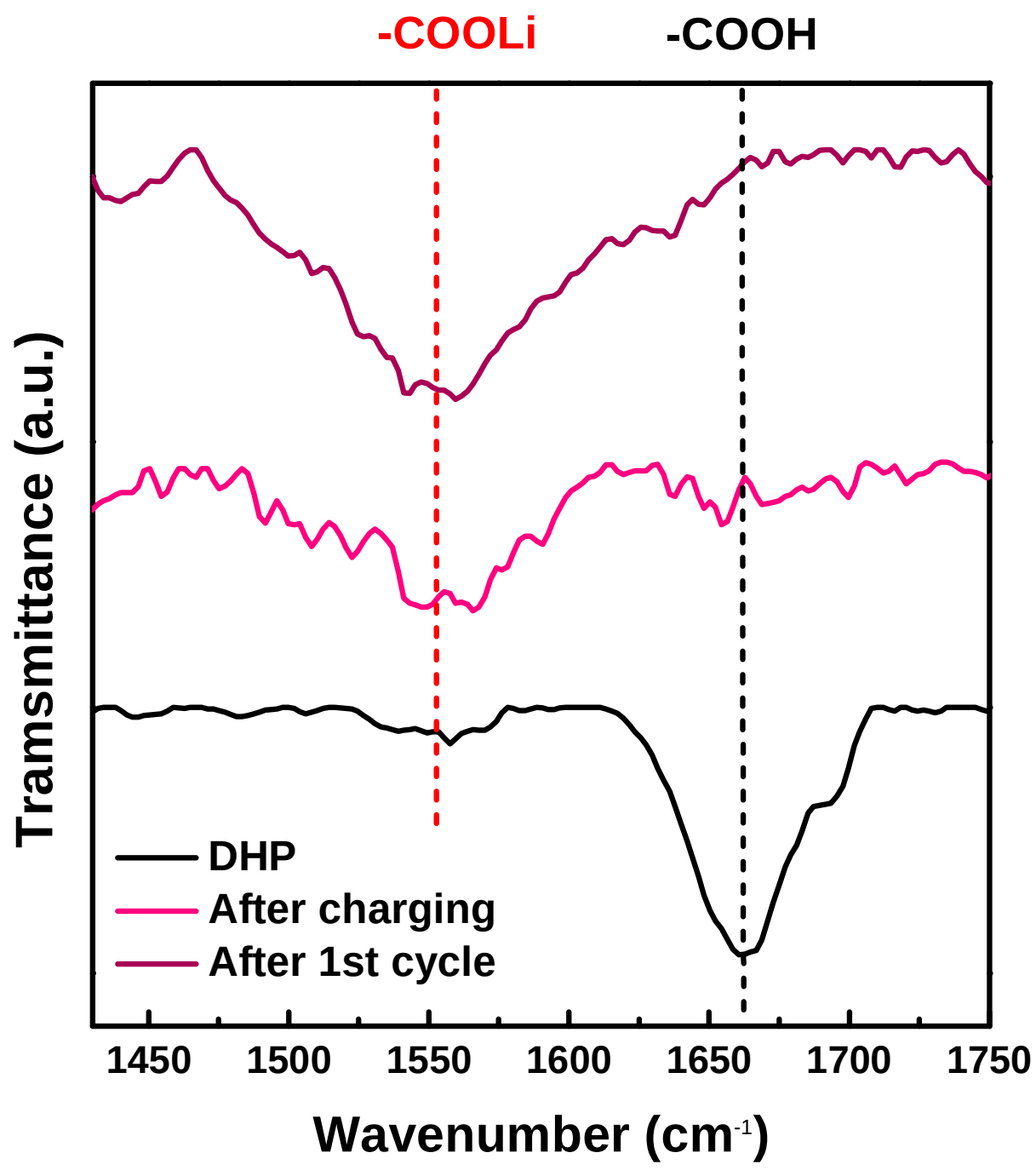

Fig. R4. FT-IR spectra of DHP and DHP electrodes after charging and the first cycle.

Correction in the revised manuscript

1) Correction in Results and Discussion section.

Fig. 3e and f present the Li 1s XPS profiles of PVDF before and after the first cycle, and Fig. 3g and h exhibit the Li 1s peaks of DHP before and after the first cycle. A peak corresponding to Li_2CO_3 was observed for both the PVDF and DHP electrodes before cycling, likely due to the residual lithium-containing alkaline species remaining on the NCM surface after synthesis. After the activation cycle at 0.1 C, the Li 1s peak at 55 eV in the PVDF electrode did not change significantly (Fig. 3e, f).

Comment 5

Since peeling strength is inherently dependent on the specimen width, the adhesion results in Figure 2(g) should be normalized by width and expressed in N m^{-1}, to ensure quantitative consistency and fair comparison among different samples.

Our response

Thank you for your valuable comments. We performed the peeling tests using electrode samples with a fixed width of 1 cm. As suggested by the reviewer, to ensure quantitative consistency and enable fair comparison, we normalized the peeling strength values by specimen width and expressed them in units of N m^{-1} in Fig. 2g and Fig. S12. In addition, detailed information regarding the specimen dimensions and normalization procedure has been added to the Experimental section. The revised Results and Discussion, Experimental Section, and Supporting Information have been modified accordingly, as provided below.

Correction in the revised manuscript

1) Correction in Results and Discussion section.

The DHP electrode exhibited the highest average peeling strength (218 N m^{-1}), nearly twice that of the PVDF electrode (131 N m^{-1}), and even higher than that of the SPDX electrode (200 N m^{-1}), which is well known for its strong adhesion.

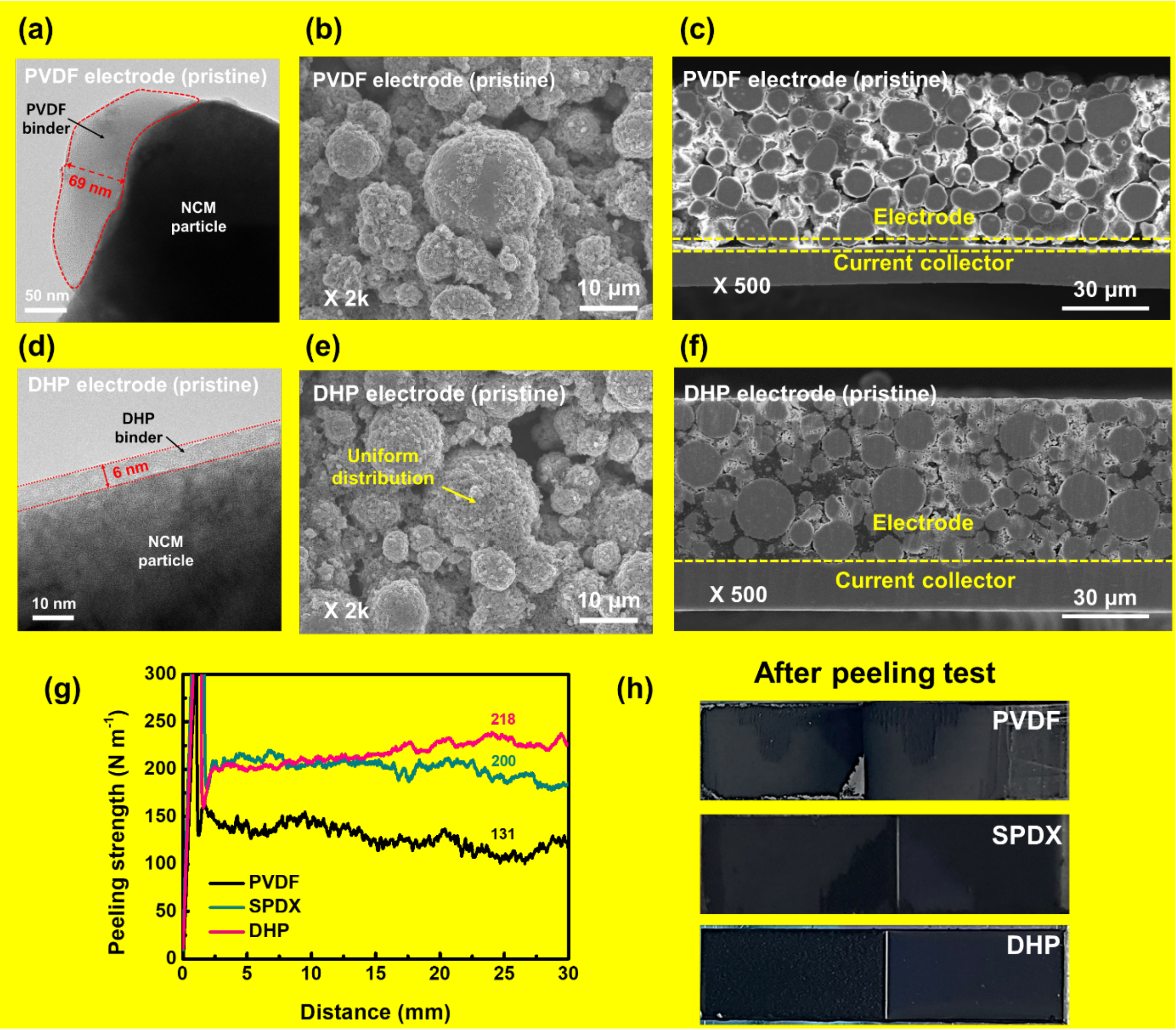

Fig. 2. Transmission electron microscopy (TEM) images of the binder coverage on NCM particles in pristine electrodes using (a) PVDF and (d) DHP. Top-view SEM images of pristine electrodes using (b) PVDF and (e) DHP. Cross-sectional SEM images of the pristine electrodes using (c) PVDF and (f) DHP. (g) 180° peeling test curves of the PVDF-, SPDX- and DHP-based electrodes. (h) Photographs after the peel test of the PVDF, SPDX and DHP electrodes.

2) Correction in Supporting information.

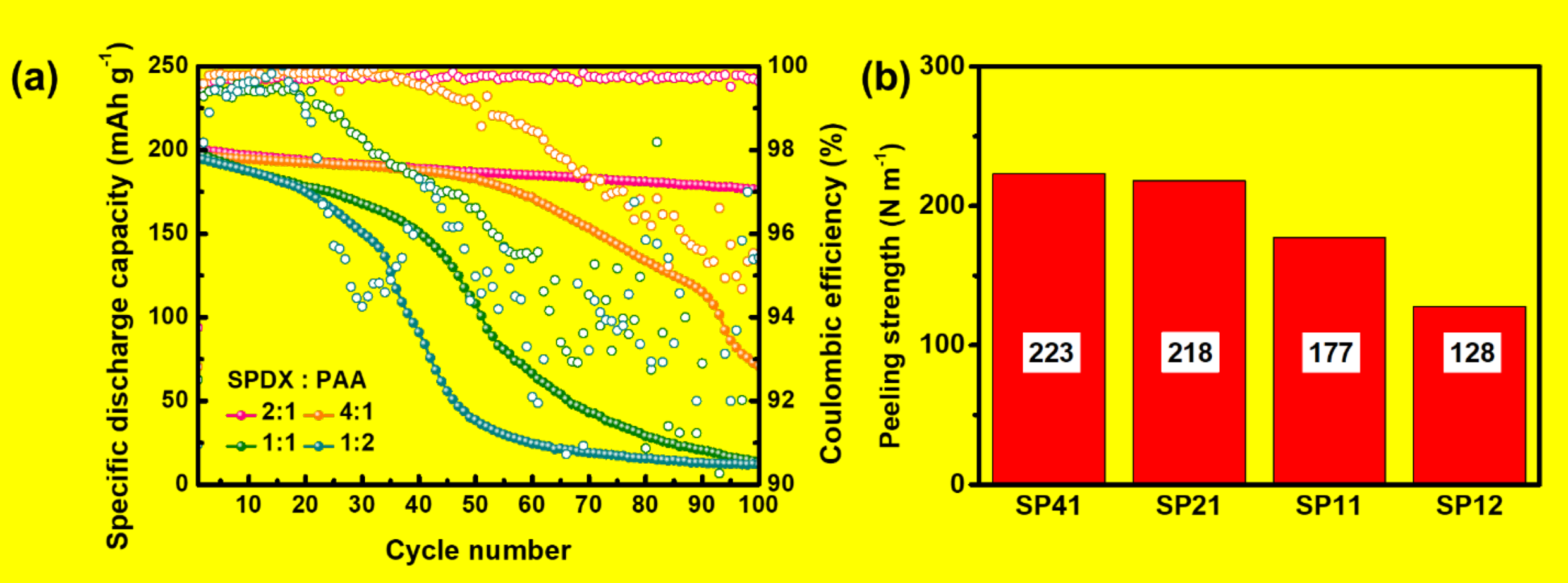

Fig. S14. (a) Cycle performances and (b) 180° peeling tests of DHP electrodes prepared using various combinations of SPDX and PAA binders.

3) Correction in Experimental section.

Characterization: Mechanical characteristics of the electrodes were assessed using a universal testing machine (UTM, JSV-H1000), including 180° peel tests using 3M double-sided tape at a peel rate of 25 mm min^{-1}. All samples used for the peel tests were prepared with a fixed width of 1 cm, and peeling strength normalized by sample width (N m^{-1}). FT-IR spectroscopy (TEMSOR27, Bruker, Germany) was employed to investigate the polymeric bonding structures. Electrode morphology was observed through FE-SEM (SU8010, HITACHI) and cross-sectional imaging via FIB (Helios 650, FEI, USA).

Comment 6

To facilitate a more convincing comparison of CEI compositions, the authors are encouraged to provide fitted peak areas and/or atomic ratios derived from XPS spectra. Such quantitative data would enhance the credibility and clarity of the compositional analysis.

Our response

We greatly appreciate your valuable comments on our work. In response to the reviewer's comments, the peak area ratios for each XPS spectrum shown in Fig. 6 have been added to the Supporting Information. The revised Supporting Information is provided below.

Correction in the revised manuscript

1) Correction in Results and Discussion section.

To further confirm the inhibitory effect of DHP on the surface degradation of the NCM cathode, XPS analysis was conducted on the surface of the NCM cathode after cycling, as shown in **Fig. 6**a–f and **S8**. In the C 1s spectra, both the PVDF and DHP electrodes showed a C-O peak at 286.3 eV, associated with the decomposition of the carbonate-based electrolyte, with the PVDF electrodes displaying a notably higher peak intensity (Fig. 6a,d).

2) Correction in Supporting information

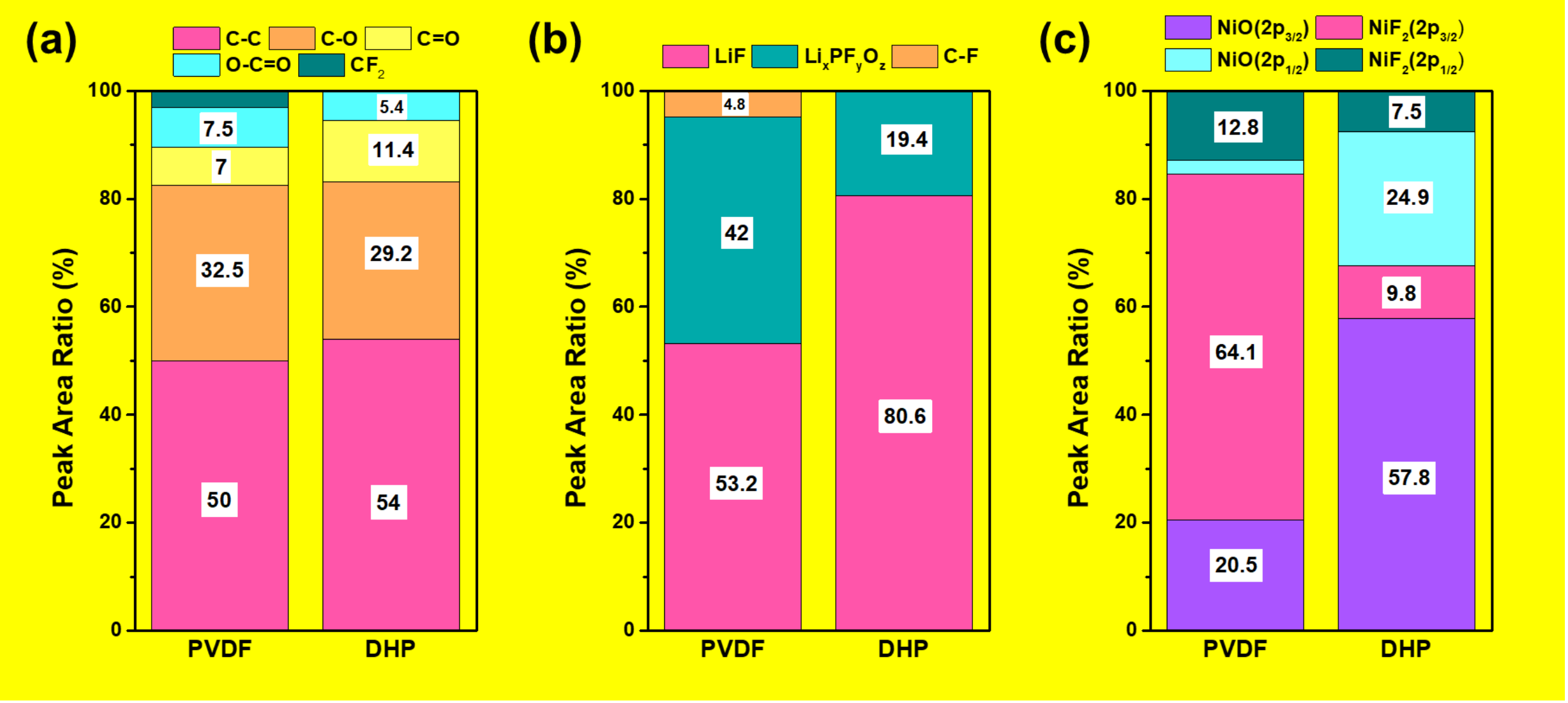

Fig. S8. Peak area ratios obtained from the XPS spectra of (a) C 1s, (b) F 1s, and (c) Ni 2p.

Comment 7

The cycling performance of the 70 mg cm^{-2} electrode, limited to only 25–50 cycles, is insufficient to substantiate the claimed long-term stability. The authors should extend the cycling test to at least 200 cycles or include post-mortem structural characterization to confirm durability. Additionally, to eliminate possible artifacts from Li-metal degradation, full-cell tests using a more stable counter electrode (e.g., Si-based or graphite anode) are strongly recommended.

Our response

We greatly appreciate your valuable comments on our work. As the reviewer pointed out, full-cell evaluation using a 70 mg cm^{-2} electrode would be valuable to eliminate potential artifacts arising from Li-metal degradation and to further validate long-term stability. However, constructing a stable full-cell system at such an ultra-high areal capacity (15 mAh cm^{-2} for the cathode) requires a matching anode with an areal capacity exceeding 17 mAh cm^{-2}. At this level of areal capacity, both silicon- and graphite-based anodes are known to encounter severe kinetic and mechanical limitations, including non-uniform ion transport, localized volume expansion, increased overpotentials, and Li plating, which significantly compromise long-term cycling stability [R3-6]. While we acknowledge the importance of full-cell validation, the optimization of such high-areal-capacity anodes remains a substantial technical challenge beyond the scope of the present revision. We sincerely apologize for this limitation and will address this issue in future work. To instead evaluate whether the observed capacity fading originates from NCM degradation, post-mortem analyses were performed on the 70 mg cm^{-2} DHP electrode after 50 cycles. The cycled DHP electrode was reassembled with fresh Li metal and electrolyte, followed by one formation cycle at 0.1 C and subsequent charge–discharge

testing at 0.2 C. As shown in **Fig. R5**, the reassembled cell retained 97.1% of its original capacity (14.0 mAh vs. 13.6 mAh), despite slightly increased polarization. This result indicates that the structural and electrochemical integrity of the NCM cathode is largely preserved after 50 cycles, suggesting that the limited cycle life is not primarily caused by severe cathode degradation. In addition, SEM analysis of the reassembled DHP electrode (**Fig. R6**a–b) reveals no significant particle fracture or crack formation after 50 cycles. These structural observations are consistent with the electrochemical recovery test and further support the conclusion that the limited cycle life of the 70 mg cm^{-2} DHP electrode is not primarily attributed to degradation of the NCM particles.

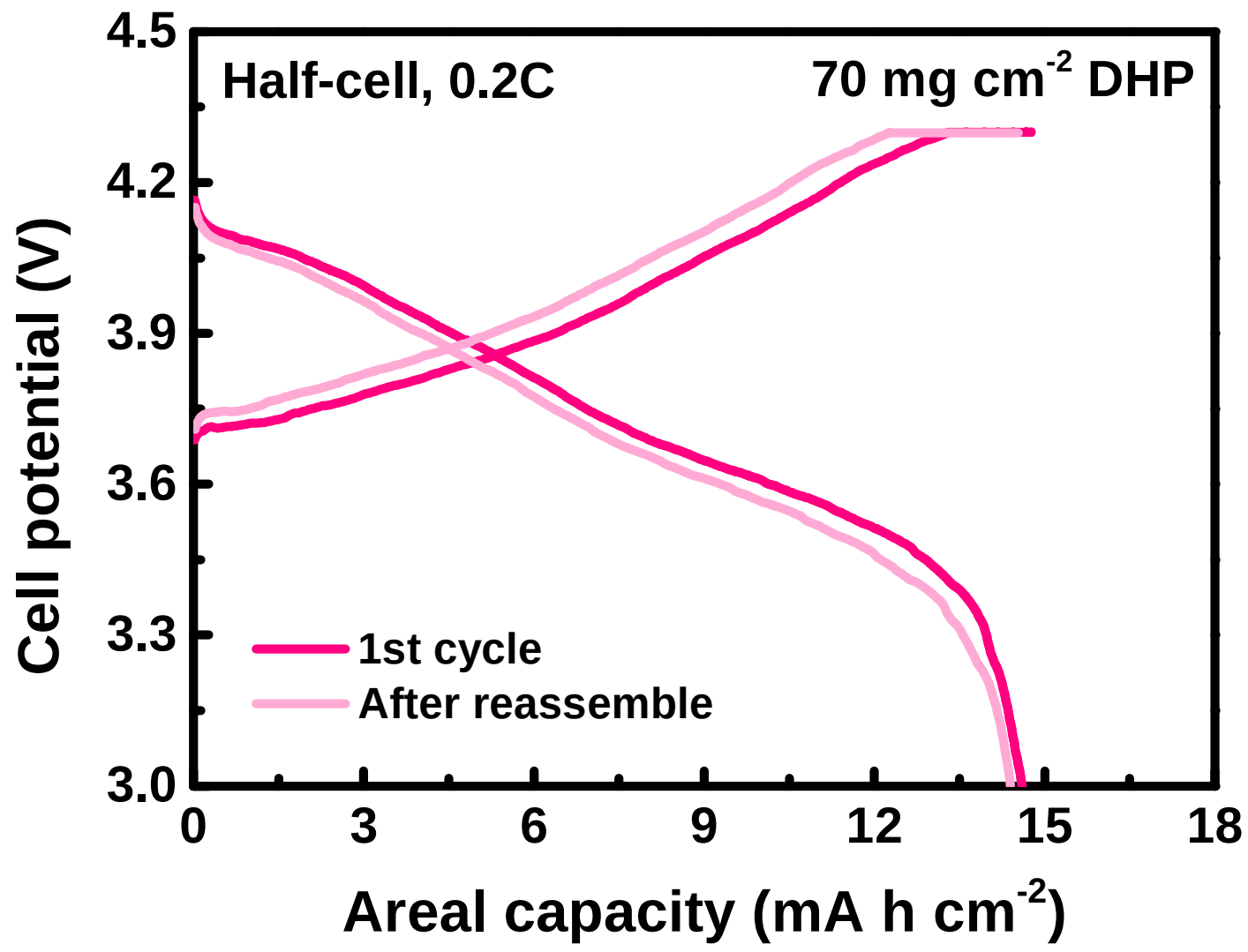

Fig. R5. Galvanostatic Voltage profile of pristine and reassembled 70 mg cm^{-2} DHP electrodes under C-rate of 0.2 C

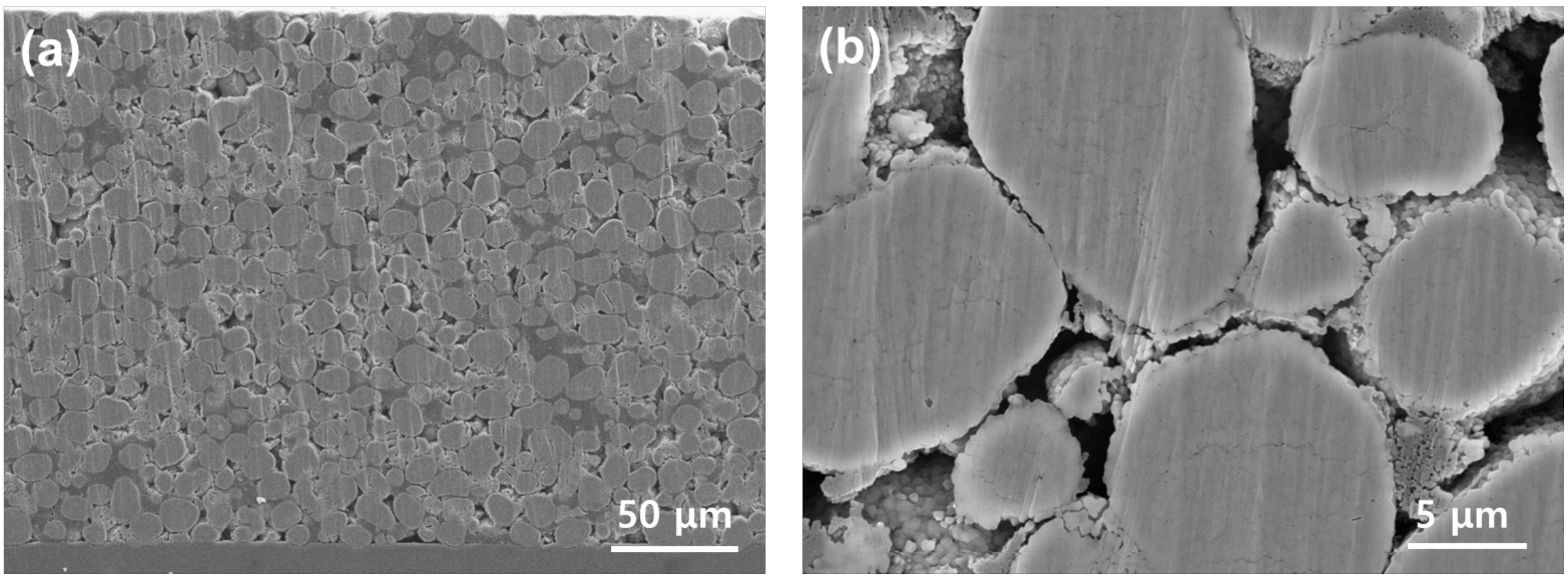

Fig. R6. SEM images of 70 mg cm^{-2} DHP electrodes after 50 cycles.

[R3] Thapa, A. & Gao, H. High areal loading silicon nanoparticle-based lithium-ion batteries, *ACS Appl. Mater. Interfaces* **17**, 9219–9230 (2025).

[R4] Geng, S. et al. Impact of thickness and charge rate on the electrochemical performance of Si-based electrodes, *Cell Rep. Phys. Sci.* **5**, 102305 (2024).

[R5] Yoon, J. et al. Deciphering the degradation mechanism of thick graphite anodes in high-energy-density Li-ion batteries by electrochemical impedance spectroscopy, *J. Ind. Eng. Chem.* **138**, 424–431 (2024).

[R6] Ko, K.-H. et al. Degradation mechanism induced by depth-dependent inhomogeneity in thick high-areal-capacity graphite electrode, *Small* **21**, 2410795 (2025).

Responses to the comments by Reviewer #3:

In this article the authors report a new promising binder allowing the wet-processing of very high areal capacity positive electrodes, which would allow the obtaining of lithium-ion

batteries with higher energy density, which is sought by the battery industry. This new binder also shows promising properties allowing for a longer cyclability of Ni-rich cathode material by helping better cycling stability of the structure of such cathode material. Overall the results presented by the authors are making sense. However, for convincing the reader some complementary data or experiments are required as below:

Comment 1

In Figure 1b, the PAA spectrum is unexpected, as the characteristic bands that include broad O-H stretching in the 3500 to 2500 cm-1, and CH2 asymmetric and symmetric stretching at 2950 and 2800 cm-1 are strikingly lacking.

Our response

Thank you for your valuable comments. As the reviewer pointed out, characteristic bands of PAA, including O–H stretching and CH_2 asymmetric and symmetric stretching modes, are not clearly distinguishable in the original scale of Fig. 1b. However, this does not indicate the absence of these peaks, as their relatively low intensities compared to other bands and the scaling of the spectrum make them less apparent in the figure. In the enlarged and rescaled FT-IR spectrum of the PAA film (**Fig. R1**), the O–H stretching band in the range of 3500–2500 cm^{-1}, as well as the CH_2 asymmetric and symmetric stretching bands at approximately 2950 and 2800 cm^{-1}, can be clearly identified.

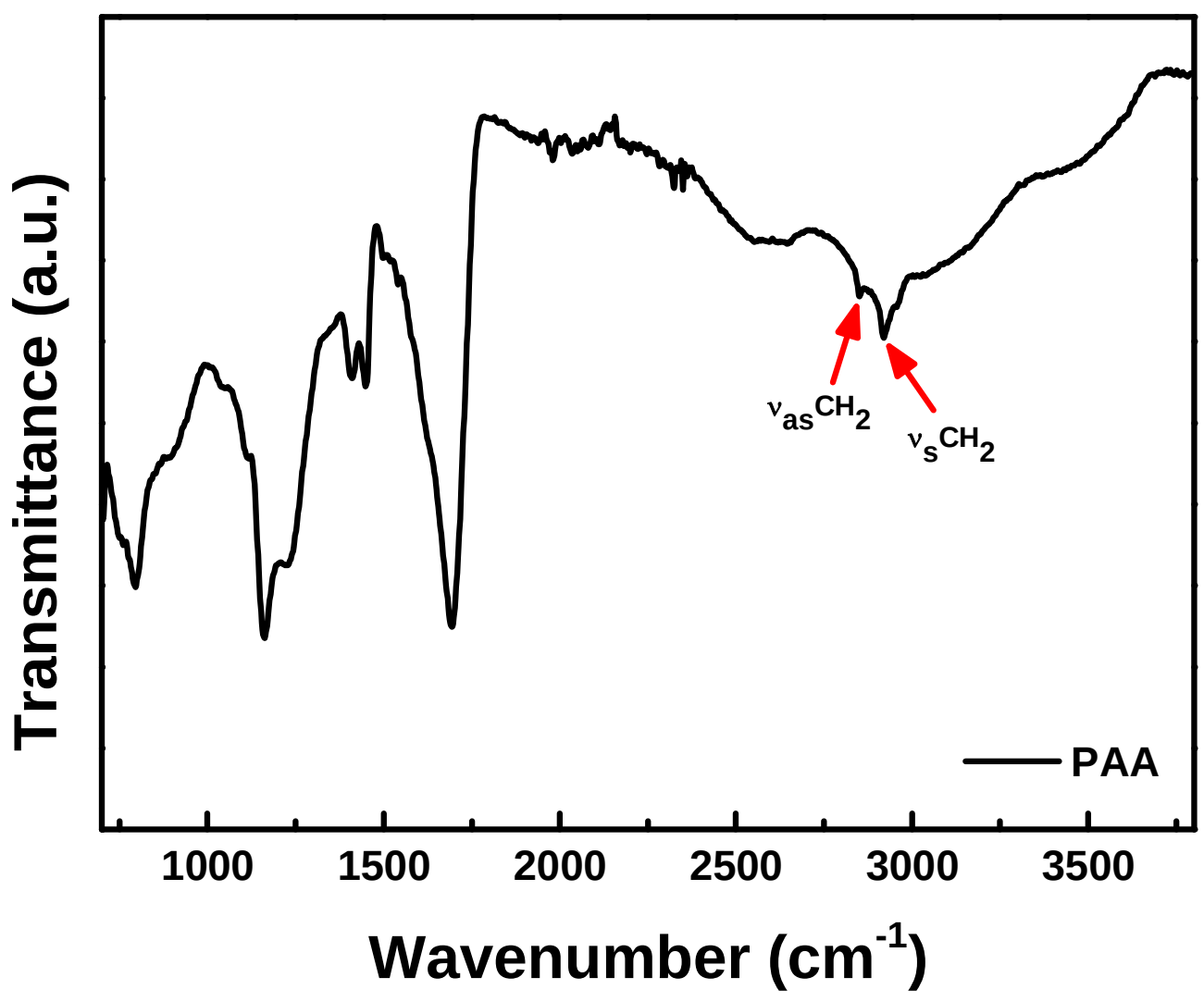

Fig. R1. Fourier transform infrared (FTIR) spectroscopy of PAA.

Comment 2

The description of the preparation of both positive and negative electrodes is far from being complete: protocole (steps, duration of each setp, equipment used for each step). All the important experimental details necessary to reproduce the work must be given (dry solid content of the slurries, doctor blade gap, SPDX provider and SPDX characteristics such as molar mass, Graphite powder & CMC & SBR providers and characteristics, current collectors thicknesses).

Our response

Thank you for your valuable comment. According to the reviewer's comment, we have added detailed descriptions of the experimental methods and materials sources to the 'Experimental' section of the revised manuscript, as shown below.

Correction in the revised manuscript

1) Correction in Experimental section.

Preparation of Electrodes and Cells: DHP-based electrodes were prepared by dispersing $LiNi_{0.8}Co_{0.1}Mn_{0.1}$ (NCM811, South Korea, D50 = 10 µm), Super P (Timcal, Switzerland), and the DHP binder in NMP (Junsei Chemical, Japan) at a weight ratio of 95:3:2. The resulting slurry was applied to an aluminum foil current collector using the doctor blade technique, dried under vacuum at 80 °C for 6 h, roll-pressed to a composite density of 3.3–3.4 g cm^{-3}, and then dried overnight at 100 °C. Control electrodes using PVDF (Sigma Aldrich, Mw ≈ 534,000), PAA, and SPDX binders were fabricated similarly. The active material loadings were set to 20, 30, 45, and 70 mg cm^{-2} for both DHP- and PVDF-based samples, corresponding to doctor blade gaps of 220, 350, 600, and 850 µm, respectively. Graphite electrodes were fabricated by slurry casting method. The slurry was prepared by dispersing graphite powder (BTR, S360-L2-H), carboxymethylcellulose (CMC, Sigma Aldrich, Mw ≈ 250 000), and Polymerized Styrene Butadiene Rubber (SBR, MTI, Mw ≈ 534,000) binder at a ratio of 97:1.5:1.5 in deionized water. The prepared slurry was cast onto Cu foil (11 µm). The obtained film was dried under vacuum at 100 °C for 6 h, roll-pressed to a composite density of 1.5 g cm^{-3}, and then dried overnight at 100 °C. Coin-type CR2032 cells were assembled in an argon-filled glovebox. A polyethylene (PE) separator (W-scope, 16 µm thickness) and an electrolyte consisting of 1 M $LiPF_6$ in EC/DEC (3:7 v/v) with 10 wt% FEC (PANAX ETEC) were used.

Comment 3

The description of the pouch cells is also laconic, as well as the samples preparations for

characterization techniques, in particular port-mortem ones. More information must be given.

Our response

According to the reviewer's comment, detailed descriptions of the pouch cells and the samples preparations for characterization techniques have been added to the 'Experimental' section of the revised manuscript, as shown below.

Correction in the revised manuscript

1) Correction in Experimental section.

A polyethylene (PE) separator (W-scope, 16 μm thickness) and an electrolyte consisting of 1 M $LiPF_6$ in EC/DEC (3:7 v/v) with 10 wt% FEC (PANAX ETEC) were used. For the preparation of NCM/Gr pouch full cells, the NCM cathode was cut to dimensions of 40 mm × 50 mm, and the Gr anode was cut to 45 mm × 55 mm. The E/C and N/P ratio of pouch cells were set to 2.0 g Ah^{-1} and 1.05, respectively, while the electrolyte and operating temperature were the same as those used in the coin cell evaluation. Pouch cells were assembled in dry room with a dew point of -50 °C.

Raman spectroscopy (LabRamn Soleil, Horiba) analysis was conducted using a 532 nm laser over a wavenumber range of 350–650 cm^{-1} with a resolution of 2 cm^{-1}. For post-mortem analyses (XRD, XPS, SEM, TEM, and Raman), the NCM electrode was collected from the disassembled cycled coin cell, washed with DEC, and fully dried under an Ar atmosphere.

Comment 4

SEM, TEM, EDS visualisation of the morphology of the pristine and cycled electrodes depending on the binder are thoroughly used to assess the advantages of the new binder. It is well known that such observations are subjective and dependent on the sampling. However,

only one image is generally shown per technique and sample (eg. in Fig. 2a-c and in Fig. 5 a-i and in Fig. S2). Complementary images taken at various spots of the sample must be shown in supporting information as element of proof. At least 2 more per characterization techniques.

Our response

We greatly appreciate your valuable comments on our work. In response to the reviewer's comments, complementary images of SEM, TEM, and EDS visualization corresponding to Fig. 2a–f, 5a–j, and S2 have been provided as **Fig. R2–7**. These supplementary data exhibit trends consistent with those shown in our manuscript, further demonstrating the reproducibility and reliability of our results.

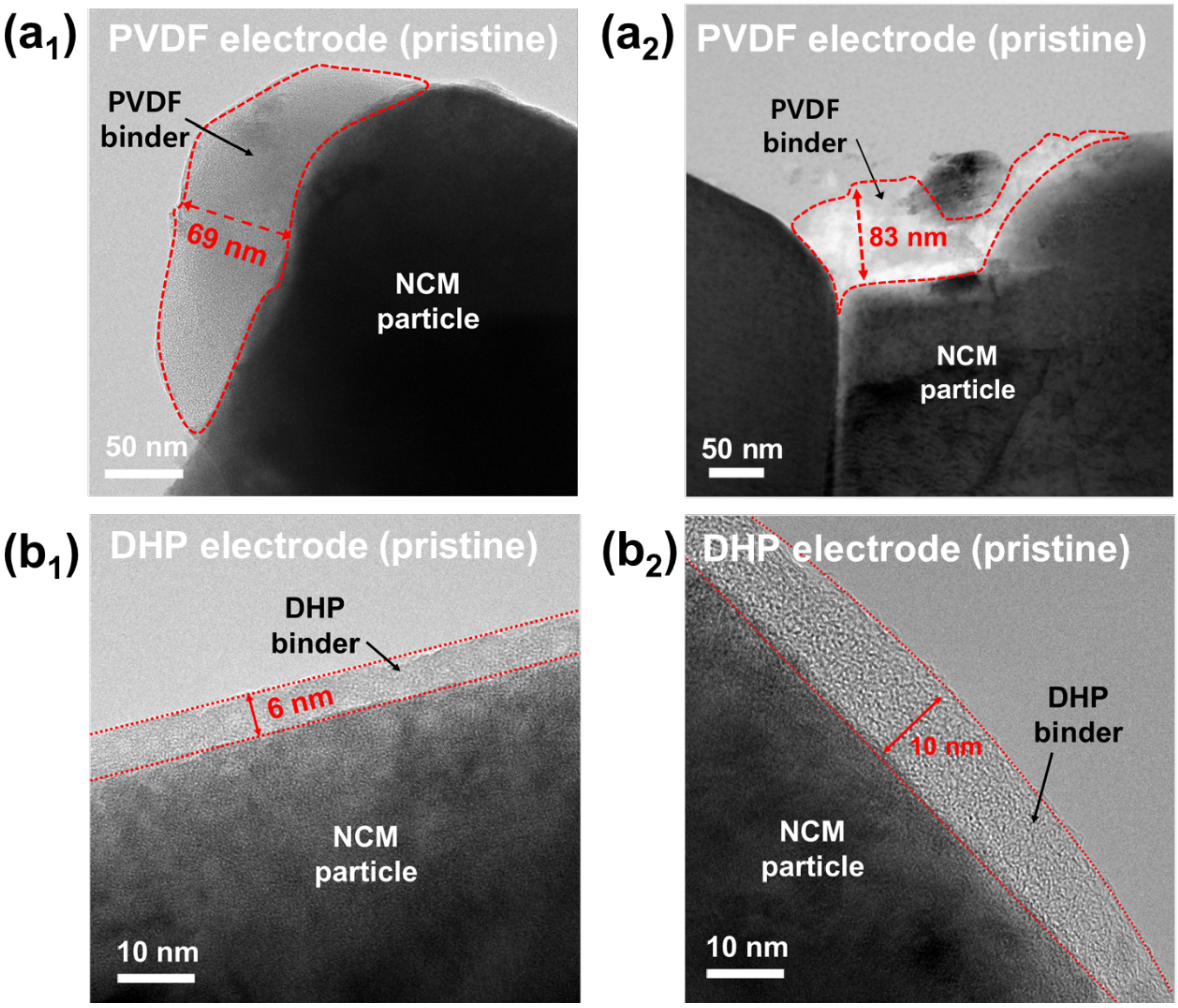

Fig. R2. Complementary TEM images of the binder coverage on NCM particles in pristine electrodes using (a_1, a_2) PVDF and (b_1, b_2) DHP electrodes corresponding to Fig. 2a and 2d.

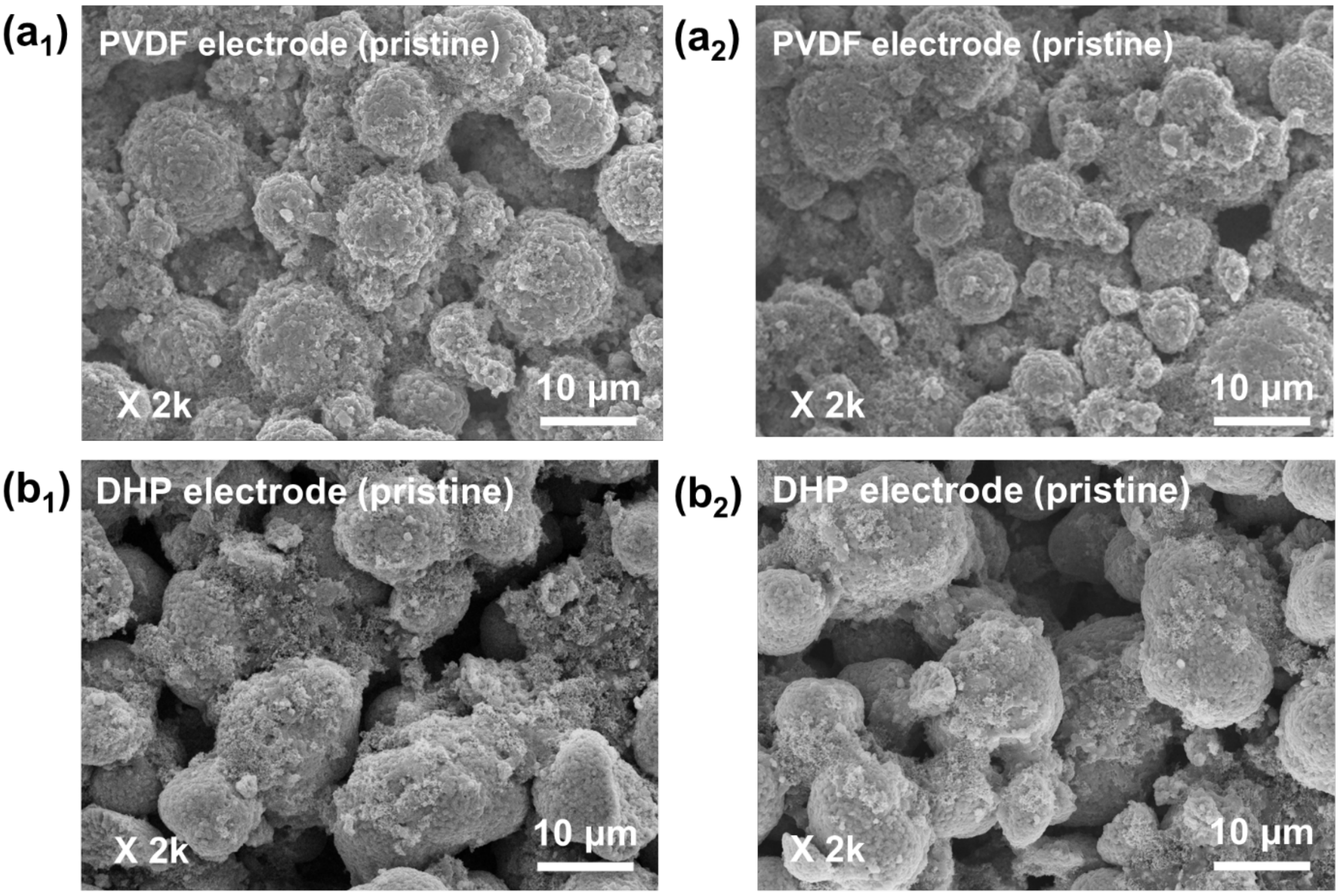

Fig. R3. Complementary Top-view SEM images of the pristine electrodes using (a_1, a_2) PVDF and (b_1, b_2) DHP electrodes corresponding to Fig. 2b and 2e.

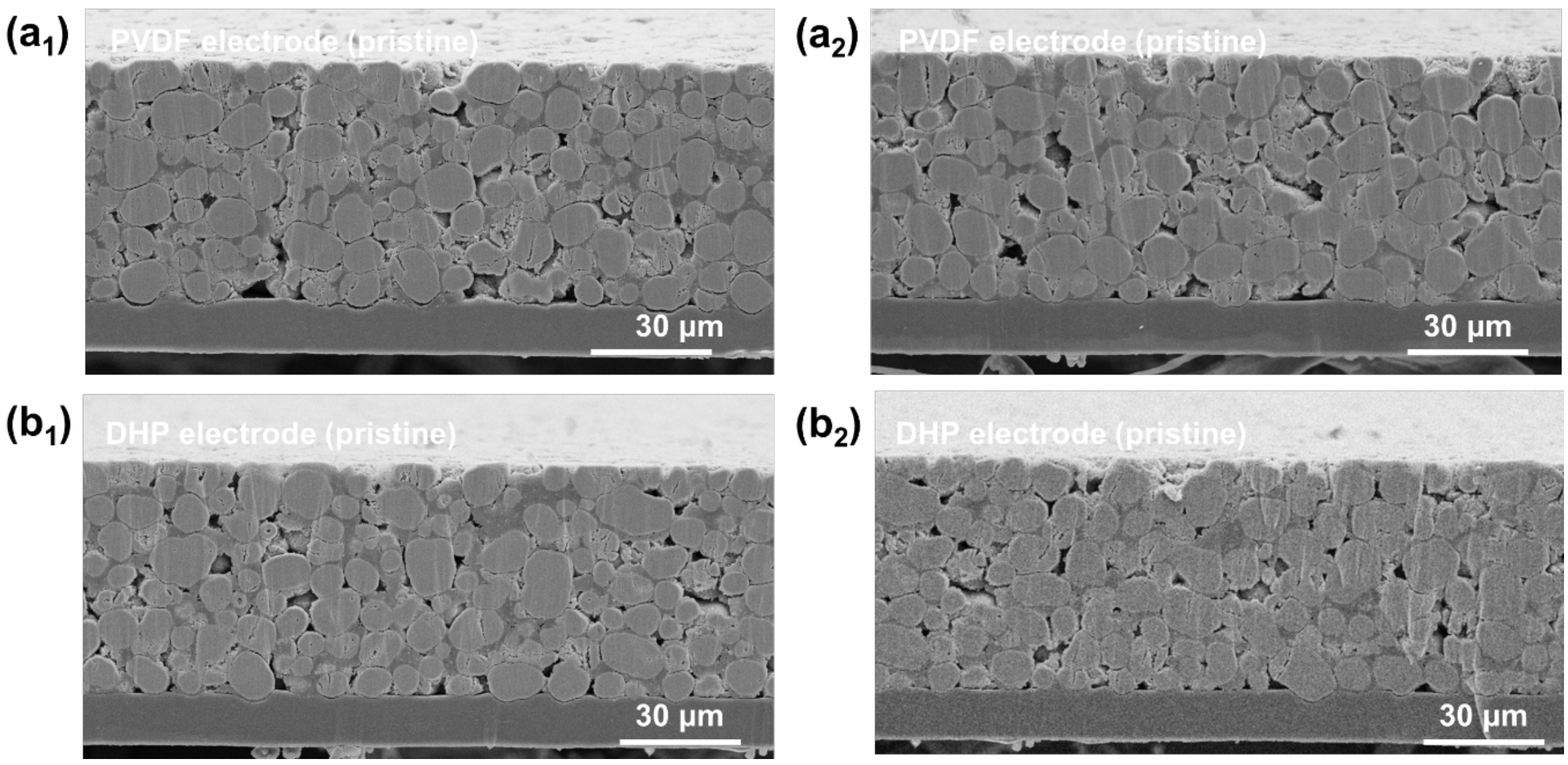

Fig. R4. Complementary Cross-sectional SEM images of the pristine electrodes using (a_1, a_2) PVDF and (b_1, b_2) DHP electrodes corresponding to Fig. 2e and 2f.

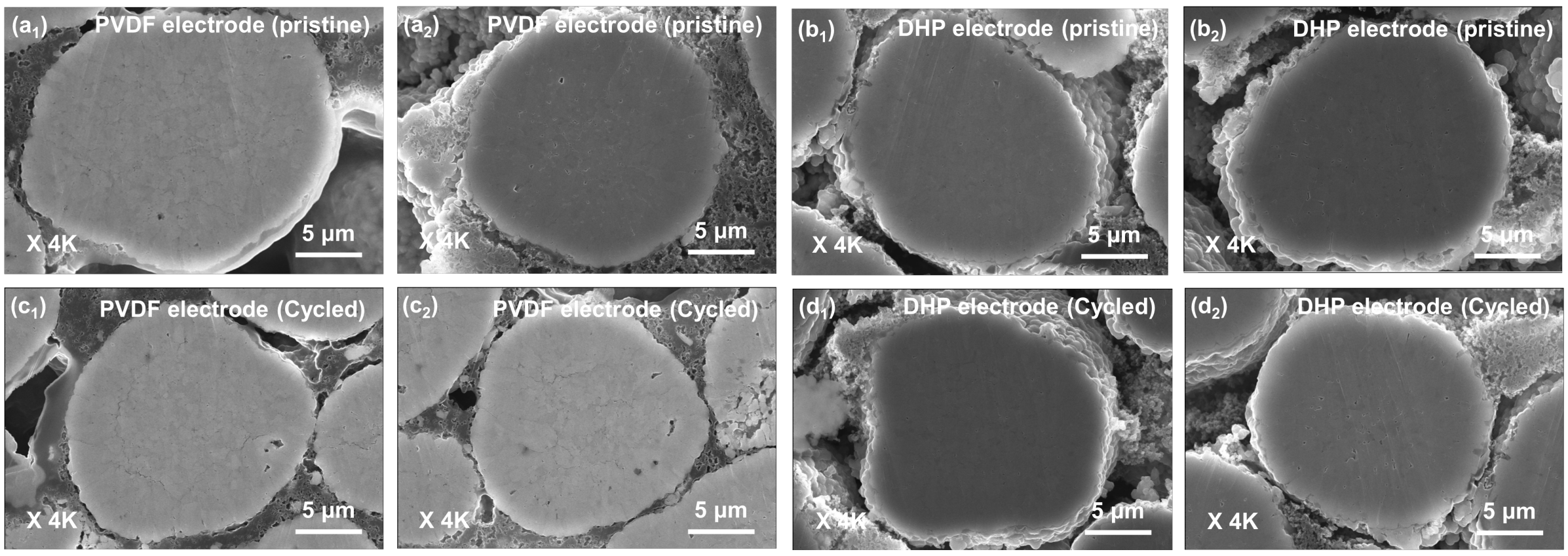

Fig. R5. Complementary SEM images of the pristine Ni-rich cathodes using (a_1, a_2) PVDF and (b_1, b_2) DHP and the cycled Ni-rich cathodes using (c_1, c_2) PVDF and (d_1, d_2) DHP corresponding to Fig. 5a-d.

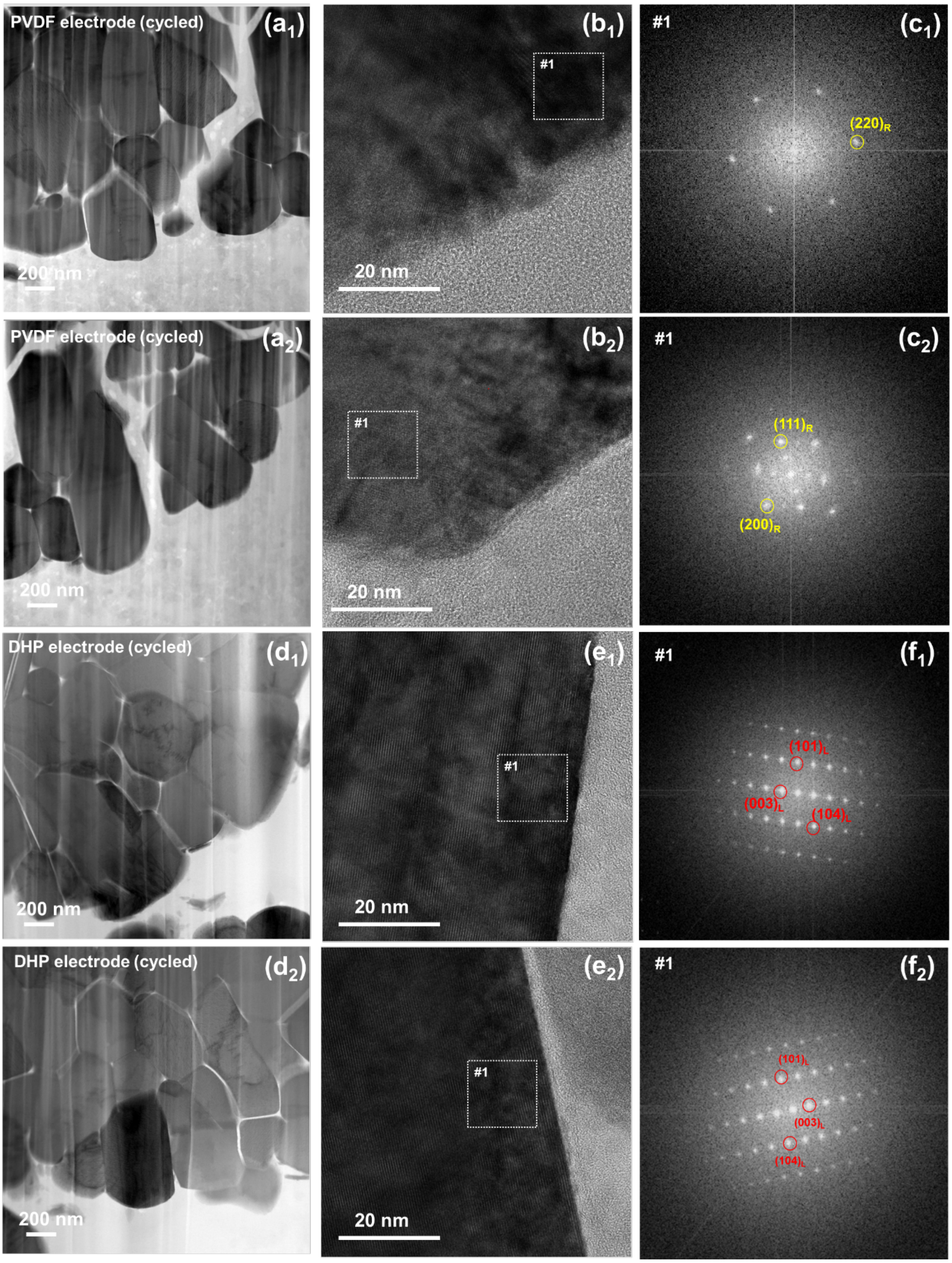

Fig. R6. Complementary STEM images of the cycled NCM in (a_1, a_2) PVDF- and (d_1, d_2) DHP-based cathode corresponding to Fig. 5e, f. High-resolution transmission electron microscopy (HR-TEM) images of the cycled NCM in (b_1, b_2) PVDF- and (e_1, e_2) DHP

-based cathode corresponding to Fig. 5g, h. Corresponding fast Fourier transform (FFT) patterns of the cycled NCM in (e_1, e_2) PVDF- and (f_1, f_2) DHP-based cathode corresponding to Fig. 5i, j.

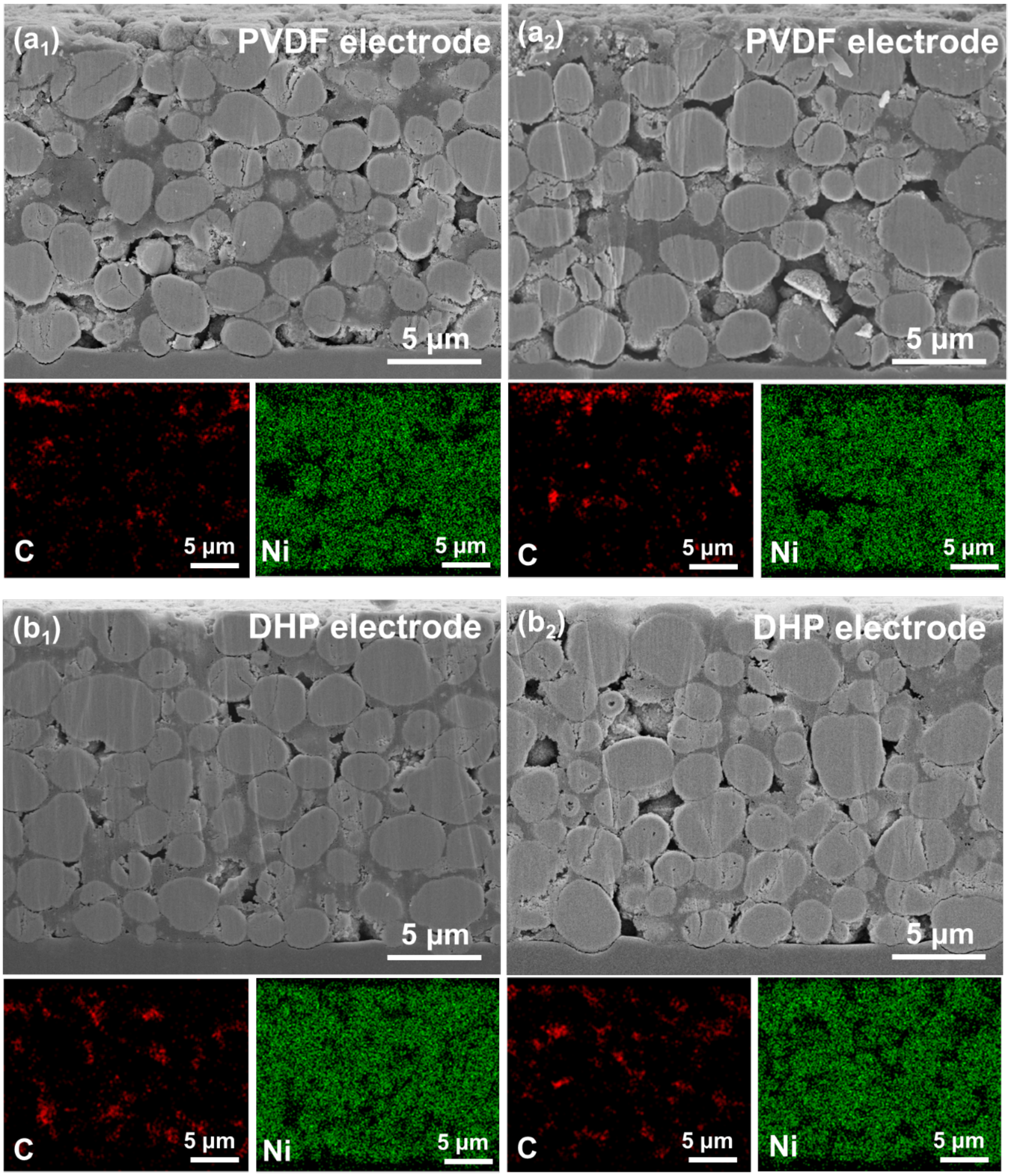

Fig. R7. Complementary Cross-sectional SEM images of the NCM cathodes prepared with (a_1, a_2) PVDF and (b_1, b_2) DHP binders and the corresponding EDS maps

corresponding to Fig. S2.

Comment 5

In Figure S2 the electrodes appear non porous or having very few porosities. What are the electrodes porosities? Is it possible to measure the distribution in pore size?

Our response

Thank you for your valuable comments. In this study, the NCM electrodes were fabricated by roll pressing to increase the electrode packing density to 3.3–3.4 g cm^{-3}. Although the roll-pressed electrodes in Fig. S2 may appear to have lower porosity, this density level is within the typical industrial range, where electrode packing densities of 3.2–3.6 g cm^{-3} or higher are commonly targeted to maximize volumetric energy density [R1-2]. In response to the reviewer's comments, we attempted to analyze the pore size distribution using mercury intrusion porosimetry (MIP), which is suitable for characterizing electrode-scale macro- and microporosity. However, due to the recent strict enforcement of international mercury regulations, MIP measurements could not be conducted at our university or affiliated research institutes. Instead, the electrode porosity was theoretically estimated based on the measured roll-pressed electrode density of 3.3–3.4 g cm^{-3}. Based on the true densities of NCM811 (ρ_{NCM}, 4.8 g cm^{-3}), Super P ($\rho_{super\ P}$, 2.0 g cm^{-3}) and DHP (ρ_{binder}, PAA: 1.3 g cm^{-3}; SPDX: 1.20 g cm^{-3}), the electrode porosity (ε) was estimated to be approximately 24–27% at a roll-pressed electrode density of 3.3–3.4 g cm^{-3} using the following equations:

$$\frac{1}{\rho_{solid}} = \frac{0.95}{\rho_{NCM}} + \frac{0.02}{\rho_{binder}} + \frac{0.03}{\rho_{super\ P}}$$

$$\varepsilon = 1 - \frac{\rho_{composite}}{\rho_{solid}}$$

This porosity range of the DHP electrode is similar with dense, high-areal-capacity cathodes reported in the literature and is sufficient to maintain continuous ionic transport pathways within the electrode. We once again appreciate the reviewer's valuable comments, which have contributed to improving the quality of our work.

[R1] Lim, J. et al. Validating the virtual calendering process with 3D-reconstructed composite electrode: An optimization framework for electrode design, *Small* **21**, 2410485 (2025).

[R2] Kwon, O. et al. Rational design of electrode mesostructures with integration of polycrystalline and single-crystalline Ni-rich particles, *Energy Storage Mater.* **84**, 104811 (2026).

Comment 6

In Figure 3b, one see a strong peak during the 1st lithiation for the new binder that is attributed to an in situ ion exchange reaction H+/Li+ occurring between the NCM cathode and the PAA of the new binder. Could the authors calculate the amount of electric charges associated (consummed) with this peak and relate it to the amount of available H+ from the -COOH moities of PAA (given its mass quantity in the electrode composition) to assess their interpretation ?

Our response

We greatly appreciate your valuable comments on our work. As the reviewer pointed out,

comparing the amount of electric charge associated with the CV peak to the theoretical amount of –COOH groups in PAA provides important quantitative support for Li-PAA formation. Accordingly, we first theoretically calculated the theoretical lithium consumption associated with complete conversion of –COOH groups to Li-PAA via the H^+/Li^+ exchange reaction. Assuming that all –COOH groups in PAA (accounting for approximately 0.66 wt% of the DHP electrode) are converted in a 1g batch of DHP electrode, and considering the molar mass of 72.06 g mol^{-1} for the PAA repeating unit, the number of moles of –COOH group is:

$$n_{COOH} = \frac{0.006667}{72.06} = 9.25 \times 10^{-5}\, mol$$

Since 1 mol of electrons corresponds to 26.801 Ah mol^{-1}, the theoretical charge consumption based on the electrode mass can be calculated as:

$$Q = n \times 26.801\, Ah\, mol^{-1} = 2.48\, mAh$$

Therefore, the theoretical Li consumption corresponds to approximately 2.48 mAh g^{-1} (electrode). Although it is difficult to clearly separate and quantify the charge contribution solely associated with Li-PAA formation because the H^+/Li^+ exchange reaction peak overlaps with the initial delithiation peak of NCM, the proposed Li–PAA formation via H^+/Li^+ exchange during the first electrochemical cycle is supported by spectroscopic evidence. Specifically, XPS analysis confirms the formation of Li-PAA after the first cycle. Additionally, post-cycling FT-IR analysis further corroborated these results. As shown in **Fig. R8**, the pristine DHP electrode exhibits a –COOH peak originating from PAA. This peak disappears after the initial charging process, accompanied by the formation of a –COOLi peak at around 1550 cm^{-1}. Collectively, these results consistently support the interpretation that the formation of Li–PAA in the DHP electrode predominantly occurs during the electrochemical reaction in the first cycle.

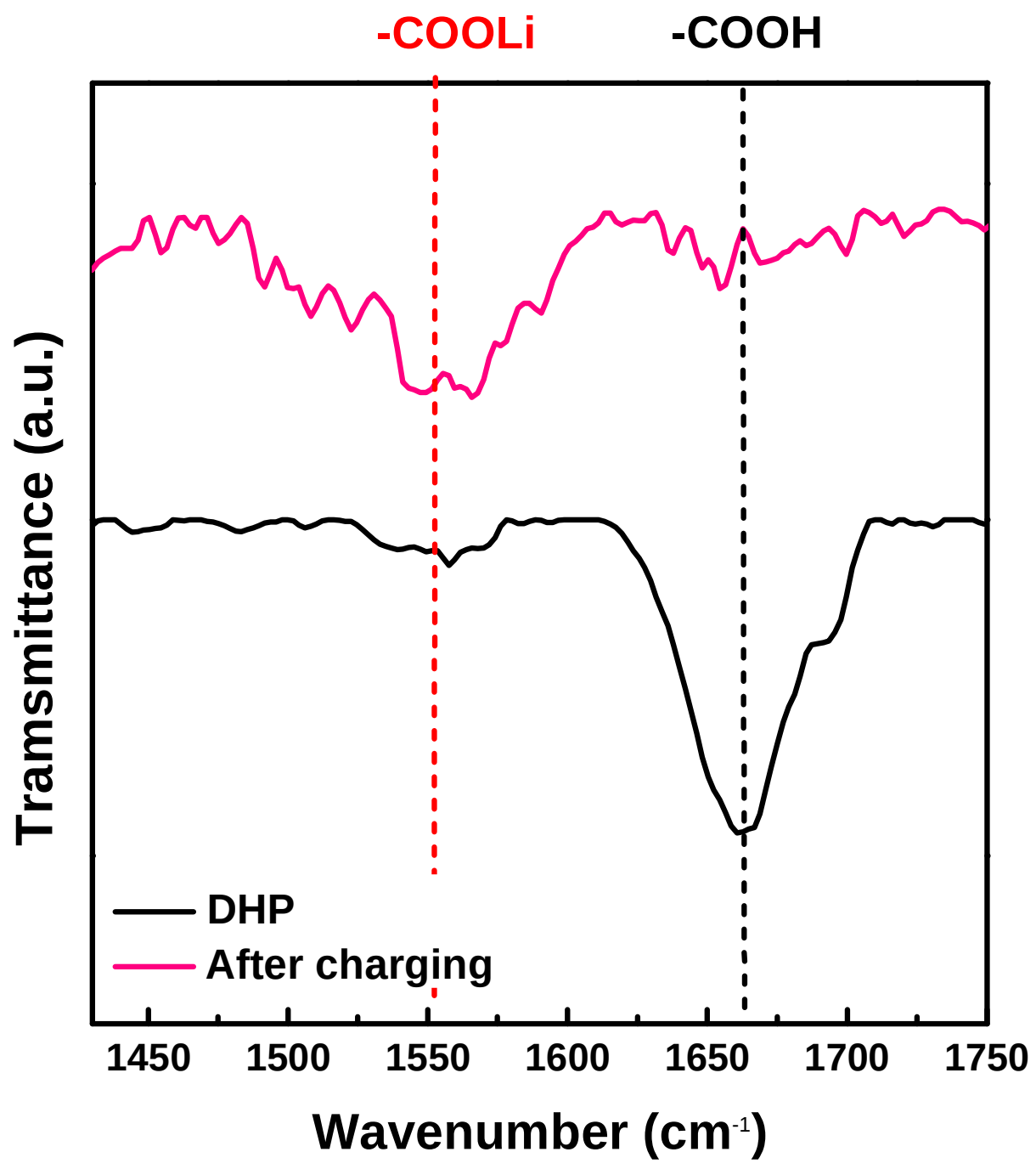

Fig. R8. FT-IR spectra of DHP and DHP electrodes after charging in the first cycle.

Comment 7

In Figure 3e-h, the intensity of the Li 1s peak is used to assess the formation of Li-PAA. The intensity of this peak must be sensitive to the XPS acquisition conditions, which is not described in the experimental method. Futhermore, what normalization procedure was adopted to secure a fair comparison between the samples ?

Our response

We greatly appreciate your valuable comments on our work. As noted by the reviewers, providing detailed information on the measurement conditions and normalization procedures of XPS analysis is essential for ensuring a clear interpretation of Li-PAA formation. In this study, for XPS analysis, the cycled NCM electrode was collected from disassembled cycled

coin cells, washed with DEC, and thoroughly dried under an Ar atmosphere. All NCM electrode samples were analyzed using Al Kα radiation under identical instrumental settings without additional Ar sputtering. Binding energy calibration was performed using the C–C bond peak of the C 1s spectrum at 284.8 eV. Peak fitting was conducted using Shirley background subtraction. We clarify the comparison of Li 1s signals in Fig. 3e-h based on relative intensity changes under identical acquisition conditions, rather than absolute quantitative comparison between independently acquired spectra. Since all samples were measured using the same instrumental parameters, the relative variation in Li 1s intensity provides meaningful information on the evolution of Li-containing species. To improve clarity and avoid confusion, the XPS data in Fig. 3 have been revised to present absolute intensities in cps, and a detailed description of the XPS measurement conditions has been added to the Experimental section. The revised Results and Discussion and Experimental sections are provided below.

Correction in the revised manuscript

1) Correction in Results and Discussion section.

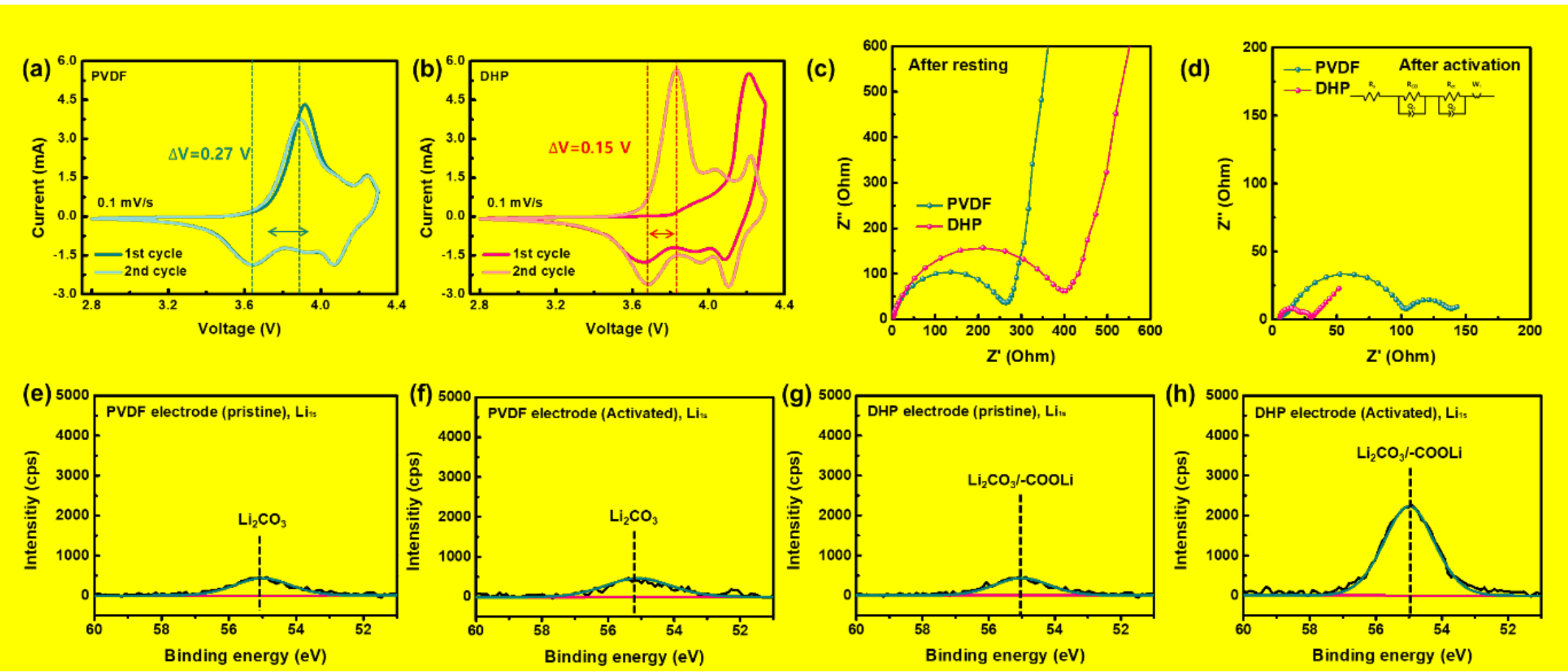

Fig. 3. Cyclic voltammetry (CV) curves of the Ni-rich cathodes using (a) PVDF and (d) DHP at the scan rate of 0.1 mV s^{-1}. Nyquist impedance plots of the Ni-rich cathodes using PVDF and DHP after (c) resting and (d) activation. Li 1s X-ray photoelectron spectroscopy (XPS) spectra of the Ni-rich cathodes using (b,c) PVDF and (e,f) DHP after activation.

2) Correction in Experimental section.

XPS (ESCALAB-250, Thermo Scientific, Al Kα) was utilized for surface composition analysis, and the binding energies of all XPS spectra were calibrated by referencing the C-C bond peak corresponding to C1s to 284.8 eV. HR-TEM (JEOL ARM-200F) provided atomic-resolution imaging at 200 kV. Raman spectroscopy (LabRamn Soleil, Horiba) analysis was conducted using a 532 nm laser over a wavenumber range of 350–650 cm^{-1} with a resolution of 2 cm^{-1}. For post-mortem analyses (XRD, XPS, SEM, TEM, and Raman), the NCM electrode was collected from the disassembled cycled coin cell, washed with DEC, and fully dried under an Ar atmosphere.

Comment 8

Figure 4b-c and 7g,j,k : reproducibility must be evaluated and results shown for at least 3 identical cells.

Our response

We greatly appreciate your valuable comments on our work. In response, we have revised the cell data in the manuscript to present the average values with corresponding standard deviations (SD) obtained from three independently assembled cells, thereby demonstrating the reproducibility of the electrochemical performance shown in Fig. 4b-c and Fig. 7g and j. Although additional pouch-cell evaluations were conducted to further verify reproducibility,

we were unable to complete full-cell cycling tests beyond 200 cycles within the revision period. Nevertheless, as shown in **Fig. R9**, the PVDF- and DHP-based electrodes exhibit electrochemical performance trends consistent with those presented with Fig. 7k, including comparable initial capacities and capacity retention behavior, which further supports the reproducibility of this study. Accordingly, the cell data have been revised to reflect reproducibility, and the revised 'Results and Discussion' and 'Experimental' sections are presented below.

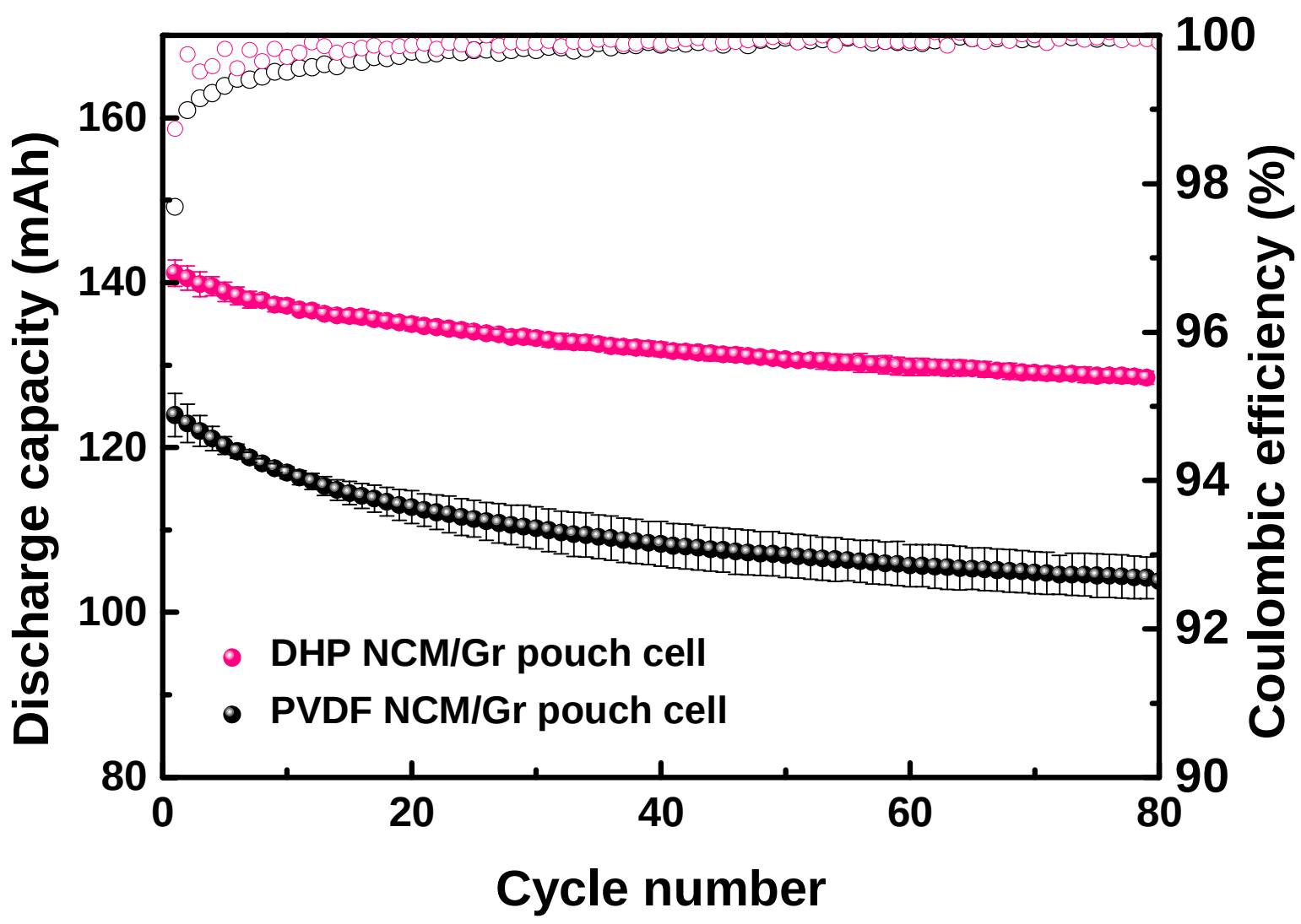

Fig. R9. Cycling performance of a DHP (40 mg cm^{-2}) ‖ graphite pouch cell, shown as the average and STD of three identical cells.

Correction in the revised manuscript

1) Correction in Results and Discussion section.

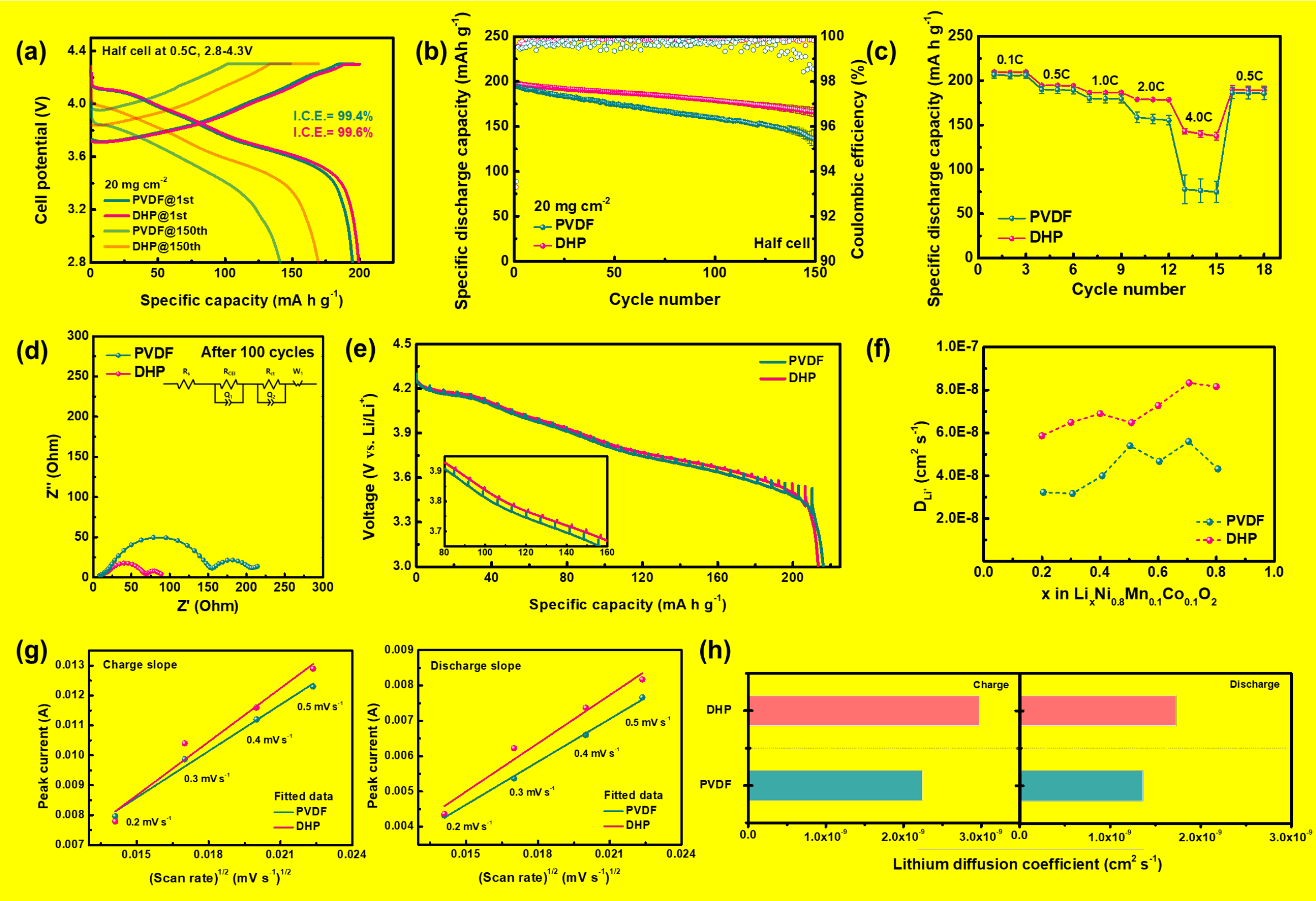

Fig. 4. (a) Voltage profiles of Ni-rich cathodes using the PVDF and DHP in their first and 150th cycle at 0.5 C (1 C = 185 mA g^{-1}). (b) Cycling performances of Ni-rich cathodes using the PVDF and DHP in the voltage window of 2.8–4.3 V when measured at 0.5 C. (c) Rate performance of both electrodes from 0.1 to 4 C in the voltage window of 2.8–4.3 V. (d) Nyquist plots of both electrodes after 100 cycles (e) Transient voltage profiles obtained by GITT during the discharge process. (f) D_{Li^+} of both electrodes as a function of the state of charge, which was estimated from the GITT. (g) Slopes of the redox peaks current of both electrodes from the CV results versus a square-root function of sweep rates. (h) D_{Li^+} of both electrodes, which was estimated from the CV results.

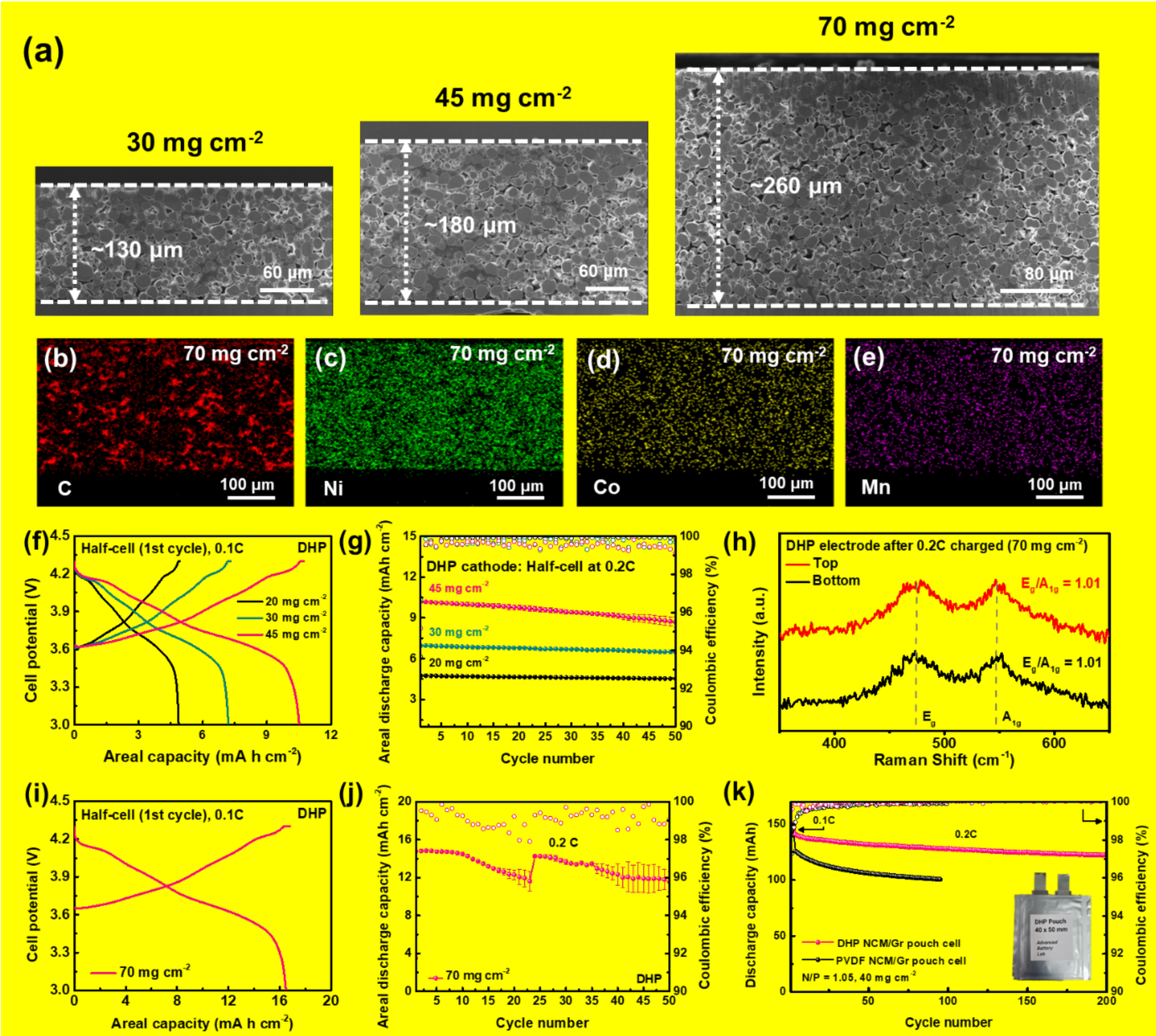

Fig. 7. High-mass-loading DHP electrodes. (a) Cross-sectional SEM images of the DHP electrodes with different areal mass loadings. Energy dispersive X-ray spectroscopy (EDS) images of the ultrahigh areal mass-loading DHP electrodes (70 mg cm^{-2}); (b) carbon, (c) nickel, (d) cobalt, (e) manganese. (f) Voltage profiles of the cells (DHP electrode||Li metal coin cell) as a function of the areal capacity at 0.1 C. (g) Cycling performance of the DHP electrodes with different areal mass loadings in the voltage window of 2.8–4.3 V measured at 0.2 C. (h) Raman spectra and the corresponding E_g/A_{1g} intensity ratio obtained from the top and bottom regions of the DHP cathodes with 70 mg cm^{-2}, after being charged to 4.3V (100% SOC) at 0.2 C-rate. (i) Voltage profiles of the cells (70 mg cm^{-2} DHP electrode||Li metal coin cell) as a function of the areal capacity at 0.1 C. (j) Cycling performances of electrodes with ultrahigh areal mass loadings. (k) Cycling performance of the DHP electrodes (40 mg cm^{-2})||Graphite pouch cell in the voltage window of 2.7–4.2 V measured at 0.2 C.

2) Correction in Experimental section.

Coin-type CR2032 cells were assembled in an argon-filled glovebox. A polyethylene (PE) separator (W-scope, 16 µm thickness) and an electrolyte consisting of 1 M $LiPF_6$ in EC/DEC (3:7 v/v) with 10 wt% FEC (PANAX ETEC) were used. For reproducibility, all coin cell tests were conducted in triplicate, and the results are presented as the mean values with standard deviations (SD). For the preparation of NCM/Gr pouch full cells, the NCM cathode was cut to dimensions of 40 mm × 50 mm, and the Gr anode was cut to 45 mm × 55 mm.

Comment 9

The satisfactory cyclability of DHP cathode half and full cells are shown. However, what are they rate performance (in particular for full cells) ? Indeed, there is little interest of achieving good cyclability of very high areal loading of electrodes if they cannont achieve good performance for example @1C.

Our response

We appreciate considerate comments on our work. According to reviewer comments, rate performance evaluations were conducted for NCM||Li half-cells and NCM||Gr full cells using 45 mg cm^{-2} thick electrodes with PVDF and DHP binder. As shown in **Fig. R10**a, in the half-cell test, the DHP electrode exhibited higher discharge capacities over the entire C-rate range. Specifically, while the PVDF electrode delivered 126.1 mAh g^{-1} (59.1% capacity retention) at 1.0 C rate, the DHP electrode maintained 163.4 mAh g^{-1} (76.0% capacity retention), demonstrating its superior rate capability. In the full-cell configuration (Fig. R10b), the DHP electrode also showed improved rate performance compared to the PVDF electrode. Although both electrodes exhibited substantial capacity fading at 1.0 C under high-areal-capacity conditions, the DHP electrode based full cell retained 42.4% of its low-rate capacity, which is

significantly higher than that of the PVDF electrode based full cell (24.5%). This relative improvement confirms that the DHP binder contributes to enhanced charge transport and structural stability even under high current densities. The reduced rate performance observed in the full cell configuration is primarily attributed to limitations associated with thick graphite anodes operating at high areal capacities (~11 mAh cm^{-2}), where sluggish ion transport and increased polarization can induce side reactions and lithium plating [R3-4]. Such effects are known to become more pronounced at high current densities, which explains why full cells employing graphite anodes exhibit lower discharge capacities than their corresponding half-cell counterparts. Overall, these results demonstrate that, despite the inherent challenges of ultra-thick electrodes, the DHP-based system consistently outperforms the conventional PVDF-based counterpart in both half- and full-cell configurations. We once again appreciate the reviewer's valuable comments, which have contributed to improving the quality of our work.

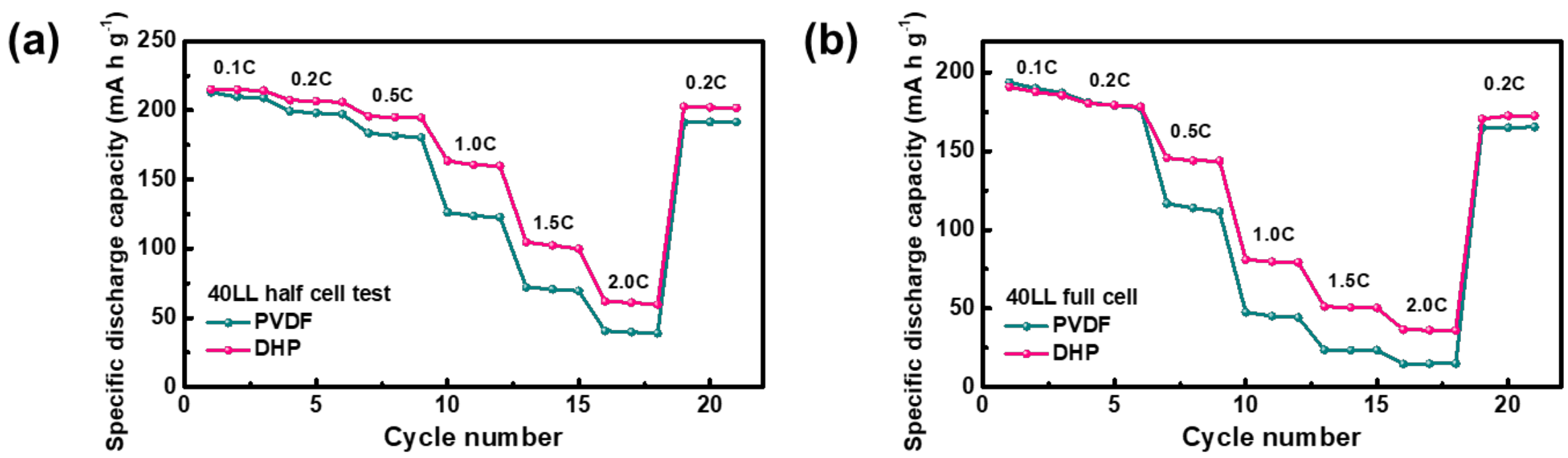

Fig. R10. Rate capability test of 45 mg cm^{-2} thick electrodes for (a) an NCM/Li half-cell and (b) an NCM/Gr full cell.

[R3] Yoon, J. et al. Deciphering the degradation mechanism of thick graphite anodes in high-energy-density Li-ion batteries by electrochemical impedance spectroscopy, *J. Ind. Eng. Chem.*

138, 424–431 (2024).

[R4] Ko, K.-H. et al. Degradation mechanism induced by depth-dependent inhomogeneity in thick high-areal-capacity graphite electrode, *Small* **21**, 2410795 (2025).

Comment 10

In their introduction, the authors cite the dry processing methods utilizing PTFE that can allow achieving very high areal capacity electrodes. Some very promising results were also obtained through such dry-processing methods but utilizing PVdF, which can be acknowledged, see for example J. A. Barreras-Uruchurtu, N. Besnard, C. Paul, L. Marchal, S. Devisme, B. Lestriez, "Effect of the physicochemical properties of PVdF on dry-sprayed graphite electrodes for lithium-ion batteries for electric vehicle applications", J. Electrochem. Soc., 2024, 171, 12053.

Our response

Thank you for your valuable comment. In response, we added relevant references on dry-processing methods utilizing PVdF and made minor revisions to the discussion of dry electrodes in the 'Introduction' section, as shown below.

Correction in the revised manuscript

1) Correction in Introduction section.

In addition, dry processing methods have recently garnered attention for fabricating hi gh-mass-loading electrodes; however, they still face industrial challenges such as incre ased complexity and cost, suggesting that they are far from completely replacing the

conventional wet process.[36,37,38,39]

Correction in the revised manuscript

2) Correction in Reference section.

38 Schumm, B. et al. Dry battery electrode technology: from early concepts to industrial applications. *Adv. Energy Mater.* **14**, 2406011 (2025).

39 Barreras-Uruchurtu, J. A. et al. Effect of the physicochemical properties of PVdF on dry-sprayed graphite electrodes for lithium-ion batteries for electric vehicle applications. *J. Electrochem. Soc.* **171**, 12053, (2024).

40 Chang, B. et al. Highly elastic binder for improved cyclability of nickel-rich layered cathode materials in lithium-ion batteries. *Adv. Energy Mater.* **10**, 2001069 (2020).

Comment 11

The peel strength results must be given in N/m or N/cm.

Our response

Thank you for your valuable comments. We performed the peeling tests using electrode samples with a fixed width of 1 cm. As suggested by the reviewer, to ensure quantitative consistency and enable fair comparison, we normalized the peeling strength values by specimen width and expressed them in units of N m^{-1} in Fig. 2g and Fig. S12. In addition, detailed information regarding the specimen dimensions and normalization procedure has been added to the Experimental section. The revised Results and Discussion, Experimental Section, and Supporting Information have been modified accordingly, as provided below.

Correction in the revised manuscript

1) Correction in Results and Discussion section.

The DHP electrode exhibited the highest average peeling strength (218 N m^{-1}), nearly twice that of the PVDF electrode (131 N m^{-1}), and even higher than that of the SPDX electrode (200 N m^{-1}), which is well known for its strong adhesion.

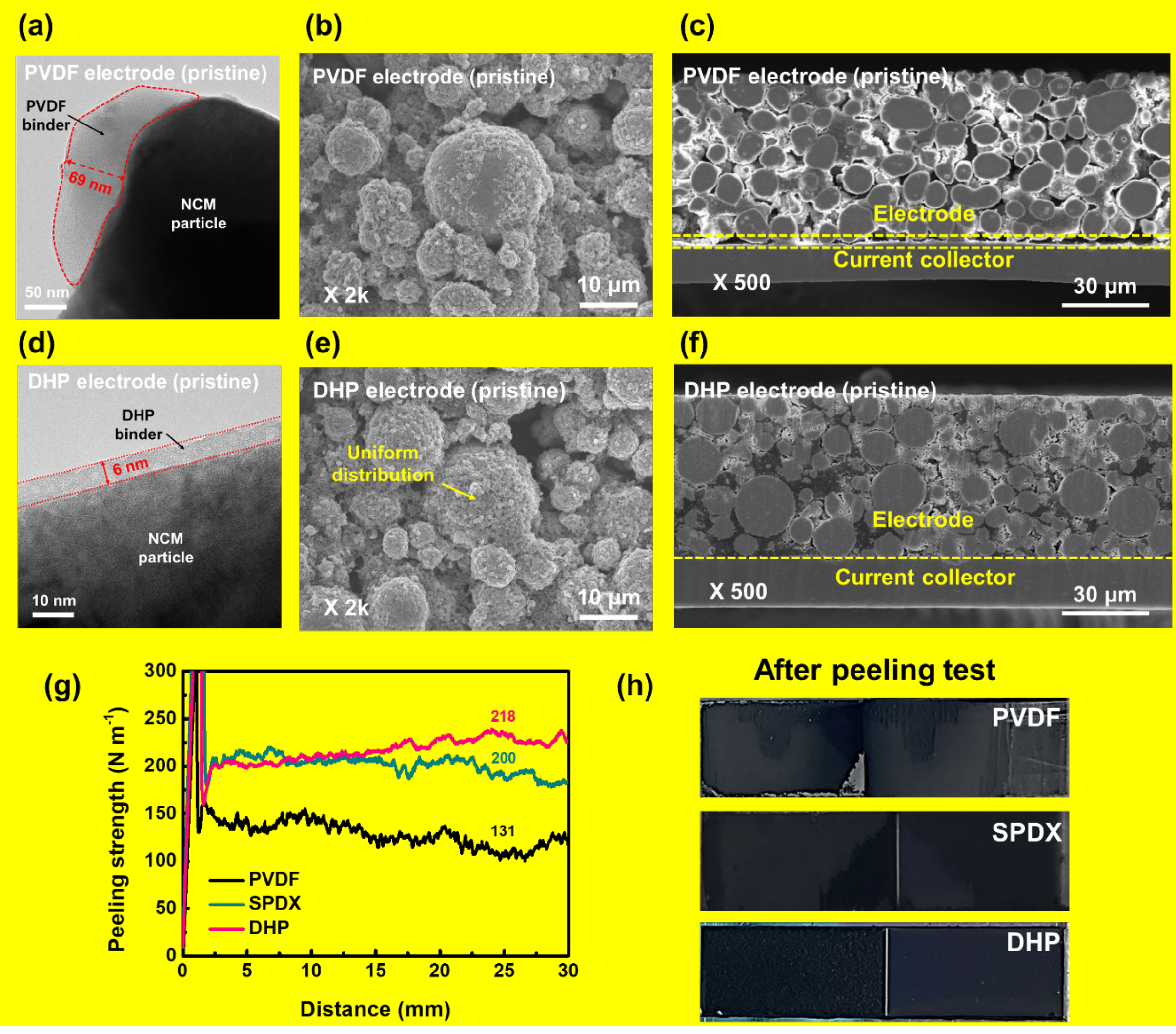

Fig. 2. Transmission electron microscopy (TEM) images of the binder coverage on NCM particles in pristine electrodes using (a) PVDF and (d) DHP. Top-view SEM images of pristine electrodes using (b) PVDF and (e) DHP. Cross-sectional SEM images of the pristine electrodes using (c) PVDF and (f) DHP. (g) 180° peeling test curves of the PVDF-, SPDX- and DHP-based electrodes. (h) Photographs after the peel test of the PVDF, SPDX and DHP electrodes.

2) Correction in Supporting information.

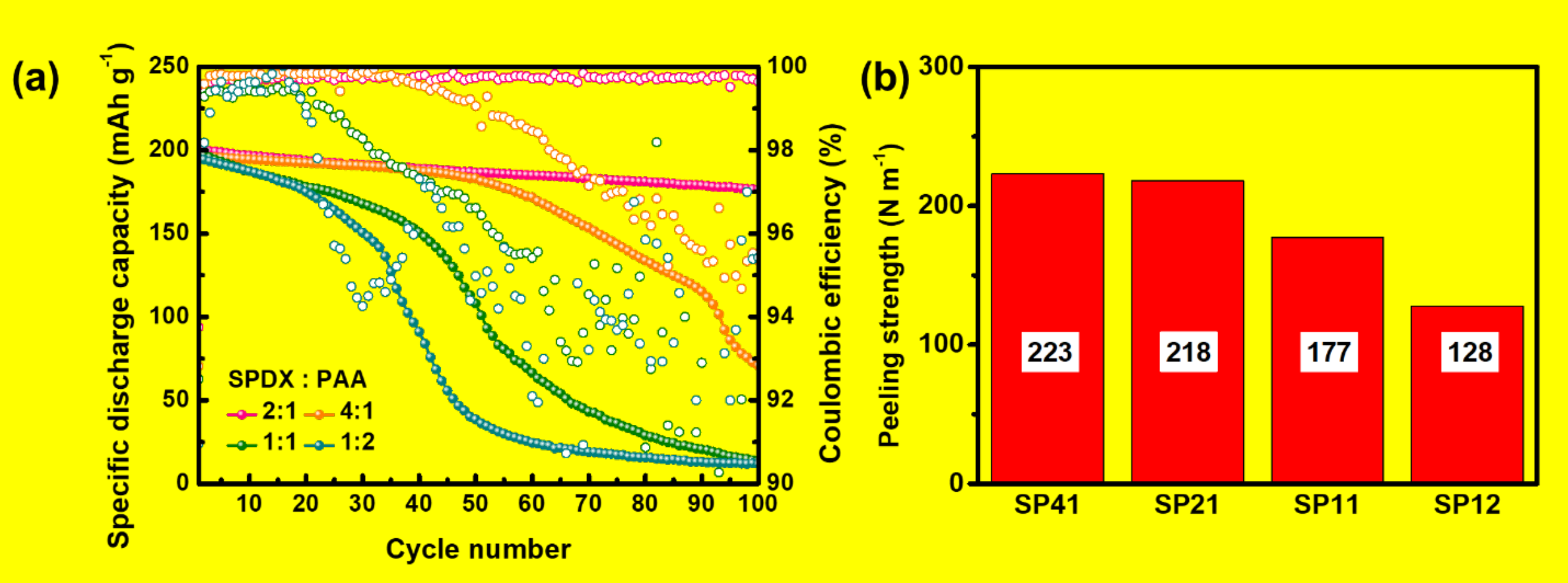

Fig. S12. (a) Cycle performances and (b) 180° peeling tests of DHP electrodes prepared using various combinations of SPDX and PAA binders.

3) Correction in Experimental section.

Characterization: Mechanical characteristics of the electrodes were assessed using a universal testing machine (UTM, JSV-H1000), including 180° peel tests using 3M double-sided tape at a peel rate of 25 mm min^{-1}. All samples used for the peel tests were prepared with a fixed width of 1 cm, and peeling strength normalized by sample width (N m^{-1}). FT-IR spectroscopy (TEMSOR27, Bruker, Germany) was employed to investigate the polymeric bonding structures. Electrode morphology was observed through FE-SEM (SU8010, HITACHI) and cross-sectional imaging via FIB (Helios 650, FEI, USA).

Comment 12

After 150 cycles, capacity retention of the PVDF electrode was measured to be 71.9%, which is low compared to the state of the art for a NMC811/PVdF electrode and may results from unoptimized preparation of the electrode or unsuitable PVdF grade. This should be discussed.

Our response

As pointed out by the reviewer, the cycling performance of the PVDF electrode in this study may be lower than that reported in some previous studies. We consider that this apparent discrepancy is largely attributed to differences in electrode loading level and applied C-rate, both of which are well known to strongly influence electrochemical performance. Whereas most recent studies employ PVDF-based NCM811 cathodes with loadings below 10 mg cm^{-2}, the PVDF electrode shown in Fig. 4b was fabricated with a higher loading of 20 mg cm^{-2}. As discussed in the manuscript, electrode loading is a critical parameter that significantly influences cell performance, thus performance differences under different electrode conditions are unavoidable. To clarify this point, we reviewed recent literature published over the past five years reporting Li metal half-cell tests of PVDF-based NCM811 electrodes under comparable loading and C-rate conditions. As summarized in **Table R1**, the cycling performance reported in these studies is comparable to, and in some cases lower than, that observed in the present work [R5-9]. Therefore, these comparisons indicate that the cycling performance of our PVDF electrode does not result from unoptimized electrode preparation or the use of an unsuitable PVDF grade.

	**Loading level of NCM811 cathode**	**Cycling performance**	**C-rate**	**Ref**
**1**	18.2 mg cm^{-2}	Failed at 55 cycles	0.5 C	[R5]
**2**	15-16 mg cm^{-2}	Failed at 120 cycles	0.5 C	[R6]
**3**	11 mg cm^{-2}	70.0%, 100 cycles	0.2 C	[R7]
**4**	18.8 mg cm^{-2}	28.9%, 80 cycles	0.5 C	[R8]
**5**	20 mg cm^{-2}	81.4%, 150 cycles	0.5 C	[R9]
**6**	20 mg cm^{-2}	82.3%, 100 cycles	0.5 C	Our work

71.9%, 150 cycles

Table R1. Comparison of the cycling performance of Li half cells with PVDF-based NCM cathode in the recently reported works.

[R5] Jeong, D. et al. Striking a balance: Exploring optimal functionalities and composition of highly adhesive and dispersing binders for high-nickel cathodes in lithium-ion batteries, *Adv. Energy Mater.* **13**, 2302845 (2023).

[R6] Jeong, Y. H. et al. A tough, adhesive, and protective binder shield for stabilizing high-nickel cathodes in lithium-ion batteries, *Adv. Energy Mater.* **15**, e02765 (2025).

[R7] Yan, X. et al. Synergistic sulfonyl–ether polyimide binders for stabilized high-loading NCM811 cathodes in high-energy lithium-ion batteries, *J. Mater. Chem. A* **13**, 38377–38387 (2025).

[R8] Han, D.-Y. et al. Ionically conductive elastic polymer binder for ultrahigh loading electrode in high-energy-density lithium batteries, *Adv. Mater.* **37**, 2506266 (2025).

[R9] Xiang, H. et al. High loading electrode with superior electron/ion transport network for high performance lithium-ion batteries, *J. Power Sources* **609**, 234672 (2024).

Comment 13

Page 14 : the authors write that the new binder suppresses the structural changes in NMC811, such as volume expansion during repeated lithiation and delithiation. This is not possible as lithiation and delithiation must result in volume expansion due to Li insertion and desinsertion

in the crystalline host structure of NMC811. However, the new binder may prevent the cracking mechanism in a certain way.

Our response

Thank you for your valuable comment. As noted by the reviewer, the proposed DHP binder cannot fundamentally suppress the intrinsic volume expansion associated with lithiation and delithiation. Rather, we intended to explain that DHP can mitigate the mechanical stress and cracking in NCM particles induced by repeated volume expansion and contraction during cycling. To clarify this point and avoid potential misunderstanding by readers, we have revised the relevant description in the Results and Discussion section of the manuscript, as shown below.

Correction in the revised manuscript

1) Correction in Results and Discussion section.

This structural degradation of the PVDF electrode suggests that severe microcracks developed in the NCM during continuous cycling, facilitating electrolyte penetration and accelerating the degradation of the cathode material.[11,52,53] These results demonstrate that the thin and uniform DHP coating layer formed on the surface of the cathode material particles effectively suppresses structural degradation of NCM 811, including microcracks, caused by volume expansion during repeated lithiation and delithiation. To confirm the effect of DHP on the phase transitions of NCM811, high-resolution TEM (HR-TEM) and fast Fourier transform (FFT) analyses were conducted on cathode cross sections after 100 cycles.

Responses to the comments by Reviewer #4:

This manuscript reports development of dual binder system to enable thick electrodes with improved performance. Below are some comments for improvement.

Comment 1

In the introduction section, the authors ascribed challenges in thick electrodes to binder migration during electrode drying. However, same process was used with the dual binder system. Why isn't the binder migration a problem any more?

Our response

We appreciate considerate comments on our work. The proposed DHP suppresses binder migration through two primary mechanisms. First, compared with PVDF, DHP forms a significantly high-viscosity slurry (380 Pa·s vs 106 Pa·s for the PVDF slurry at a shear rate of 250 s^{-1}, as shown in Fig. S1). Since binder migration is generally more severe in low-viscosity slurries due to enhanced convective flow, DHP is effective in reducing binder migration under the identical solvent conditions [R1]. Second, the functional groups of DHP can interact strongly with both the Al current collector and the electrode materials, providing enhanced adhesion and cohesion. These strong interactions improve binder immobilization during solvent evaporation, thereby mitigating capillary-driven redistribution and suppressing binder migration [R2,3]. Through these combined mechanisms, DHP effectively mitigates binder migration during the drying process, enabling the formation of a well-dispersed thick NCM electrode, as confirmed by SEM and TEM results.

[R1] Klemens, J. et al. Drying of NCM cathode electrodes with porous, nanostructured particles versus compact solid particles: Comparative study of binder migration as a function of drying conditions, *Energy Technol.* **10**, 2100985 (2022).

[R2] Chang, J. H. et al. Binder migration: Frequently observed yet overlooked phenomena in electrode processing for lithium-ion batteries, *J. Energy Storage* **83**, 110729 (2024).

[R3] Heo, S.-W. et al. Core-shell structure of polyacrylate-based binder enhancing the wet-adhesion strength and the kinetics of lithium-ion diffusion, *Electrochim. Acta* **455**, 142387 (2023).

Comment 2

The authors performed a series of postmortem analysis and ascribed the difference to the binder. However, how does the binder affect cation mixing in NMC?

Our response

Thank you for your valuable comment. As discussed in our work, the DHP binder can cover the NCM particle thinly and uniformly, and it has also excellent TM chelation ability owing to the abundance of carboxyl groups derived from PAA [R4]. Benefiting from these features, DHP can stabilize the NCM surface and mitigate cation mixing and transition-metal (TM) dissolution during prolonged cycling, consistent with the TEM and ICP-OES results presented in this work. Moreover, cation mixing can be accelerated by increased local overpotential arising from internal resistance and by the enlarged surface area due to surface cracking [R5]. In this regard, these degradation processes are effectively suppressed by the enhanced Li^+ transport and crack-suppression in the DHP electrode, thereby mitigating the progression of

cation mixing.

[R4] Liu, Z. et al. Poly(acrylic acid)-based polymer binders for high-performance lithium-ion batteries: from structure to properties. *Small* **20**, 2407297 (2024).

[R5] Su, J. et al. Multiscale failure mechanisms of ternary oxide cathode materials for lithium-ion batteries, *Adv. Mater.* **38**, e06063 (2026).

Comment 3

Miscroscopy images could be misleading. In Fig S2, there seem more particle cracks in the DHP electrode than that PVDF electrode, which is contradictory to the statement in the context.

Our response

We fully agree with the reviewer's concern regarding the particle cracks observed in the fresh DHP electrode. We consider that these cracks may have originated from the mechanical pressure applied during the roll-pressing process of the electrode fabrication. These cracks are identified at the uncycled state of the electrode rather than to post-cycling degradation and are therefore not related to the crack-suppression effect of DHP during electrochemical cycling discussed in this study. This appears to have resulted from an error during measurement within a specific area, as subsequent SEM analysis of the pristine DHP electrode did not reveal similarly severe cracking (**Fig. R1**). To avoid any confusion, the Supporting Information has been revised based on additional SEM analysis, as shown below.

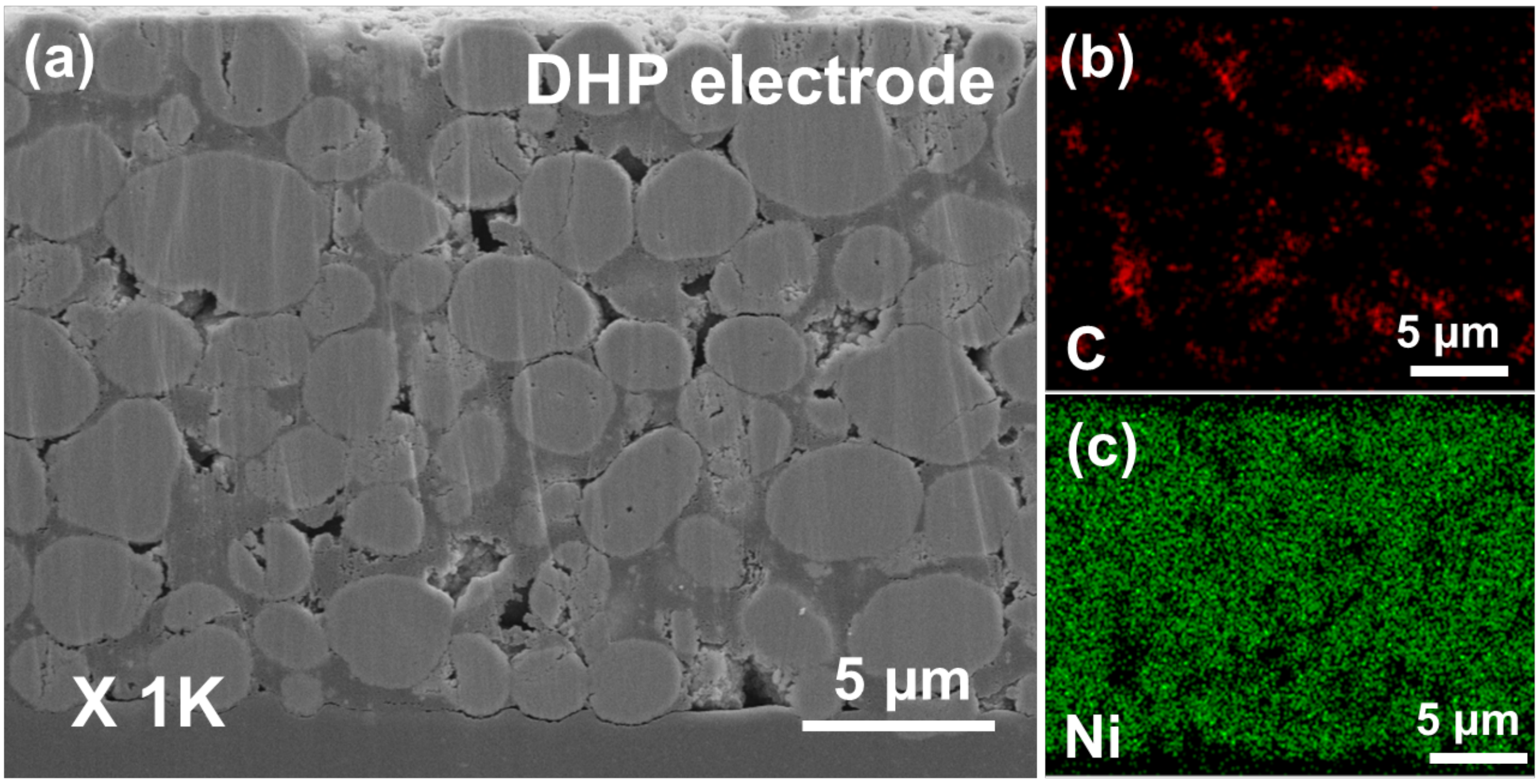

Fig. R1. Cross-sectional SEM images of the NCM cathodes prepared with (a) DHP binders and the corresponding EDS maps: (b) C (in red) and (c) Ni (in green).

Correction in the revised manuscript

1) Correction in Supporting information.

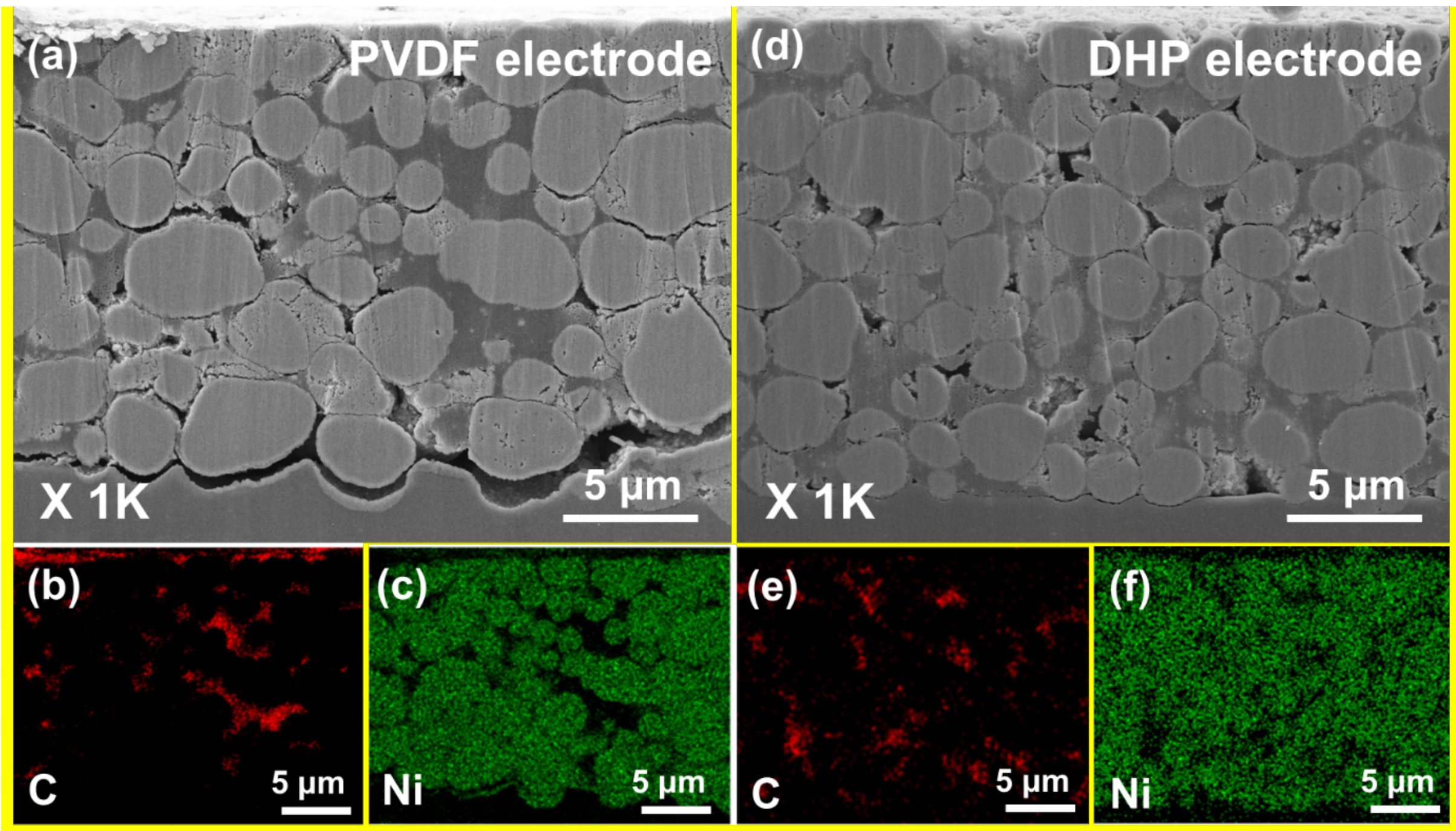

Fig. S2. Cross-sectional SEM images of the NCM cathodes prepared with (a) PVDF and (d) DHP binders and the corresponding EDS maps: (b), (e) C (in red) and (c), (f) Ni (in green).

Comment 4

The authors mentioned that NMC particles were fully covered by the dual binder. The electrodes would be more resistive similar to that after 100 cycles in figure 4d. However, why was the overpotential lower compared to the PVDF electrode as shwon in Fig 4a.

Our response

Thank you for your valuable comments. Upon reviewing the reviewer's comments and the results shown in Fig. 4d, we identified an error in the labeling of PVDF and SPDX, despite the trends of graph colors being clearly distinguishable. The DHP electrode exhibits lower R_{CEI} and R_{ct} values than the PVDF electrode in Fig. 4d owing to its highly Li^+-conductive Li–PAA interface and thus exhibits a lower overpotential in Fig. 4a. However, an error in Fig. 4d may have led to this misunderstanding. We sincerely apologize for any confusion this may have caused. To prevent potential misunderstandings by readers, we have revised the labeling in Fig. 4d of the manuscript, as shown below.

Correction in the revised manuscript

1) Correction in Results and Discussion section.

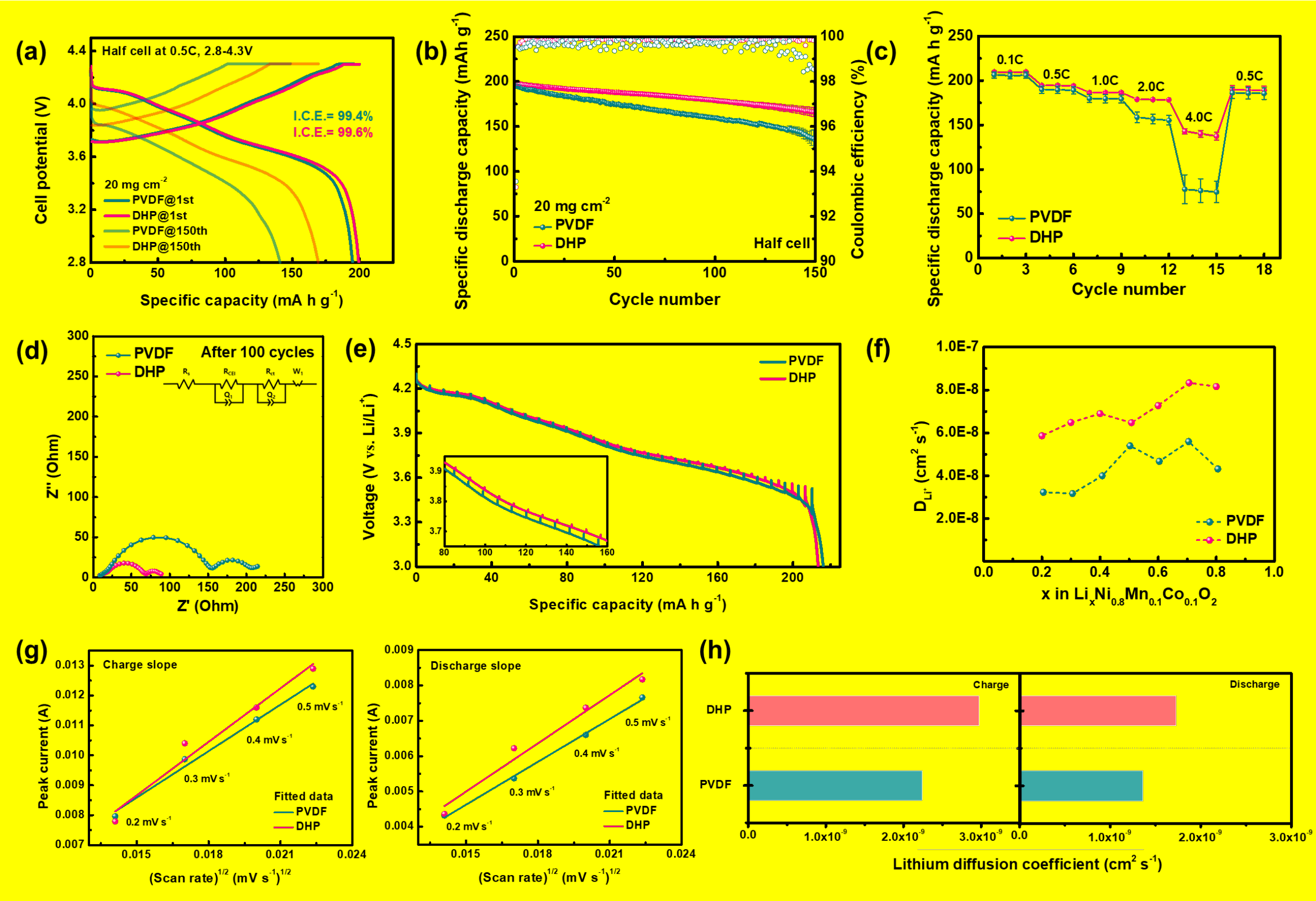

Fig. 4. (a) Voltage profiles of Ni-rich cathodes using the PVDF and DHP in their first and 150th cycle at 0.5 C (1 C = 185 mA g^{-1}). (b) Cycling performances of Ni-rich cathodes using the PVDF and DHP in the voltage window of 2.8–4.3 V when measured at 0.5 C. (c) Rate performance of both electrodes from 0.1 to 4 C in the voltage window of 2.8–4.3 V. (d) Nyquist plots of both electrodes after 100 cycles (e) Transient voltage profiles obtained by GITT during the discharge process. (f) D_{Li}^+ of both electrodes as a function of the state of charge, which was estimated from the GITT. (g) Slopes of the redox peaks current of both electrodes from the CV results versus a square-root function of sweep rates. (h) D_{Li}^+ of both electrodes, which was estimated from the CV results.

Comment 5

The impedance unit in Fig 4 d is incorrect.

Our response

We greatly appreciate your valuable comments. In response to the reviewer's suggestion, the impedance units in Figs. 3c–d and Fig. 4d have been revised from normalized to absolute values to avoid potential misunderstanding and improve readability, as shown below.

Correction in the revised manuscript

1) Correction in Results and Discussion section.

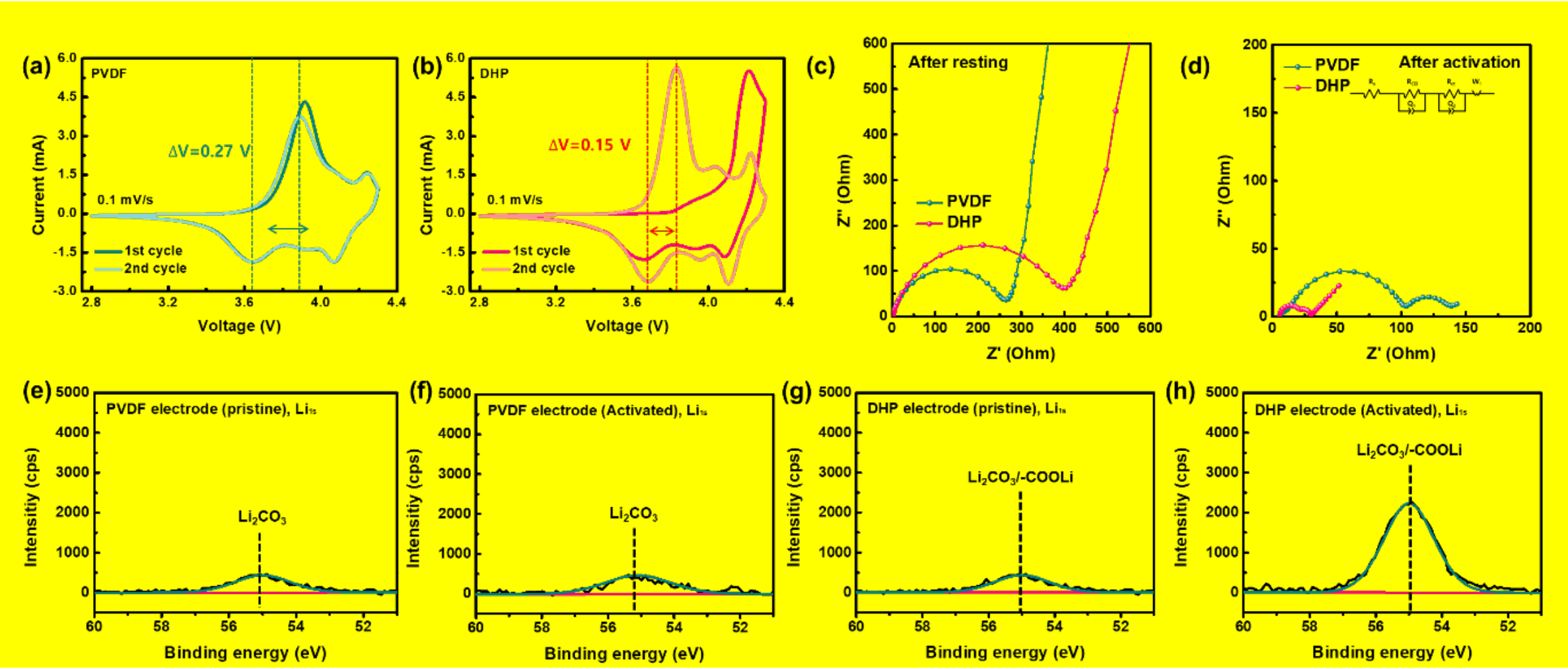

Fig. 3. Cyclic voltammetry (CV) curves of the Ni-rich cathodes using (a) PVDF and (d) DHP at the scan rate of 0.1 mV s^{-1}. Nyquist impedance plots of the Ni-rich cathodes using PVDF and DHP after (c) resting and (d) activation. Li 1s X-ray photoelectron spectroscopy (XPS) spectra of the Ni-rich cathodes using (b,c) PVDF and (e,f) DHP after activation.

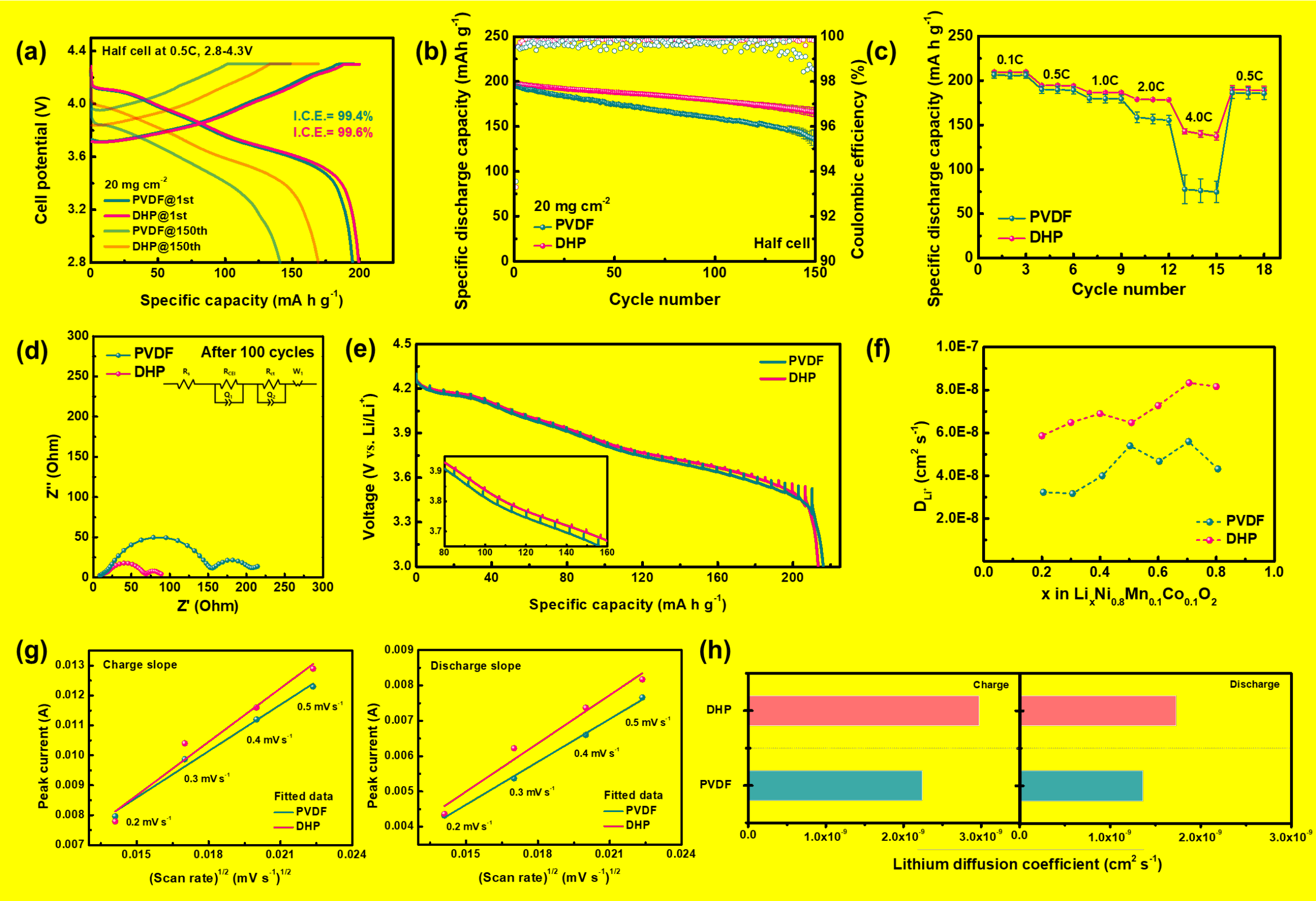

Fig. 4. (a) Voltage profiles of Ni-rich cathodes using the PVDF and DHP in their first and 150th cycle at 0.5 C (1 C = 185 mA g^{-1}). (b) Cycling performances of Ni-rich cathodes using the PVDF and DHP in the voltage window of 2.8–4.3 V when measured at 0.5 C. (c) Rate performance of both electrodes from 0.1 to 4 C in the voltage window of 2.8–4.3 V. (d) Nyquist plots of both electrodes after 100 cycles (e) Transient voltage profiles obtained by GITT during the discharge process. (f) D_{Li}^{+} of both electrodes as a function of the state of charge, which was estimated from the GITT. (g) Slopes of the redox peaks current of both electrodes from the CV results versus a square-root function of sweep rates. (h) D_{Li}^{+} of both electrodes, which was estimated from the CV results.

Comment 6

Since there Li-H exchange, what's the coulombic efficiency in the first cycle, particularly in full cells?

Our response

Thank you for your valuable comments. The pouch full cell with the DHP electrode delivered an initial coulombic efficiency (ICE) of 83.9%, which is slightly higher than that of the PVDF-based cell (82.2%) during the formation cycle (Fig. 7k). Although the H^+/Li^+ exchange reaction associated with Li–PAA formation consumes a small amount of lithium during the first cycle, the theoretical lithium consumption (approximately 2.48 mAh g^{-1} on an electrode basis) represents only a minor fraction of the total discharge capacity. Therefore, its contribution to irreversible capacity loss is limited. In contrast, the overall initial coulombic efficiency in thick full cells is strongly influenced by interfacial side reactions, charge-transfer resistance, and lithium transport limitations. The DHP binder promotes more uniform and efficient Li^+ transport within the thick electrode and provides thin, homogeneous surface coverage that suppresses excessive interfacial reactions. As a result, parasitic lithium consumption from side reactions is reduced. Consequently, despite the minor lithium consumption associated with Li-PAA formation, the DHP-based full cell exhibits slightly higher initial coulombic efficiency than the PVDF-based counterpart. These results indicate that the net electrochemical stabilization effect of the DHP binder outweighs the limited irreversible lithium consumption from the H^+/Li^+ exchange reaction.

Comment 7

Fabricating 45 mg/cm2 NMC cathode with PVDF as binder should not be a problem especially with doctor blade as shown in Fig. S7. The baseline was very poor.

Our response

We greatly appreciate your valuable comments on our work. The cracks observed in the 45 mg cm^{-2} PVDF electrode in Fig. S7 are likely attributable to the relatively low binder content (2 wt%) used in this study, compared to the 3–5 wt% binder content typically employed in previous reports. To further clarify this issue, additional 45 mg cm^{-2} NCM cathodes were fabricated using a doctor blade with PVDF binder contents of 1.5, 2, 3, and 4 wt%, and their surface morphologies were examined after drying. Consistent with the results shown in Fig. S7, localized cracks were observed in the electrodes containing 1.5 and 2 wt% PVDF (**Fig. R2**), whereas crack-free electrodes were obtained at binder contents of 3 and 4 wt%. Therefore, the surface cracking in PVDF electrode highlighted by the reviewer is associated with the low binder content rather than an inappropriate baseline selection. Importantly, the proposed DHP binder produced crack-free thick electrodes even at a binder content of 2 wt%, thereby demonstrating its excellent mechanical robustness and electrochemical performance.

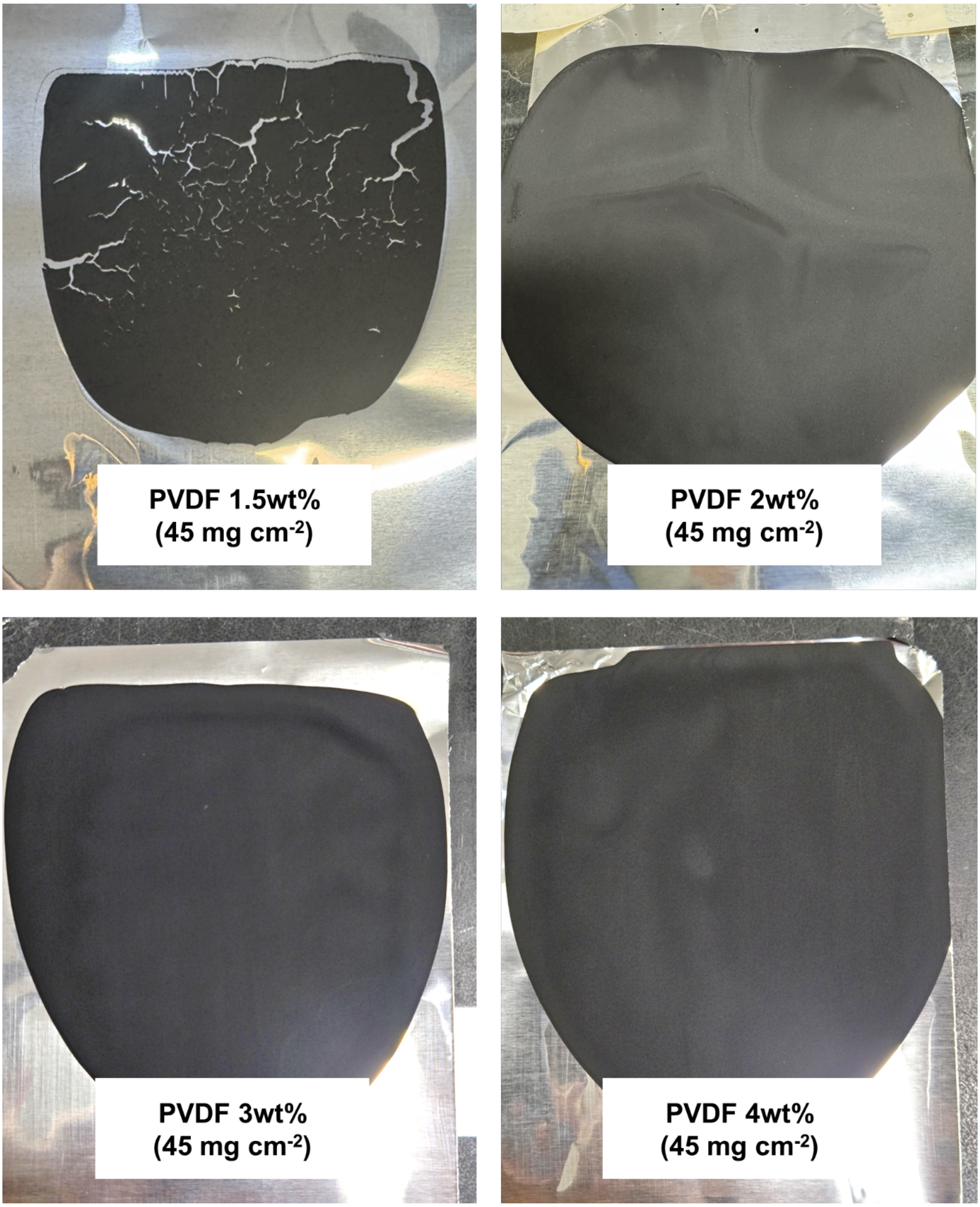

Fig. R2. Photographs of high-mass-loading (45 mg cm^{-2}) PVDF electrodes.

Comment 8

In Table S1, the tabs will be much larger for 10 Ah cells and should be much more heavier.

Our response

Thank you for your valuable comments. As the reviewer pointed out, the tab considered in the manuscript was thin and narrow to be suitable for a 10 Ah pouch cell. Therefore, considering the pouch capacity and dimensions, we revised the tab mass from 48.3 mg to 340 mg. Furthermore, we carefully reconfirm the calculations and cell design to ensure a more realistic pouch cell configuration. Specifically, the lithium metal thickness was reduced from 200 μm to 100 μm to achieve a realistic N/P ratio (2.58 to 1.24), the E/C ratio was revised from 1.7 to 2.0, and a minor error in the cathode weight calculation related to the current collector was corrected. Even after these corrections, the designed pouch cell still achieved an energy density of 474 Wh kg^{-1}. Accordingly, the Results and Discussion and Supporting Information sections have been revised as presented below.

Correction in the revised manuscript

1) Correction in Results and Discussion section.

The initial areal capacity was recovered, and stable cycling performance was maintained for up to 50 cycles, indicating that the observed capacity fading after 25 cycles originated from the Li metal anode degradation rather than the ultrahigh-mass-loading cathode. Moreover, as shown in **Table 1**, the estimated energy density of a 10 Ah-class pouch cell employing a DHP-based electrode with a mass loading of 70 mg cm^{-2} reached 474 Wh kg^{-1}, which is over 20% higher than that of the 25LL electrode (391 Wh kg^{-1}) (Calculation details are provided in Supplementary Table 1).

2) Correction in Supporting information.

Table S1. Gravimetric energy density of the fabricated >10 Ah-class pouch cell (calculated as an estimated value).

**Loading level of active material**	**Stack**	**Areal capacity (mAh cm^{-2})**	**Capacity (mAh)**	**M_c (mg)**	**M_a (mg)**	**M_s (mg)**	**M_p (mg)**	**M_e (mg)**	**M_t (mg)**	**Specific energy (Wh kg^{-1})**
**25**	91	5.55	10,101	51,560	20,381	1,950	1043	20,202	340	391
**70**	33	15.5	10,230	49,935	7,383	707	1043	20,460	340	474
